# Oncogenic stress‐induced Netrin is a humoral signaling molecule that reprograms systemic metabolism in *Drosophila*

**DOI:** 10.15252/embj.2022111383

**Published:** 2023-05-04

**Authors:** Morihiro Okada, Tomomi Takano, Yuko Ikegawa, Hanna Ciesielski, Hiroshi Nishida, Sa Kan Yoo

**Affiliations:** ^1^ Physiological Genetics Laboratory RIKEN CPR Kobe Japan; ^2^ Laboratory for Homeodynamics RIKEN BDR Kobe Japan; ^3^ Graduate School of Biostudies Kyoto University Kyoto Japan; ^4^ Division of Developmental Biology and Regenerative Medicine Kobe University Kobe Japan; ^5^ Division of Cell Physiology Kobe University Kobe Japan

**Keywords:** *Drosophila*, inter‐organ communication, Netrin, oncogenic stress, Metabolism, Signal Transduction

## Abstract

Cancer exerts pleiotropic, systemic effects on organisms, leading to health deterioration and eventually to organismal death. How cancer induces systemic effects on remote organs and the organism itself still remains elusive. Here we describe a role for NetrinB (NetB), a protein with a particularly well‐characterized role as a tissue‐level axon guidance cue, in mediating oncogenic stress‐induced organismal, metabolic reprogramming as a systemic humoral factor. In *Drosophila*, Ras‐induced dysplastic cells upregulate and secrete NetB. Inhibition of either NetB from the transformed tissue or its receptor in the fat body suppresses oncogenic stress‐induced organismal death. NetB from the dysplastic tissue remotely suppresses carnitine biosynthesis in the fat body, which is critical for acetyl‐CoA generation and systemic metabolism. Supplementation of carnitine or acetyl‐CoA ameliorates organismal health under oncogenic stress. This is the first identification, to our knowledge, of a role for the Netrin molecule, which has been studied extensively for its role within tissues, in humorally mediating systemic effects of local oncogenic stress on remote organs and organismal metabolism.

## Graphical abstract

Ras‐induced tumors in *Drosophila* reduce organismal fitness by Netrin secretion‐dependent suppression of carnitine biosynthesis in the fat body.

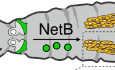

## Introduction

Why animals die from cancer is enigmatic. While cancer affects organs where it exists, cancer patients often exhibit systemic symptoms. For example, it has been long known that cancer patients tend to suffer from infection due to immunosuppression that often accompanies cancer (Bodey, 1986; Hanahan & Weinberg, 2011; Hiam‐Galvez *et al*, 2021). Cancer also induces cachexia, which is defined by loss of muscles and fat tissues (Argiles *et al*, 2014). Cancer cachexia occurs in more than 80% of patients with advanced cancer (Fearon *et al*, 2013). Metabolic dysfunction induced by tumor can be primary causes of cancer‐related morbidity and mortality (Egeblad *et al*, 2010). The cytokine storm has been postulated to mediate the cancer's systemic effects such as immunosuppression and cachexia (Luft, 2007; Argiles *et al*, 2014; Hiam‐Galvez *et al*, 2021), but its exact mechanism remains elusive.

Research with *Drosophila* has been pioneering discovery of molecular mechanisms of tumorigenesis by taking advantage of its powerful genetics (Wu *et al*, 2010; Villegas, 2019). Recently, *Drosophila* research started to address non‐cell autonomous effects of tumor on the host using both larvae and adults (Figueroa‐Clarevega & Bilder, 2015; Kwon *et al*, 2015; Song *et al*, 2019; Bilder *et al*, 2021; Santabarbara‐Ruiz & Leopold, 2021). The main advantage of using *Drosophila* for investigating tumor‐host interaction is its relative ease to independently manipulate genes in tumor and non‐tumor tissues. Previous studies implicated humoral factors such as ImpL2 (Figueroa‐Clarevega & Bilder, 2015; Kwon *et al*, 2015), Pvf1 (Song *et al*, 2019), and ecdysone (Santabarbara‐Ruiz & Leopold, 2021) in mediating tumor's systemic effects. Inhibition of these humoral factors, however, does not completely ameliorate the systemic phenotypes induced by oncogenic tissues, suggesting that there are additional mechanisms that mediate tumor's systemic effects on animals.

## Results

### NetrinB in Ras^V12^‐transformed tissues affects organismal lethality

**Figure 1:**
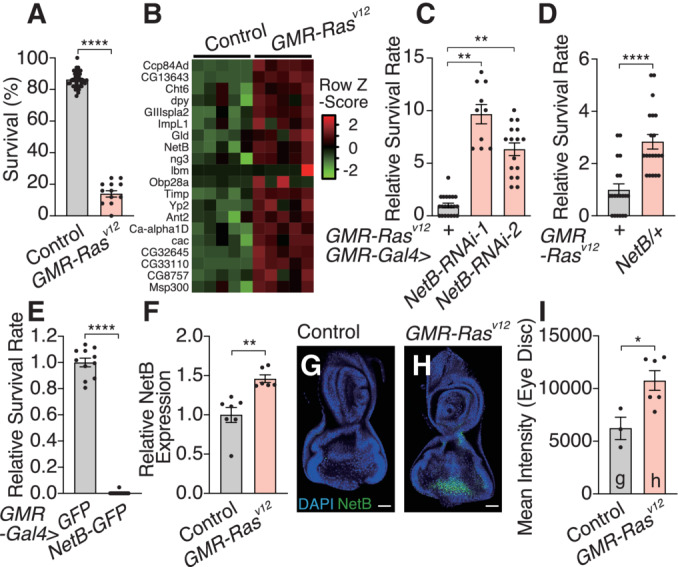
NetrinB in Ras^V12^‐transformed tissues affects organismal lethality AExpression of oncogenic *Ras* ^*V12*^ under the control of *GMR* enhancer element (*GMR*‐*Ras* ^*V12*^) leads to organismal lethality. The survival rate is calculated by counting the number of adult flies.BExpression of selected genes that were upregulated in the eye disc of *GMR‐Ras* ^*V12*^ flies.CKnockdown of *NetB* in the eye disc of *GMR‐Ras* ^*V12*^ flies enhances survival.D
*NetB* heterozygous mutant flies survive better over Ras^v12^‐induced oncogenic stress.EEctopic expression of *NetB* in the eye disc kills animals even without tumor formation in the eye.FqRT–PCR with mRNA from the eye disc confirms higher expression of *NetB* in *GMR*‐*Ras* ^*V12*^ flies.G, HOncogenic *Ras* expression in the eye disc induces NetB protein, which was detected by GFP signal using CPTI‐000748, a protein trap of NetB. Single confocal z‐section images are shown.IQuantification of GFP signals in (G) and (H). Data information: Data points indicate biological replicates. Data are mean ± s.e.m., and the statistical significance was determined by one‐way ANOVA followed by Dunnett's multiple comparisons test (C) and two‐tailed unpaired *t*‐test (A, D, E–F, and I). *P < 0.05, **P < 0.01, ****P < 0.0001. Scale bar, 50 μm. Source data are available online for this figure.

**Figure EV1:**
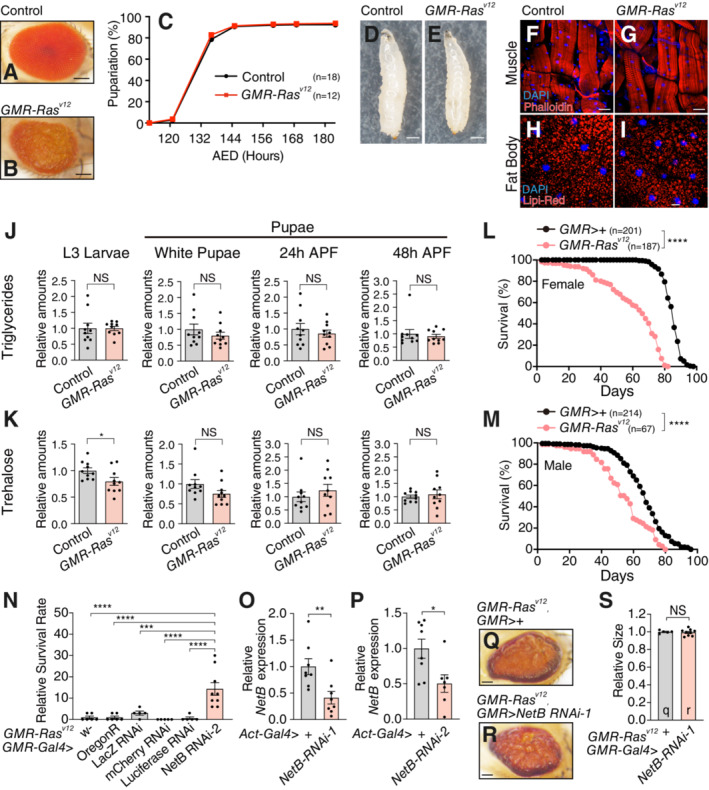
*GMR‐Ras* ^*V12*^ model and *NetB* expression A, BRepresentative images of adult eyes from Control (*OregonR*) (A) and *GMR‐Ras* ^*V12*^ flies (B).CNo developmental retardation for the timing of pupariation was observed in *GMR‐Ras* ^*V12*^ flies. The time for each larva to reach a pupal stage were determined and plotted. AED, hours after egg deposition.D, ERepresentative images of control (D) and *GMR*‐*Ras* ^*V12*^ third‐instar larvae (E). Scale bar, 500 μm.F, GPhalloidin and DAPI staining of dissected larval body‐wall muscle from control (F) and *GMR*‐*Ras* ^*V12*^ third‐instar larvae (G). Single confocal *z*‐section images. Scale bar, 50 μm.H, ILipi‐Red and DAPI staining of dissected fat body from control (H) and *GMR*‐*Ras* ^*V12*^ third‐instar larvae (I). Single confocal *z*‐section images. Scale bar, 20 μm. Note that no *GMR*‐*Ras* ^*V12*^ third‐instar larvae show the bloating symptom or degeneration of muscles/fat.J, KThe amounts of triglycerides (J) and trehalose (K) with the indicated genotypes during development. APF, after puparium formation.L, MSignificant decrease in *GMR‐Ras* ^*V12*^ adult female (L) and male (M) lifespan compared to control flies (*GMR > +*).NUAS‐RNAi expression does not affect the survival rate compared to w‐ and OregonR control.O, PqRT–PCR analysis of *NetB* RNAi efficiency. NetB RNAis lowered expression of *NetB* mRNA.Q, RRepresentative images of adult eyes from *GMR*‐*Ras* ^*V12*^ flies with (R) or without knockdown (Q) of *NetB* in the eye disc using *GMR*‐*Gal4*. Scale bar, 100 μm.SQuantification of the eye area in (Q) and (R). Data information: Data points indicate biological replicates. Data are mean ± s.e.m., and *n* represents the number of vials (C) of flies (L, M) that were analyzed. The experiments were repeated independently at least twice with similar results (A and B). The statistical significance was determined by one‐way ANOVA followed by Dunnett's multiple comparisons test (N), two‐tailed unpaired *t*‐test (J and K, O and P, and S), and log‐rank (Mantel‐Cox) test (L and M). NS, not significant, *P < 0.05, ****P < 0.0001. Source data are available online for this figure.

To understand how tumor affects organismal physiology and metabolism, we used *Drosophila* larvae, a genetically tractable system to study tumor biology (Dar *et al*, 2012; Bilder *et al*, 2021; Nishida *et al*, 2021; Santabarbara‐Ruiz & Leopold, 2021). To prevent an occurrence of a too strong malignant situation such as metastasis or massive overproliferation, which has a tremendous local effect, arresting development and confounding interpretation of the systemic effect, we decided to induce a relatively mild, pre‐cancer situation, in the eye imaginal disc, which is a dispensable organ for organismal survival. We expressed *Ras* ^*V12*^, which is the most common mutation and a prerequisite for many tumors (Prior *et al*, 2012; Hobbs *et al*, 2016), in the eye disc using the GMR enhancer element (Freeman, 1996; Tang *et al*, 2001). Ras expression in the eye disc leads to dysplasia, as previously demonstrated (Simon *et al*, 1991), which results in the “rough eye” in adults (Fig EV1A and B). Although this dysplastic tissue does not metastasize or affect the brain tissue, it leads to high lethality: over 80% of the *GMR‐Ras* ^*V12*^ animals die (Fig 1A). Most of *GMR‐Ras* ^*V12*^ larvae pupariate without developmental delay compared to control flies in spite of oncogenic stress in the eye imaginal disc (Fig EV1C). This is due to the late initiation of *GMR*‐driven Ras expression behind the morphogenetic furrow (Freeman, 1996), past the timing when stresses can delay developmental timing (Halme *et al*, 2010). In spite of the high lethality during development, animals carrying the *Ras* ^*V12*^‐transformed tissue do not display apparent cachexia symptoms such as the bloating phenotype (Fig EV1D and E), muscle (Fig EV1F and G) or fat body degeneration (Fig EV1H–J), or hyperglycemia (Fig EV1K), which are induced by more aggressive tumors (Figueroa‐Clarevega & Bilder, 2015; Kwon *et al*, 2015; Song *et al*, 2019; Newton *et al*, 2020; Ding *et al*, 2021; Khezri *et al*, 2021; Kim *et al*, 2021; Santabarbara‐Ruiz & Leopold, 2021). Survivors of *GMR‐Ras* ^*V12*^ flies have shorter lifespan in both males and females (Fig EV1L and M).

**Figure EV2:**
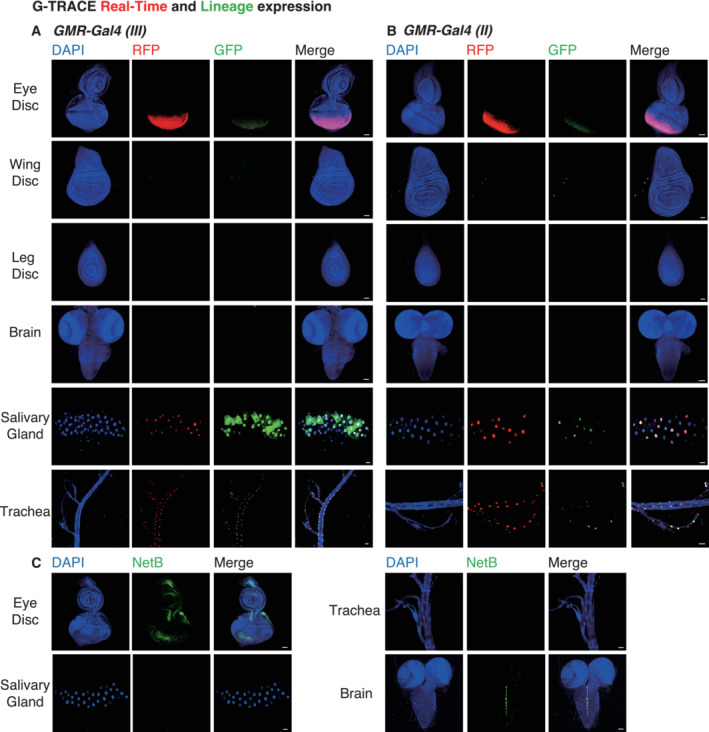
Expression pattern of NetB and *GMR‐Gal4* A, BThe expression pattern of two independent *GMR‐Gal4* lines (second chromosome (A) and third chromosome (B)) in six tissues (eye discs, wing imaginal discs, leg discs, brain, salivary gland, and trachea) using G‐TRACE system. G‐TRACE uses fluorescent protein reporters for real‐time (RFP) and lineage‐based analysis (GFP). Single confocal z‐section images. Scale bar, 50 μm.CThe amount of NetB protein in the eye discs, salivary gland, trachea, and brain of *GMR*‐*Ras* ^*V12*^ flies. CPTI‐000748, a protein trap of NetB, labels endogenous NetB. Single confocal z‐section images. Scale bar, 50 μm. Source data are available online for this figure.

Since the local event is relatively minor without metastasis or extensive overgrowth, we hypothesized that the dysplastic tissue that expresses Ras^V12^ may secrete humoral factors that could mediate systemic effects of the oncogenic stress, leading to organismal death. We performed RNA sequencing using the eye disc tissue from control and *GMR‐Ras* ^*V12*^ flies. Among the genes encoding secreted, we identified 20 secreted proteins that were highly upregulated in the eye disc from *GMR‐Ras* ^*V12*^ flies (Fig 1B). Among these, we found that inhibition of *NetrinB* (*NetB*) reverses organismal lethality induced by Ras^V12^ (Figs 1C and D, and EV1N, O, and P). *NetB* inhibition did not reduce the eye size or the rough eye phenotype in *GMR‐Ras* ^*V12*^ flies (Fig EV1Q–S), suggesting that the local event is intact. Ectopic expression of *NetB* in the eye disc of normal animals induced lethality (Fig 1E). Consistent with the RNA‐seq data, RT–qPCR confirmed that the eye disc of *GMR‐Ras* ^*V12*^ flies upregulates *NetB* mRNA (Fig 1F). The eye disc of *GMR‐Ras* ^*V12*^ flies also has higher levels of NetB protein (Fig 1G–I). Although *GMR‐Gal4* promotes Gal4 expression in the eye disc, trachea, and salivary glands as previously demonstrated (Li *et al*, 2012), only the eye disc expresses detectable levels of NetB, suggesting that NetB in the eye disc could be critical for organismal death (Fig EV2A–C).

### NetB secreted from the eye dysplasia functions in the fat body

**Figure 2:**
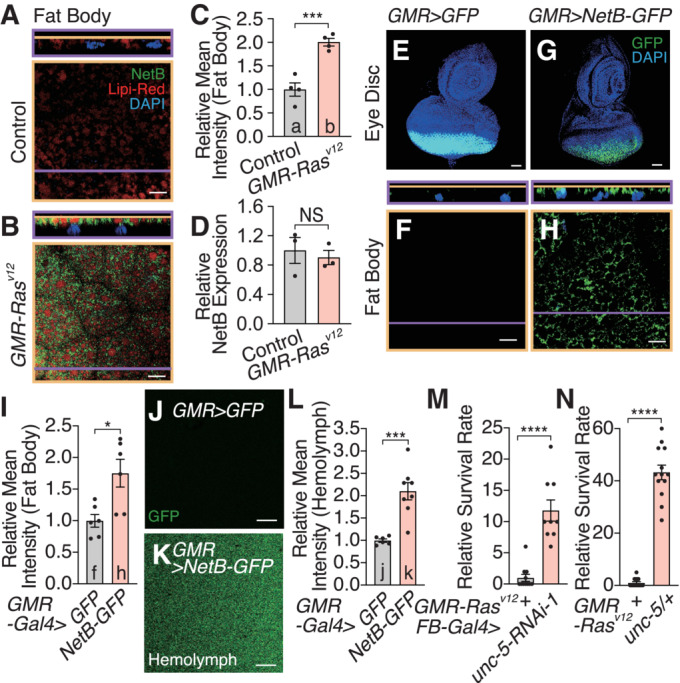
NetB secreted from the eye dysplasia functions in the fat body A, BThe amount of NetB protein increases in the fat body *GMR*‐*Ras* ^*V12*^ (A) compared to control flies (B). CPTI‐000748, a protein trap of NetB, labels endogenous NetB.CQuantification of mean intensity of GFP signals in (A) and (B).DThere is no difference of *NetB* mRNA expression in the fat body of *GMR*‐*Ras* ^*V12*^ and control flies.E–HEctopic expression of GFP‐tagged NetB in the eye disc induces high‐level GFP‐NetB protein in the fat body. Single confocal z‐section are shown in images (E and G).IQuantification of mean intensity of GFP signal in the fat body of (F) and (H).J, KEctopic expression of GFP‐tagged NetB in the eye disc leads to detection of GFP signals in the hemolymph.LQuantification of median intensity of GFP signals in (J) and (K).MKnockdown of *unc‐5*, NetB receptor, in the fat body increases organismal survival over the oncogenic stress.N
*unc‐5* heterozygous mutants flies survive better over Ras^v12^‐induced oncogenic stress. Data information: Data points indicate biological replicates. Data are mean ± s.e.m., and the statistical significance was determined using a two‐tailed unpaired *t*‐test (C, D, I, and L–N). NS, not significant, *P < 0.05, ***P < 0.001, ****P < 0.0001. Scale bar, 20 μm (A and B, F–H, and J and K), 50 μm (E–G). Source data are available online for this figure.

**Figure EV3:**
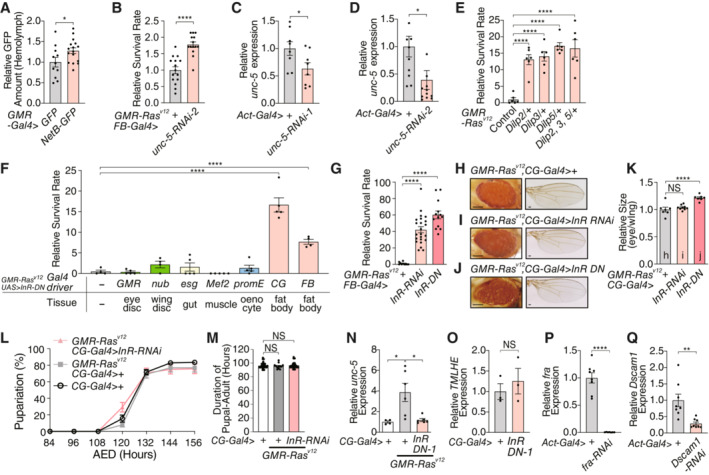
Insulin inhibition increases survival of *GMR‐Ras* ^*V12*^ flies AEctopic expression of GFP‐tagged NetB in the eye disc leads to GFP signals, which were detected by a spectrophotometer, in the hemolymph.BKnockdown of *unc‐5* in the fat body increases survival of *GMR‐Ras* ^*V12*^ flies.C, DqRT–PCR analysis of *unc‐5* RNAi efficiency. *unc‐5* RNAis lowered the expression of *unc‐5* mRNA.E
*Dilp* heterozygous mutants survive better over oncogenic *Ras* expression in the imaginal disc.FExpression of a dominant‐negative form of insulin receptor (InR‐DN) in the fat body (*CG*‐*Gal4*, *FB*‐*Gal4*) but not in other tissues increases survival over oncogenic *Ras* expression in the imaginal disc. We used the following *Gal4* lines: *GMR‐Gal4* (eye disc), *nub*‐*Gal4* (wing disc), *esg*‐Gal4 (gut; intestinal stem cells), *Mef2*‐*Gal4* (somatic muscle), *promE*‐*Gal4* (oenocyte), *CG*‐*Gal4* (fat body), and *FB*‐*Gal4* (fat body).GKnockdown of InR (InR) or expressing a dominant‐negative form of InR (InR‐DN) in the fat body using *FB*‐*Gal4* driver increases survival over oncogenic *Ras* expression in the imaginal disc.H–JInR manipulation in the fat body does not affect the eye disc. Representative images of adult eyes and wings from *GMRRas* ^*V12*^, *CG‐Gal4 > +* (H), *GMR*‐*Ras* ^*V12*^, *CG‐Gal4 > InR‐RNAi* (I), and *GMR*‐*Ras* ^*V12*^, *CG‐Gal4 > InR‐DN* (J). Scale bar, 100 μm.KQuantification of the eye area in (H–J). The adult eye area was measured and normalized against the adult wing area.L, MInR knockdown in the fat body does not induce developmental delay. The time for each larva to reach the pupal stage (L) and the duration of pupal‐adult development for each pupa (M) was determined and plotted. AED, hours after egg deposition.NqRT–PCR analysis of *unc‐5* expression in the fat body. *unc‐5* mRNA was significantly increased in the fat body of *GMR‐Ras* ^*V12*^ flies, and this was reversed by fat body‐specific expression of InR‐DN.OIn the absence of *GMR‐Ras* ^*V12*^, inhibition of insulin receptor in the fat body does not affect *TMLHE* expression in the fat body.P, QqRT–PCR analysis of RNAi efficiency of *fra* (P) and *Dscam1* (Q). Data information: Data points indicate biological replicates. Data are mean ± s.e.m., and the statistical significance was determined by one‐way ANOVA followed by Dunnett's multiple comparisons test (E, G, K, and M), Tukey's multiple comparison test (N), and two‐tailed unpaired *t*‐test (A–D and O–Q). Data points indicate biological replicates. NS, not significant, *P < 0.05, **P < 0.01, ***P < 0.001, ****P < 0.0001. Source data are available online for this figure.

In mammals and flies, Netrin molecules play major roles in neuronal navigation during development of the nervous system (Serafini *et al*, 1996; Kennedy, 2000; Bradford *et al*, 2009). In addition, Netrin and its receptors have been implicated in tumorigenesis in some types of cancers (Arakawa, 2004; Kefeli *et al*, 2017; Hao *et al*, 2020). In general, Netrin molecules have been described to function within the tissue, and their humoral role has not been known. We speculated that, since NetB is secreted, it might work as a humoral factor. To test the hypothesis that NetB from the *GMR‐Ras* ^*V12*^ eye disc reaches remote organs, we examined non‐tumor tissues. We found that NetB protein abundantly exists in the fat body of *GMR‐Ras* ^*V12*^ flies (Fig 2A–C). Importantly, endogenous expression of *NetB* mRNA in the fat body was unchanged in *GMR‐Ras* ^*V12*^ flies compared to control flies (Fig 2D), strongly suggesting that NetB protein observed in the fat body of *GMR‐Ras* ^*V12*^ flies is not due to its increased transcription. To completely exclude a possibility that NetB is generated in the fat body, we made a transgenic line *UAS‐NetB‐GFP* to visualize the incorporation of NetB in the fat body. Ectopic expression of the GFP‐tagged NetB, but not control GFP, in the eye disc led to existence of GFP signals in both the hemolymph and the fat body (Figs 2E–L and EV3A), providing further evidence that NetB secreted by the eye disc humorally relays the signal to the fat body. Furthermore, inhibition of a NetB receptor, Unc‐5 in the fat body increased organismal survival over oncogenic stress (Figs 2M and N, and EV3B–D), suggesting an involvement of NetB signaling in the fat body.

### NetB regulates *TMLHE* expression through Unc‐5 in the fat body of *GMR‐Ras* ^*V12*^ flies

**Figure 3:**
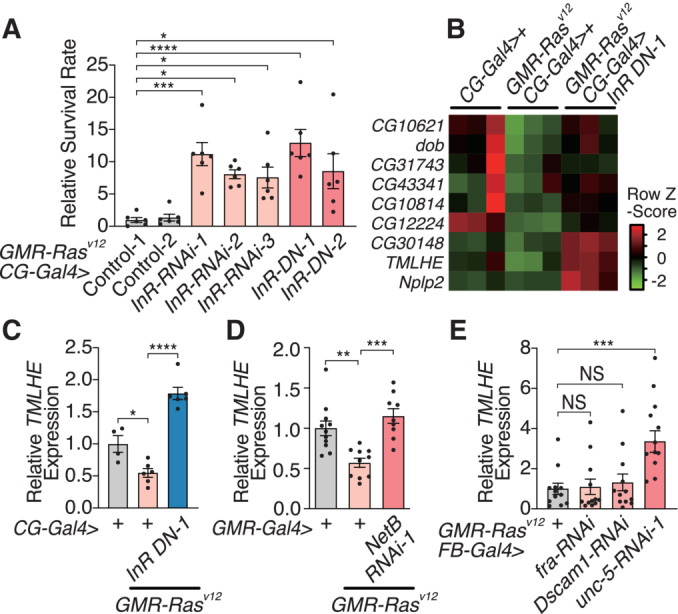
NetB regulates *TMLHE* expression through Unc‐5 in the fat body of *GMR*‐*Ras* ^*V12*^ flies Knockdown of InR (InR) or expressing a dominant‐negative form of InR (InR‐DN) in the fat body using *CG*‐*Gal4* increases survival over oncogenic *Ras* expression in the imaginal disc.Expression of selected gene that were down‐regulated in the fat body of *GMR‐Ras* ^*V12*^ flies and up‐regulated by inhibition of insulin signals (*GMR‐Ras* ^*V12*^, *CG‐Gal4 > InR‐DN*).qRT–PCR demonstrates that *TMLHE* expression in the fat body is reduced by *Ras* expression in the eye disc and reversed by insulin inhibition in the fat body.qRT–PCR demonstrates that *NetB* knockdown in the eye disc increases *TMLHE* expression in the fat body.Knockdown of *unc‐5*, but not other NetB receptors (*Fra* or *Dscam1*), in the fat body increases *TMLHE* expression. Data information: Data points indicate biological replicates. Data are mean ± s.e.m., and the statistical significance was determined by one‐way ANOVA followed by Tukey's multiple comparison test (C and D) and Dunnett's multiple comparisons test (A and E). NS, not significant, *P < 0.05, **P < 0.01, ***P < 0.001, ****P < 0.0001. Source data are available online for this figure.

How does Netrin signaling regulate the fat body and organismal metabolism? To get a clue on Netrin‐mediated signals, we performed RNAseq of the fat body with/without *GMR‐Ras* ^*V12*^ dysplasia. Importantly, because in a different line of research, we had already obtained data that insulin signaling inhibition in the fat body enhances organismal survival of *GMR‐Ras* ^*V12*^ flies, phenocopying the Netrin inhibition, potentially through downregulation of the NetB receptor *unc‐5* (Figs 3A and EV3E–N), we focused on genes that are regulated by Ras and reversed by insulin inhibition. Among such genes is trimethyllysine hydroxylase, epsilon (TMLHE), which regulates the carnitine biosynthesis pathway (Maas *et al*, 2020). Both RNAseq and RT–qPCR demonstrated that *TMLHE* mRNA is decreased in the fat body of *GMR‐Ras* ^*V12*^ flies and increased by InR knockdown in the fat body (Fig 3B and C). InR knockdown in the fat body does not affect *TMLHE* mRNA in the absence of *GMR‐Ras* ^*V12*^ (Fig EV3O), indicating that insulin signaling regulates *TMLHE* in a context of *GMR‐Ras* ^*V12*^. Importantly, *NetB* inhibition in the Ras‐transformed eye disc reversed the Ras‐dependent *TMLHE* downregulation in the fat body (Fig 3D). Further, only *unc‐5* but no other Netrin receptor knockdown affected *TMLHE* expression (Figs 3E and EV3P and Q), suggesting that Unc‐5 mainly mediates the NetB signal in the fat body.

### Carnitine biosynthesis is reduced in the fat body of *GMR‐Ras*_*V12*_ flies

**Figure 4:**
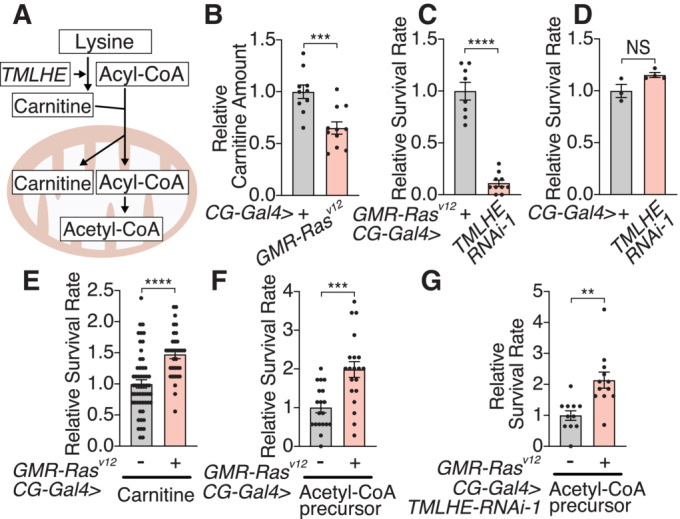
Carnitine biosynthesis is reduced in the fat body of *GMR*‐*Ras* ^*V12*^ flies A schematic of carnitine biosynthesis and its role to transport acyl‐CoA to mitochondria.Local expression of oncogenic *Ras* in the eye disc decreases the amount of carnitine in the fat body.
*TMLHE* knockdown in the fat body aggravates organismal survival over the oncogenic stress.In the absence of *GMR‐Ras* ^*V12*^, inhibition of *TMLHE* in the fat body is not sufficient to induce organismal death.Carnitine feeding increases the survival rate over the oncogenic stress.Acetyl‐CoA precursor (acetate) administration makes *GMR*‐*Ras* ^*V12*^ flies survive.Feeding of acetyl‐CoA precursor (acetate) makes *GMR*‐*Ras* ^*V12*^ flies survive even with *TMLHE* knockdown in the fat body. Data information: Data points indicate biological replicates. Data are mean ± s.e.m., and the statistical significance was determined by two‐tailed unpaired *t*‐test (B–G). NS, not significant, **P < 0.01, ***P < 0.001, ****P < 0.0001. Source data are available online for this figure.

**Figure EV4:**
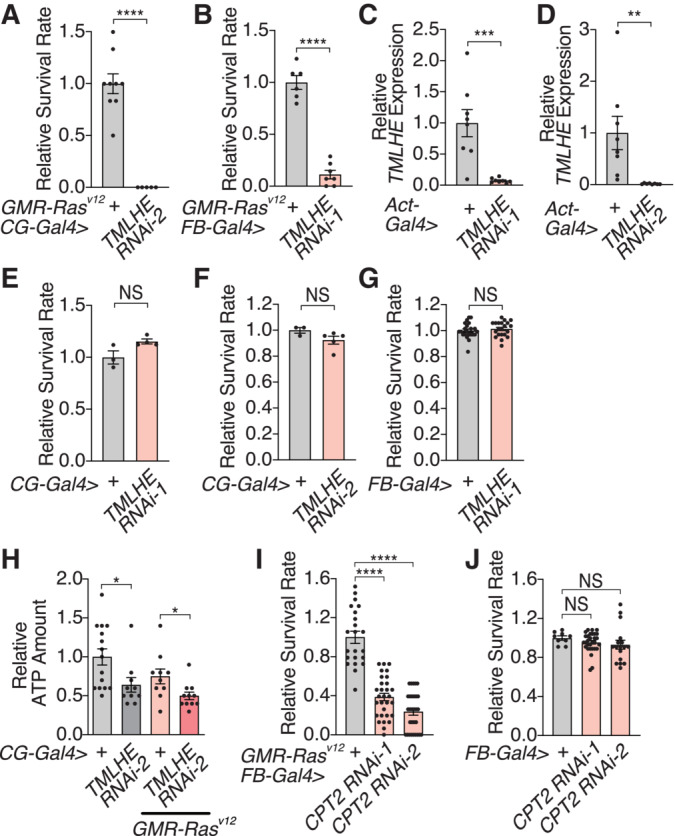
*TMLHE* knockdown in the fat body A, BKnockdown of *TMLHE* in the fat body of *GMR‐Ras* ^*V12*^ flies aggravates organismal survival, demonstrated by using a different RNAi line (A) or another fat body‐specific *FB*‐*Gal4* driver (B).C, DqRT–PCR analysis of *TMLHE* RNAi efficiency. *TMLHE* RNAis lowered expression of *TMLHE* mRNA.E–GIn the absence of tumor burden, inhibition of *TMLHE* in the fat body does not affect organismal death. *TMLHE* was inhibited by *TMLHE* RNAis using *CG*‐*Gla4* (E, F) and *FB*‐*Gla4* driver (G).HInhibition of *TMLHE* in the fat body decreases the amount of ATP in both *GMR‐Ras* ^*V12*^ and control flies.I
*CPT2* knockdown in the fat body aggravates organismal survival over the oncogenic stress.JIn the absence of *GMR‐Ras* ^*V12*^, inhibition of *CPT2* in the fat body is not sufficient to induce organismal death. Data information: Data points indicate biological replicates. Data are mean ± s.e.m., and the statistical significance was determined using a two‐tailed unpaired *t*‐test (A–H) and one‐way ANOVA followed by Dunnett's multiple comparisons test (I and J). NS, not significant, *P < 0.05, **P < 0.01, ***P < 0.001, ****P < 0.0001. Source data are available online for this figure.

TMLHE plays an important role in carnitine biosynthesis and hence acetyl‐CoA production from fatty acids (Fig 4A). Consistently, the amount of carnitine is decreased in the fat body of *GMR‐Ras* ^*V12*^ flies compared to control (Fig 4B). We tested whether manipulation of TMLHE in the fat body could affect organismal death. Knockdown of *TMLHE* in the fat body of *GMR‐Ras* ^*V12*^ flies significantly decreased their survival (Figs 4C and EV4A–D), suggesting that inhibition of *TMLHE* in the fat body makes animals more sensitive to Ras‐transformation. In the absence of *GMR‐Ras* ^*V12*^, inhibition of *TMLHE* in the fat body is not sufficient to induce organismal death (Figs 4D and EV4E–G), suggesting an oncogenic stress‐specific role for TMLHE. Inhibition of *TMLHE* in the fat body decreases the amount of ATP in both *GMR‐Ras* ^*V12*^ and control flies (Fig EV4H). This result indicates *GMR‐Ras* ^*V12*^ flies are more sensitive to ATP reduction induced by *TMLHE* inhibition. Additionally, consistent with the inhibition of *TMLHE*, the fat body‐specific inhibition of carnitine palmitoyltransferase 2 (CPT2), essential for importing fatty acids into mitochondria and catalyzing conversion of acylcarnitine into acyl‐CoA, worsens the organismal death of *GMRRas* ^*V12*^ flies but not control flies (Fig EV4I and J). To further investigate the involvement of carnitine and acetyl‐CoA, a critical metabolite for energy production, in survival of *GMR‐Ras* ^*V12*^ flies, we orally supplemented carnitine or acetyl‐CoA. Since highly charged acetyl‐CoA is a membrane‐impermeant molecule in general, we fed acetate as an acetyl‐CoA precursor (Comerford *et al*, 2014; Pietrocola *et al*, 2015). Carnitine or the acetyl‐CoA precursor administration enhanced survival of *GMR‐Ras* ^*V12*^ flies (Fig 4E and F) and reversed the effect of *TMLHE* knockdown (Fig 4G). Taken together, the Ras‐transformed tissue remotely inhibits carnitine biosynthesis in the fat body, which reduces acetyl‐CoA production, inducing organismal lethality.

### Ras‐induced netrin regulates organismal health in larvae and adults

**Figure 5:**
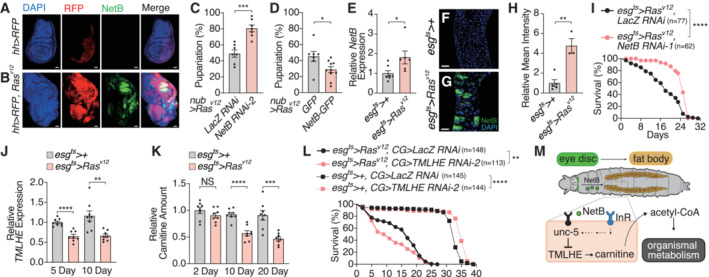
Ras‐induced Netrin regulates organismal health in larvae and adults A, BOncogenic *Ras* ^*V12*^ expression in the wing disc induces high levels of NetB protein. Single confocal z‐section images are shown.CKnockdown of *NetB* in the wing disc of *nub > Ras* ^*V12*^ flies enhances survival.DEctopic expression of *NetB* in the wing disc aggravates organismal survival over the oncogenic stress.EqRT–PCR with mRNA from the adult gut confirms higher expression of *NetB* in *esg* ^*ts*^>*Ras* ^*V12*^ flies after oncogenic *Ras* induction for 10 days.F, GOncogenic *Ras* expression in the adult gut induces NetB protein, which was detected by GFP signal using CPTI‐000748, a protein trap of NetB. *Ras* was induced for 10 days. Single confocal *z*‐section images are shown.HQuantification of GFP signals in (F) and (G).IKnockdown of *NetB* in the adult gut of *esg* ^*ts*^>*Ras* ^*V12*^ flies enhances survival.JLocal expression of oncogenic *Ras* in the adult gut decreases the *TMLHE* mRNA in the fat body. Ras was induced for 5 and 10 days.KLocal expression of oncogenic *Ras* in the adult gut decreases the amount of carnitine. Ras was induced for 2, 10, and 20 days.LKnockdown of *TMLHE* in the fat body of adult flies shortened lifespan of *esg* ^ts^>*Ras* ^*V12*^ flies but not of control flies.MA schematic of the proposed model. Ras^V12^‐transformed tissue‐derived NetB reprograms organismal metabolism through downregulation of carnitine biosynthesis in the fat body. Data information: Data points indicate biological replicates. Data are mean ± s.e.m., and *n* represents the number of flies that were analyzed. The experiments were repeated independently at least twice with similar results (I and L). The statistical significance was determined by two‐tailed unpaired *t*‐test (C–E, H, and J–K) and log‐rank (Mantel‐Cox) test (I and L). NS, not significant, *P < 0.05, **P < 0.01, ***P < 0.001, ****P < 0.0001. Scale bar, 50 μm. Source data are available online for this figure.

**Figure EV5:**
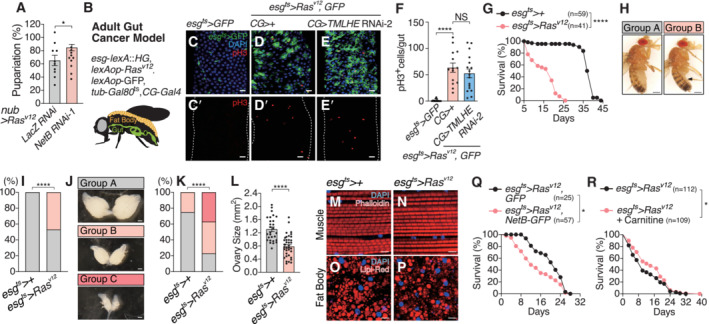
Gut tumor model in adult flies AInhibition of *NetB* in the wing disc reverses *Ras* ^*V12*^‐induced lethality in larvae.BIllustration of the gut tumor model in adult flies. *esg*‐LexA::HG drives Ras^V12^ in intestinal stem cells and *CG*‐*Gal4* drives genes of interest in the fat body. Both *LexA*‐ and *Gal4*‐induced expressions are regulated by a temperature through *Gal80* ^ts^.C–ERepresentative images of *Drosophila* adult stained for DAPI (Nuclei) (C–E) and pH3 (cell proliferation) (C'–E'). Transgenes were induced with *esg* ^ts^ by incubating flies at 30°C for 1 day. Scale bar, 20 μm.FQuantification of the number of pH3‐positive cells per gut in (C–E).GExpression of oncogenic *Ras* in the adult gut using the *esg*‐*LexA* driver shortens lifespan compared to control flies.H, IRepresentative images of flies (H). The arrowheads indicate a shrinked abdomen phenotype. Control and *esg* ^*ts*^>*Ras* ^*V12*^ flies were divided into two classes based on the abdomen phenotypes, as indicated in pictures (I). Oncogenic *Ras* induction for 10 days. Scale bar, 500 μm.J–LRepresentative images of ovaries (J). Control and *esg* ^*ts*^>*Ras* ^*V12*^ flies were divided into three classes based on the ovary's phenotypes, as indicated in (K). Quantification of ovary size (μm^2^) from K (L). Oncogenic *Ras* induction for 10 days. Scale bar, 500 μm.M, NPhalloidin and DAPI staining of dissected thoracic muscle from control (M) and *esg* ^*ts*^>*Ras* ^*V12*^ flies (N). Scale bar, 5 μm.O, PLipi‐Red and DAPI staining of dissected fat body from control (O) and *esg* ^*ts*^>*Ras* ^*V12*^ flies (P). Scale bar, 10 μm.QEctopic expression of *NetB* in the gut induced lethality of *esg* ^*ts*^>*Ras* ^*V12*^ flies.RCarnitine feeding increases the survival rate over the oncogenic stress. Data information: Data points indicate biological replicates. Data are mean ± s.e.m., and *n* represents the number of flies that were analyzed. The experiments were repeated independently at least twice with similar results (G, Q and R). The statistical significance was determined by two‐tailed unpaired *t*‐test (A), one‐way ANOVA followed by Tukey's multiple comparison test (F), log‐rank (Mantel‐Cox) test (G and Q, R), and chi‐square test (I and K). NS, not significant, *P < 0.05, ****P < 0.0001. Source data are available online for this figure.

To further test generality of our findings, we used two additional settings: *Ras* ^*V12*^ expression in the larval wing disc and in adult intestinal stem cells. We found that ectopic expression of *Ras* ^*V12*^ induces NetB in the non‐neuronal wing disc epithelia (Fig 5A and B). Furthermore, inhibition and ectopic expression of *NetB* reverses and exacerbates *Ras* ^*V12*^‐induced lethality in larvae respectively (Figs 5C and D, and EV5A).

Next, we exploited a tumor model in the adult intestinal stem cells (Apidianakis *et al*, 2009; Markstein *et al*, 2014; Tsuda‐Sakurai *et al*, 2020). We generated a dual genetic system that enabled *Ras* ^*V12*^ expression in adult intestinal stem cells by the *esg*‐*LexA*::*HG* driver and gene manipulation in the fat body by the *Cg*‐*Gal4* driver (Fig EV5B). As previously shown (Apidianakis *et al*, 2009; Markstein *et al*, 2014; Tsuda‐Sakurai *et al*, 2020), *Ras* ^*V12*^ expression induces hyperplasia of the gut epithelia, which is detected by the enhanced phospho‐Histone3 (pH3)‐positive cell number (Fig EV5C–F). Flies with the intestinal Ras tumor die much earlier than control (Fig EV5G). *esg* ^*ts*^>*Ras* ^*V12*^ flies display a shrinked abdomen phenotype and smaller ovaries but not an obvious phenotype in muscles or the fat body (Fig EV5H–P). Consistent with the *GMR‐Ras* ^*V12*^ flies, *Ras* ^*V12*^ expression induces higher levels of *NetB* mRNA and protein in the gut (Fig 5E–H). Importantly, similar to the effect of NetB in *GMR‐Ras* ^*V12*^ flies, inhibition of *NetB* reverses the organismal lethality induced by *Ras* ^*V12*^ in adult intestinal stem cells (Fig 5I). Ectopic expression of *NetB* in the gut shortened lethality of *esg* ^*ts*^>*Ras* ^*V12*^ flies (Fig EV5Q). *TMLHE* mRNA and the amount of carnitine are decreased in *esg* ^*ts*^>*Ras* ^*V12*^ flies compared to control (Fig 5J and K). Carnitine administration enhanced survival of *esg* ^*ts*^>*Ras* ^*V12*^ flies (Fig EV5R). Knockdown of *TMLHE* in the adult fat body aggravated survival of *esg* ^*ts*^>*Ras* ^*V12*^ flies without perturbing tumor proliferation (Figs 5L and EV5C–F), suggesting that carnitine generation in the fat body is also important for survival in the adult tumor situation. Taken together, Ras‐induced Netrin regulates organismal health in larvae, pupae and adults, suggesting generality, to a certain degree, of the Ras‐Netrin signaling.

## Discussion

Here we reveal a mechanism by which local oncogenic stress exacerbates organismal health: Ras‐induced dysplastic tissues secrete NetB, which humorally inhibits *TMLHE* expression in the fat body, leading to reduction of carnitine biosynthesis (Fig 5M). Since Netrin molecules could play local roles in tumorigenesis, they are potential therapeutic targets for cancer treatment (Arakawa, 2004; Kefeli *et al*, 2017; Hao *et al*, 2020). Nentrin‐1 protein levels in the plasma are increased in various cancer patients, which has been noted as a cancer marker without functional implications in its systemic role (Ko *et al*, 2014). On the other hand, serum carnitine levels become low in human cancer patients (Silverio *et al*, 2011). Our findings in *Drosophila* imply a possibility that these two, at a glance unrelated symptoms could be mechanistically linked. If our findings are applicable to humans, inhibition of Netrin signaling in cancer patients may kill two birds with one stone, by improving the systemic symptom as well as by suppressing local tumorigenesis.

We demonstrated that Ras induces NeB expression in several tissues, including the eye disc, wing disc and adult intestinal stem cells. This indicates that NetB induction is a relatively general phenomenon that occurs downstream of oncogenic Ras signaling. The precise mechanism by which Ras induces NeB requires further investigation. Additionally, our research revealed that NetB‐Unc‐5 signaling suppresses *TMLHE* transcription. While the role for NetB in cytoskeleton regulation during axon guidance has been well studied (Serafini *et al*, 1996; Kennedy, 2000; Bradford *et al*, 2009), its role in regulation of metabolism‐related gene transcription remains unclear and requires further investigation.

One question is why Ras‐transformed tissues actively secrete NetB to suppress carnitine production in the fat body. Considering that the organismal response to oncogenic stress, especially such a mechanism that exacerbates organismal health, likely has not been evolutionarily selected, we speculate that humorally mediated NetB signaling in the fat body may play an alternative, more adaptive role in a more physiological context, which Ras‐transformed tissues hijack accidently. We hypothesize that NetB signaling might have evolved to couple and coordinate two events simultaneously: local neuronal pathfinding and systemic metabolism, both of which oncogenic tissues could take advantage of.

## Materials and Methods

### Fly stocks

Fly stocks used in this study are shown in Dataset EV1. Appropriate control RNAi or stocks that express control molecules from a similar genetic background were used as controls. In some experiments, matching *Attp2/Attp40* and OregonR were used as control for TRiP RNAi lines and VDRC lines, respectively. We confirmed that UAS‐control RNAi expression does not affect the survival rate compared to OregonR and w‐. Genotypes used in each figure are shown in Dataset EV3.

### *Drosophila* husbandry and feeding assay

Flies were maintained as previously described (Yoo *et al*, 2016). The fly food is composed of the following ingredients: 0.8% agar, 10% glucose, 4.5% corn flour, 3.72% dry yeast, 0.4% propionic acid, 0.3% butyl p‐hydroxybenzoate. For acetate supplementation experiments, acetate (FUJIFILM Wako) was added to the fly food to a final concentration of 333 or 500 mM. Carnitine (Tokyo Chemical Industry) was also added to the fly food to a final concentration of 100 mM.

### Plasmid construction and transgenesis

The cDNA encoding NetB was amplified with Phusion High‐Fidelity DNA polymerase (Thermo Fisher Scientific) and subcloned into the pEGFP‐N1vector (Addgene) by KpnI/EcoRI. Then, NetB‐EGFP fragment was subcloned into the pJFRC7‐20XUAS‐IVS‐mCD8‐GFP vector (Addgene) by XhoI/Xba. The plasmid inserted into the attP2 site using phiC31‐mediated transgenesis (Best Gene).

### Measurement of the survival rate and developmental timing

Measurement of the survival rate was performed as previously (Nishida *et al*, 2021). After mated females were allowed to lay eggs on grape agar plates for 24 h at 25°C, L1 stage larvae were collected from grape agar plates and placed into treatment vials with different food conditions (50 larvae/vial). The number of adult flies of each genotype that could enclose was recorded. Survival rates were calculated as the number of adult flies that eclosed divided by the expected number of larvae of each genotype placed in each vial. For developmental timing assay, the number of larvae that had pupariated was recorded at the indicated time points after egg deposition (AED). Most experiments were performed at 25°C, except the ones performed to increase the sensitivity of the assays at 23°C in Figs 1C, 3C, and 4B or 30°C in Figs 1E, EV1O and P, EV3C, D, P, and Q, 5E, F, G, H, I, J, K, and L, and EV5B, C, D, E, F, G, H, I, J, K, L, M, N, O, P, Q, and R

### Quantification of the size of eye, wing, and ovary

Bright view photographs were taken by using a digital CCD color camera (Nikon Digital Sight DS‐Fi2) attached to a Nikon SMZ18 stereomicroscope (Nikon Instruments Inc.). The eye, wing, or ovary areas were manually traced and measured using ImageJ software (National Institutes of Health).

### RNA sequencing

Total RNA was isolated from the fat body and the eye disc from L3 stage larvae with indicated genotypes using the RNeasy Mini Kit (Qiagen). Library construction and sequencing for the eye disc were carried out by Macrogen Japan Corp. RNA‐seq libraries for the fat bodies were prepared using the TruSeq Stranded mRNA Sample Prep Kit (Illumina). The prepared libraries were sequenced by the HiSeq 1500. The obtained reads were mapped and analyzed by CLC Genomics Workbench version 20.0.4 software (Filgen). The expression heat maps were drawn using the online program Heatmapper (Babicki *et al*, 2016). Data have been deposited in the DDBJ Sequence Read Archive (DRA) (accession number: DRA015648 and DRA015649).

### Quantitative RT–PCR (qRT–PCR)

Quantitative RT–PCRs were performed as described (Okada & Shi, 2018). Briefly, Total RNA was isolated from the fat body, eye disc, and whole body from L3 stage larvae using the Maxwell RSC simplyRNA Tissue Kit (Promega). The reverse transcription (RT) reaction was carried out using the ReverTra Ace qPCR RT Kit (Toyobo). The resulting cDNA was diluted 1:5, and the diluted products (2 μl) were subjected to PCR by using a FastStart Essential DNA Green Master Mix (Roche) in a 10 μl of reaction solution and the LightCycler 96 (Roche) according to the manufacturer's protocol. The level of mRNAs was normalized against the level of RpL32 mRNA for each sample. Primers used for qRT–PCRs are shown in Dataset EV2.

### Immunofluorescence and imaging

For immunostaining, the eye imaginal disc, the fat body, muscle, adult midguts, and adult ovaries were dissected in PBS, fixed with paraformaldehyde in PBS, and washed in PBS with 0.1% Triton X‐100, according to the method described previously (Sasaki *et al*, 2021). The following reagents were used at indicated dilution: DAPI (1:500; D9542, Sigma), rabbit‐anti‐phospho‐H3 (1:200; 06‐570, Merck), Alexa rabbit Fluor 568 secondary antibody (1:500; A‐11036, Thermo Fisher Scientific), Alexa Fluor 568 phalloidin (1:500; A‐12380, Thermo Fisher Scientific), and GFP‐Booster Alexa Fluor^®^ 488 (1:200; gb2AF488‐50, ChromoTek). For lipid droplet staining, fat bodies were stained with Lipi‐Red (1:1,000; LD03, Dojindo Molecular Technologies). Fluorescence images were acquired with a confocal microscope (Zeiss LSM 880, 900) as previously described (Ciesielski *et al*, 2022). Quantification of the intensity measurement of fluorescent signals was performed by ImageJ software (National Institutes of Health) or IMARIS 9.5.1 (Bitplane).

### Measurement of carnitine in the fat body

Measurement of carnitine in the fat body were performed by using ultra‐high‐performance liquid chromatography with tandem mass spectrometry (UPLC‐MS/MS). The fat bodies from two larvae at the L3 stage were used per sample and samples were processed according to previous description (Nishida *et al*, 2021). The detection was carried out on a XEVO TQ‐S triple quadrupole tandem mass spectrometer coupled with electrospray ionization source (Waters). Precursor ion was scanned at m/z (MH^+^: 162.073 > 102.825 for Carnitine) by multiple reaction monitoring and established methods using individual authentic compounds and biological samples. The peak area of a target metabolite was analyzed using MassLynx 4.1 software (Waters). The insoluble pellets were heat‐denatured with 0.2 N NaOH and used to quantify total protein using a BCA protein assay kit (Thermo Fisher Scientific).

### Measurement of GFP signals in the hemolymph

GFP signals in the hemolymph were measured by either microscopy or spectrophotometry. For GFP signal measurement using a microscope, the hemolymph from 10 larvae at the L3 stage was collected and spread onto a glass slide. Then, fluorescent images of the hemolymph were acquired using Zeiss LSM 900 confocal microscope. For GFP signal measurement using a Nanodrop spectrophotometer (Thermo Fisher Scientific), the hemolymph from three larvae at the L3 stage was collected and then measured for the absorbance at 509 nm. The standard curve was generated for each trial.

### ATP measurement

ATP measurement was performed as previously described (Figueroa‐Clarevega & Bilder, 2015). Briefly, the fat body from L3 stage larvae was homogenized in 80 μl of extraction buffer (6 M guanidine hydrochloride, 4 mM EDTA, 100 mM Tris–HCl, pH 8.0), boiled at 100°C for 5 min, and centrifuged at 4°C for 15 min. ATP levels were quantified using an ATP Determination Kit (A‐22066, Thermo Fisher Scientific).

### Measurement of triglycerides and trehalose

Triglycerides and trehalose were measured as described previously (Matsuda *et al*, 2015). In brief, L3 larvae were homogenized in PBS with 0.1% Triton X‐100, heated to 80°C for 10 min, and then cooled to room temperature. For the measurement of whole‐body triglycerides, 5 μl of the homogenate was mixed with 5 μl of a triglyceride reagent (T2449, Sigma‐Aldrich) and incubated at 37°C for more than 30 min. Ten microliters of the mixture was used for triacylglycerol (TAG) determination with free glycerol reagent (F6428, Sigma‐Aldrich). The amount of free glycerol was subtracted from the measurements. For measurement of whole‐body trehalose, 5 μl of supernatant was mixed with 0.3 μl trehalase (T8778, Sigma‐Aldrich), and 10 μl buffer (5 mM Tris–HCl (pH 6.6), 137 mM NaCl, and 2.7 mM KCl) at overnight at 37 °C. The amount of glucose was measured using a glucose assay kit (GAGO20, Sigma‐Aldrich). The trehalose concentration was determined for each sample by subtracting the amount of free glucose from the measurements. Protein levels were determined with the Pierce™ BCA Protein Assay Kit (23227, Thermo Fisher Scientific) and used for normalization.

### Statistical analysis

All the statistical analyses were performed using GraphPad Prism 8. Data are presented as mean ± s.e.m. A two‐tailed unpaired *t*‐test was used to test between two samples. One‐way ANOVA with multiple comparison tests was used to compare among group. Log rank (Mantel‐Cox) test was used for comparison of survival distributions. Statistical significance is shown by asterisk; *P < 0.05, **P < 0.01, ***P < 0.001, ****P < 0.0001.

## Author contributions

**Morihiro Okada:** Conceptualization; data curation; formal analysis; funding acquisition; investigation; methodology; writing – original draft; writing – review and editing. **Tomomi Takano:** Investigation; methodology; writing – review and editing. **Yuko Ikegawa:** Investigation; methodology; writing – review and editing. **Hanna Ciesielski:** Investigation; methodology; writing – review and editing. **Hiroshi Nishida:** Investigation; methodology; writing – review and editing. **Sa Kan Yoo:** Conceptualization; supervision; funding acquisition; investigation; methodology; writing – original draft; project administration; writing – review and editing.

## Disclosure and competing interests statement

The authors declare that they have no conflict of interest.

## Supporting information



Expanded View Figures PDF

Dataset EV1

Dataset EV2

Dataset EV3

Source Data for Expanded View

PDF+

Source Data for Figure 1

Source Data for Figure 2

Source Data for Figure 3

Source Data for Figure 4

Source Data for Figure 5

## Data Availability

All raw sequencing reads generated for this study have been deposited to the DNA Data Bank of Japan (DDBJ) database. DRA015648 (https://ddbj.nig.ac.jp/resource/sra‐submission/DRA015648). DRA015649 (https://ddbj.nig.ac.jp/resource/sra‐submission/DRA015649).
