# Supplementary material for: Oncogenic stress‐induced Netrin is a humoral signaling molecule that reprograms systemic metabolism in Drosophila
Source: EMBO J. 2023 May 4;42(12):e111383. doi: 10.15252/embj.2022111383 (PMC10267689; doi:10.15252/embj.2022111383)
Supplement: Supplementary file 1 — Expanded View Figures PDF [file EMBJ-42-e111383-s004.pdf]

Expanded View Figures

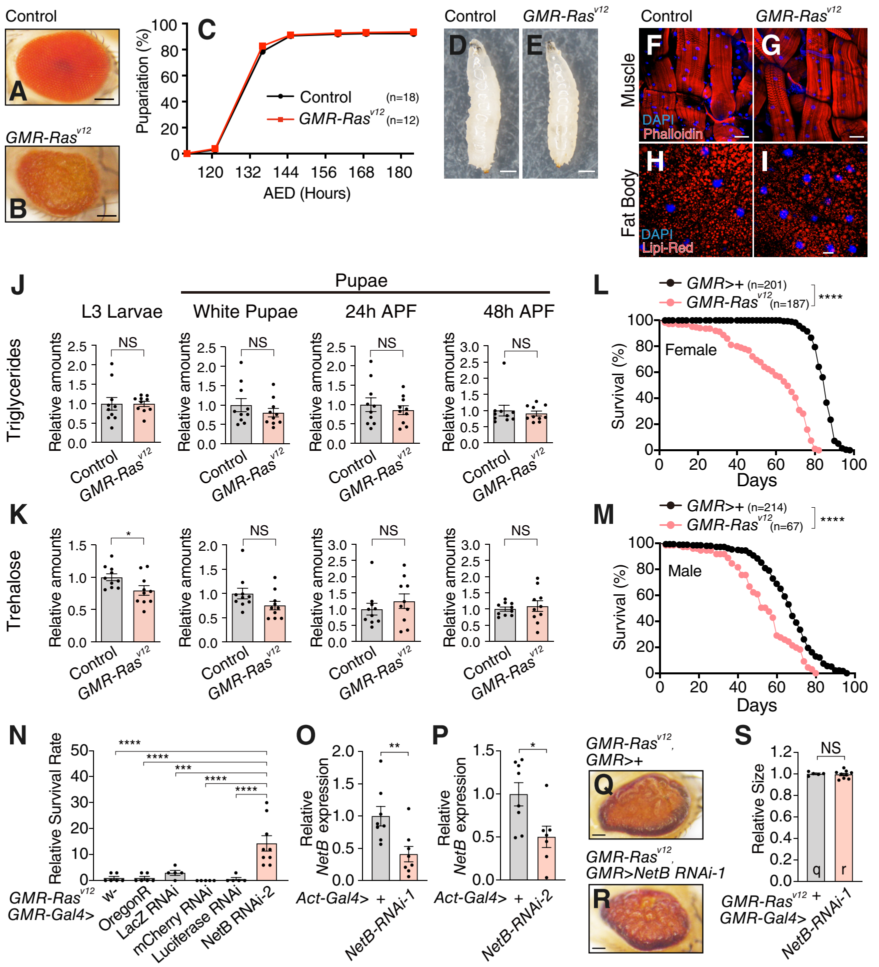

Figure EV1. ***GMR-Ras^{V12}* model and *NetB* expression.**

A, B Representative images of adult eyes from Control (*OregonR*) (A) and *GMR-Ras^{V12}* flies (B).

C No developmental retardation for the timing of pupariation was observed in *GMR-Ras^{V12}* flies. The time for each larva to reach a pupal stage were determined and plotted. AED, hours after egg deposition.

D, E Representative images of control (D) and *GMR-Ras^{V12}* third-instar larvae (E). Scale bar, 500 µm.

F, G Phalloidin and DAPI staining of dissected larval body-wall muscle from control (F) and *GMR-Ras^{V12}* third-instar larvae (G). Single confocal *z*-section images. Scale bar, 50 µm.

H, I Lipi-Red and DAPI staining of dissected fat body from control (H) and *GMR-Ras^{V12}* third-instar larvae (I). Single confocal *z*-section images. Scale bar, 20 µm. Note that no *GMR-Ras^{V12}* third-instar larvae show the bloating symptom or degeneration of muscles/fat.

J, K The amounts of triglycerides (J) and trehalose (K) with the indicated genotypes during development. APF, after puparium formation.

L, M Significant decrease in *GMR-Ras^{V12}* adult female (L) and male (M) lifespan compared to control flies (*GMR* > +).

N UAS-RNAi expression does not affect the survival rate compared to w- and OregonR control.

O, P qRT–PCR analysis of *NetB* RNAi efficiency. NetB RNAis lowered expression of *NetB* mRNA.

Q, R Representative images of adult eyes from *GMR-Ras^{V12}* flies with (R) or without knockdown (Q) of *NetB* in the eye disc using *GMR-Gal4*. Scale bar, 100 µm.

S Quantification of the eye area in (Q) and (R).

Data information: Data points indicate biological replicates. Data are mean ± s.e.m., and *n* represents the number of vials (C) of flies (L, M) that were analyzed. The experiments were repeated independently at least twice with similar results (A and B). The statistical significance was determined by one-way ANOVA followed by Dunnett's multiple comparisons test (N), two-tailed unpaired *t*-test (J and K, O and P, and S), and log-rank (Mantel-Cox) test (L and M). NS, not significant, **P* < 0.05, *****P* < 0.0001.

Source data are available online for this figure.

© 2023 The Authors

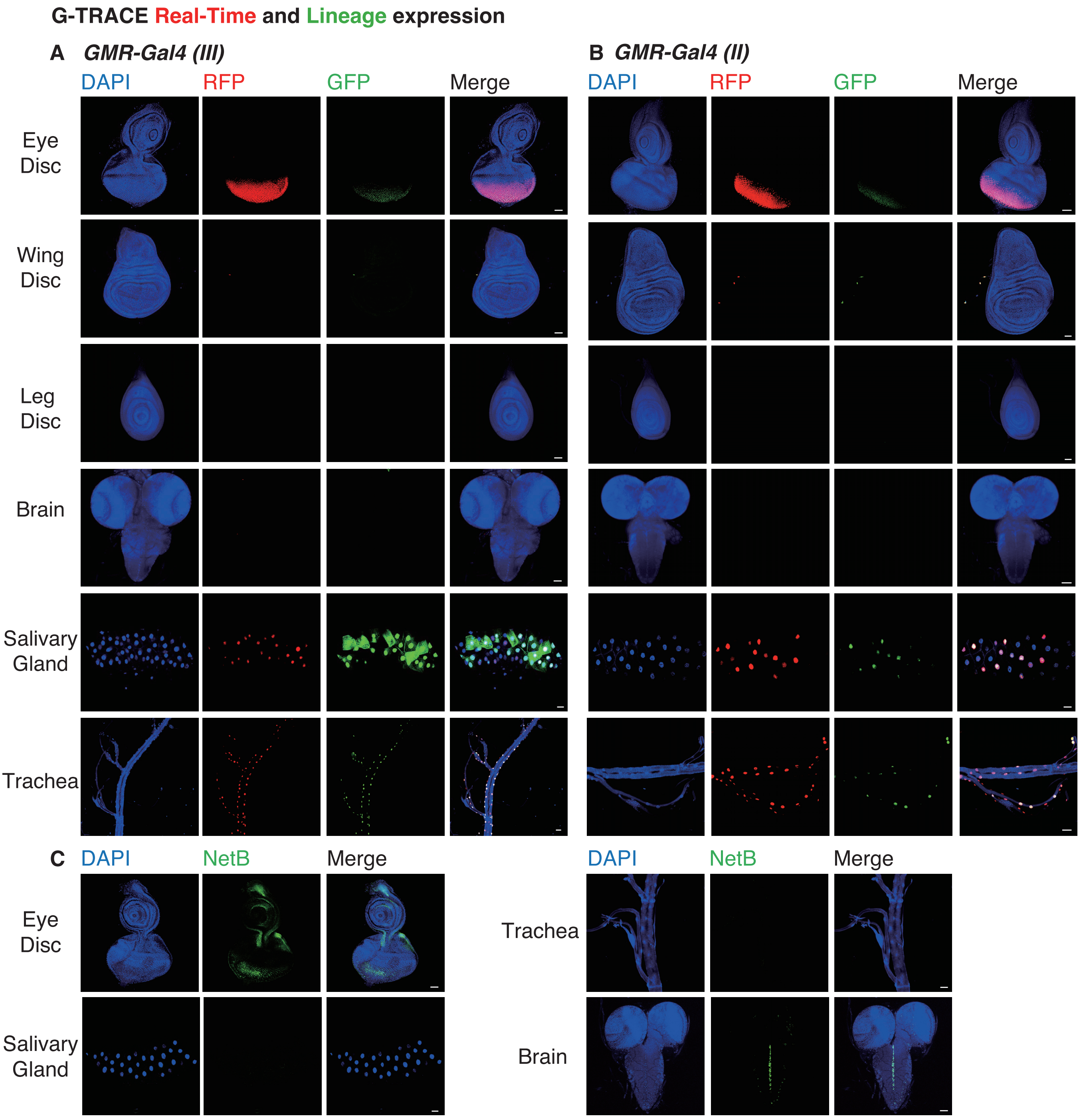

Figure EV2. Expression pattern of NetB and *GMR-Gal4*.

A, B The expression pattern of two independent *GMR-Gal4* lines (second chromosome (A) and third chromosome (B)) in six tissues (eye discs, wing imaginal discs, leg discs, brain, salivary gland, and trachea) using G-TRACE system. G-TRACE uses fluorescent protein reporters for real-time (RFP) and lineage-based analysis (GFP). Single confocal z-section images. Scale bar, 50 μm.

C The amount of NetB protein in the eye discs, salivary gland, trachea, and brain of *GMR-Ras*^{V12} flies. CPTI-000748, a protein trap of NetB, labels endogenous NetB. Single confocal z-section images. Scale bar, 50 μm.

Source data are available online for this figure.

 © 2023 The Authors

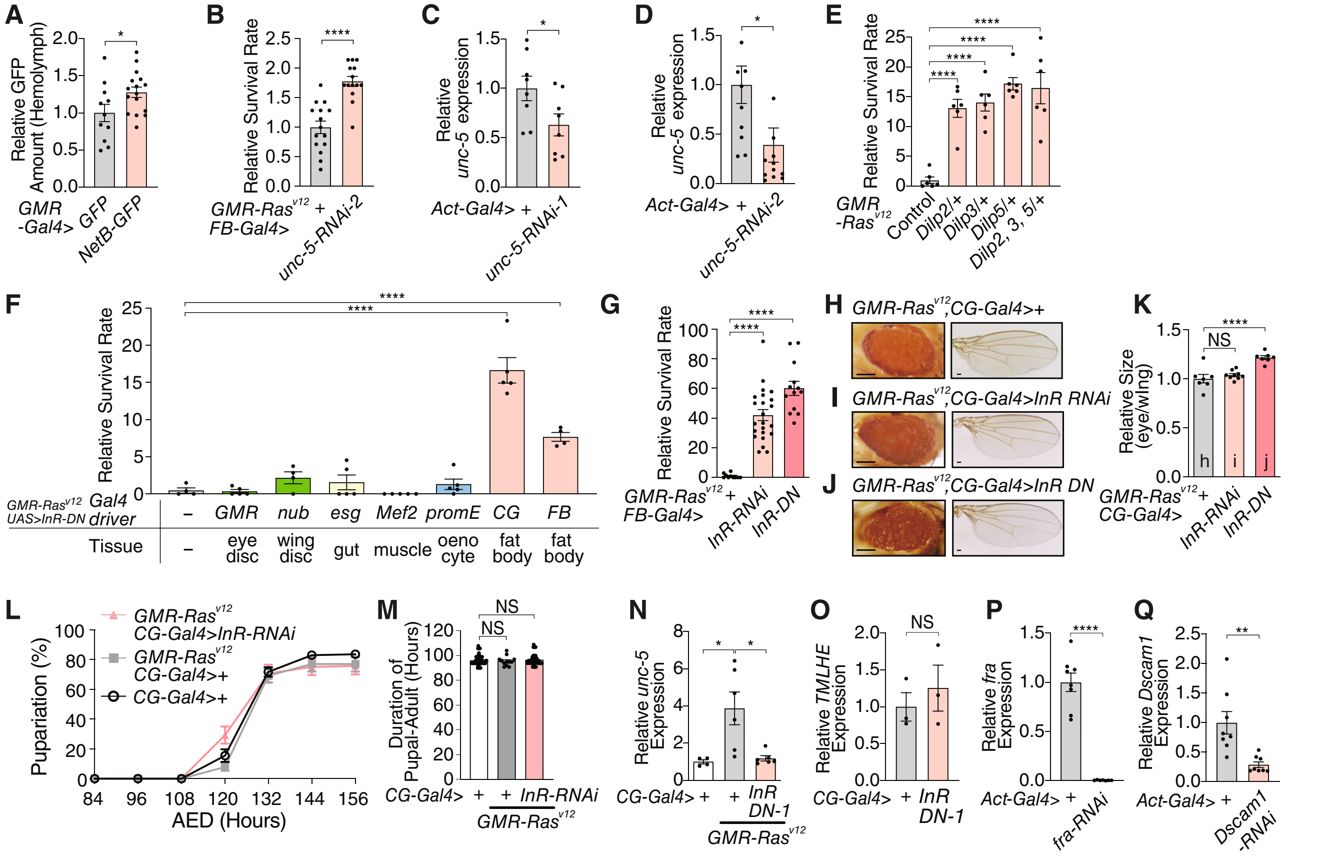

Figure EV3. Insulin inhibition increases survival of *GMR-Ras*^{V12} flies.

- A Ectopic expression of GFP-tagged NetB in the eye disc leads to GFP signals, which were detected by a spectrophotometer, in the hemolymph.
- B Knockdown of *unc-5* in the fat body increases survival of *GMR-Ras*^{V12} flies.
- C, D qRT–PCR analysis of *unc-5* RNAi efficiency. *unc-5* RNAis lowered the expression of *unc-5* mRNA.
- E *Dilp* heterozygous mutants survive better over oncogenic *Ras* expression in the imaginal disc.
- F Expression of a dominant-negative form of insulin receptor (InR-DN) in the fat body (*CG-Gal4*, *FB-Gal4*) but not in other tissues increases survival over oncogenic *Ras* expression in the imaginal disc. We used the following *Gal4* lines: *GMR-Gal4* (eye disc), *nub-Gal4* (wing disc), *esg*-Gal4 (gut; intestinal stem cells), *Mef2-Gal4* (somatic muscle), *promE-Gal4* (oenocyte), *CG-Gal4* (fat body), and *FB-Gal4* (fat body).
- G Knockdown of InR (InR) or expressing a dominant-negative form of InR (InR-DN) in the fat body using *FB-Gal4* driver increases survival over oncogenic *Ras* expression in the imaginal disc.
- H–J InR manipulation in the fat body does not affect the eye disc. Representative images of adult eyes and wings from *GMRRas*^{V12}, *CG-Gal4* > + (H), *GMR-Ras*^{V12}, *CG-Gal4* > *InR-RNAi* (I), and *GMR-Ras*^{V12}, *CG-Gal4* > *InR-DN* (J). Scale bar, 100 μm.
- K Quantification of the eye area in (H–J). The adult eye area was measured and normalized against the adult wing area.
- L, M InR knockdown in the fat body does not induce developmental delay. The time for each larva to reach the pupal stage (L) and the duration of pupal-adult development for each pupa (M) was determined and plotted. AED, hours after egg deposition.
- N qRT–PCR analysis of *unc-5* expression in the fat body. *unc-5* mRNA was significantly increased in the fat body of *GMR-Ras*^{V12} flies, and this was reversed by fat body-specific expression of InR-DN.
- O In the absence of *GMR-Ras*^{V12}, inhibition of insulin receptor in the fat body does not affect *TMLHE* expression in the fat body.
- P, Q qRT–PCR analysis of RNAi efficiency of *fra* (P) and *Dscam1* (Q).

Data information: Data points indicate biological replicates. Data are mean ± s.e.m., and the statistical significance was determined by one-way ANOVA followed by Dunnett's multiple comparisons test (E, G, K, and M), Tukey's multiple comparison test (N), and two-tailed unpaired *t*-test (A–D and O–Q). Data points indicate biological replicates. NS, not significant, $*P < 0.05$, $**P < 0.01$, $***P < 0.001$, $****P < 0.0001$.
Source data are available online for this figure.

© 2023 The Authors

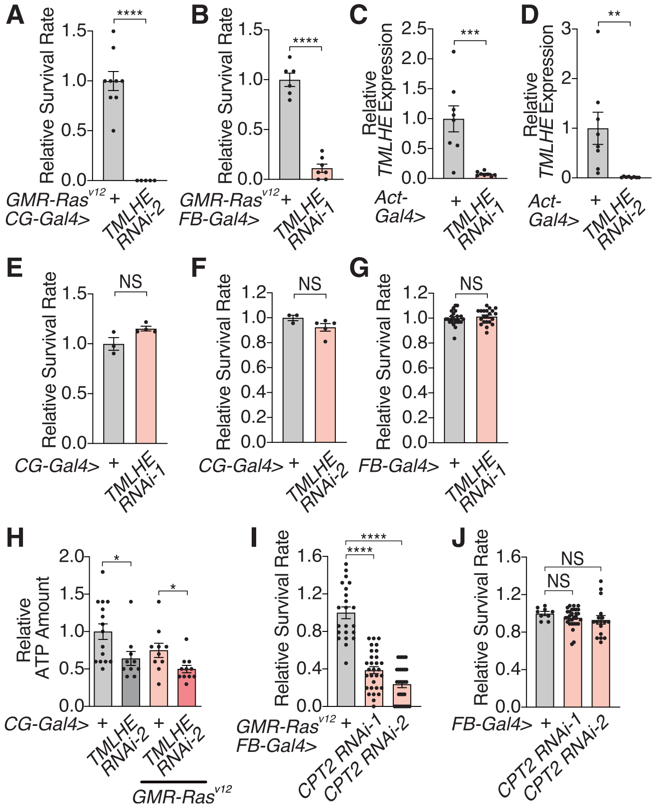

Figure EV4. *TMLHE* knockdown in the fat body.

A, B Knockdown of *TMLHE* in the fat body of *GMR-Ras^{V12}* flies aggravates organismal survival, demonstrated by using a different RNAi line (A) or another fat body-specific *FB-Gal4* driver (B).

C, D qRT–PCR analysis of *TMLHE* RNAi efficiency. *TMLHE* RNAis lowered expression of *TMLHE* mRNA.

E–G In the absence of tumor burden, inhibition of *TMLHE* in the fat body does not affect organismal death. *TMLHE* was inhibited by *TMLHE* RNAis using *CG-Gla4* (E, F) and *FB-Gla4* driver (G).

H Inhibition of *TMLHE* in the fat body decreases the amount of ATP in both *GMR-Ras^{V12}* and control flies.

I *CPT2* knockdown in the fat body aggravates organismal survival over the oncogenic stress.

J In the absence of *GMR-Ras^{V12}*, inhibition of *CPT2* in the fat body is not sufficient to induce organismal death.

Data information: Data points indicate biological replicates. Data are mean ± s.e.m., and the statistical significance was determined using a two-tailed unpaired *t*-test (A–H) and one-way ANOVA followed by Dunnett's multiple comparisons test (I and J). NS, not significant, $^*P < 0.05$, $^{**}P < 0.01$, $^{***}P < 0.001$, $^{****}P < 0.0001$.

Source data are available online for this figure.

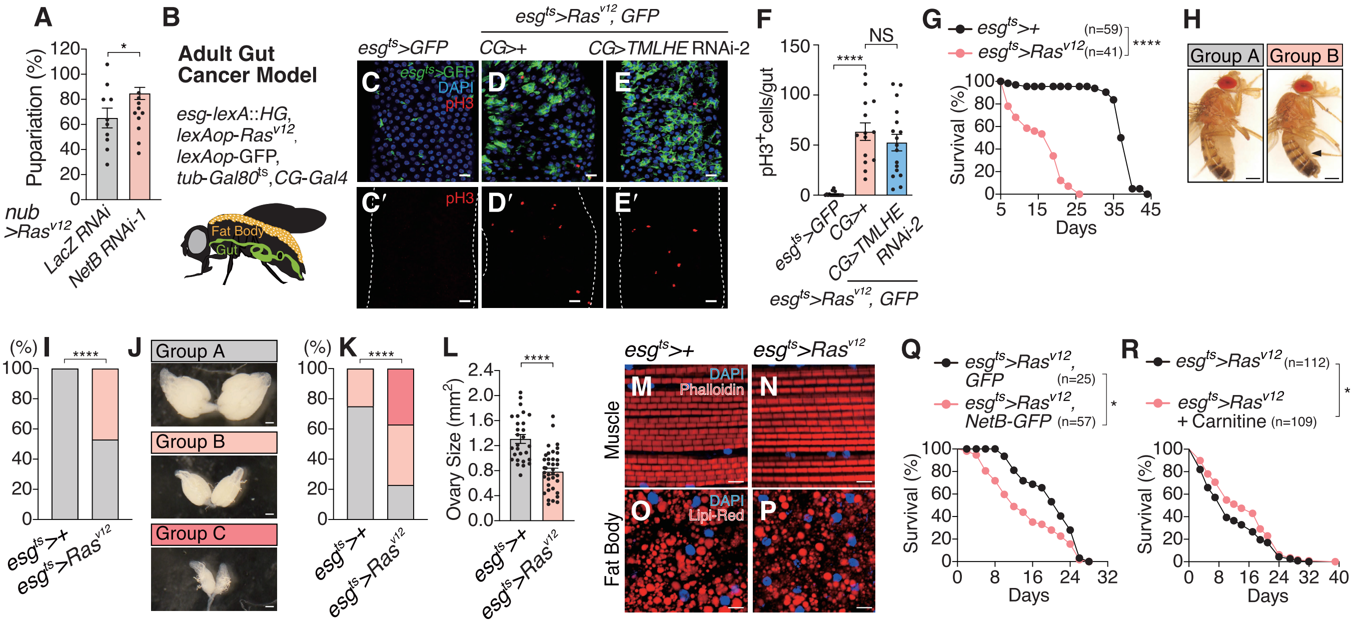

Figure EV5.

© 2023 The Authors

◀ **Figure EV5. Gut tumor model in adult flies.**

A Inhibition of *NetB* in the wing disc reverses *Ras*^{V12}-induced lethality in larvae.

B Illustration of the gut tumor model in adult flies. *esg*-LexA::HG drives Ras^{V12} in intestinal stem cells and *CG-Gal4* drives genes of interest in the fat body. Both *LexA*- and *Gal4*-induced expressions are regulated by a temperature through *Gal80*^{ts}.

C–E Representative images of *Drosophila* adult stained for DAPI (Nuclei) (C–E) and pH3 (cell proliferation) (C'–E'). Transgenes were induced with *esg*^{ts} by incubating flies at 30°C for 1 day. Scale bar, 20 μm.

F Quantification of the number of pH3-positive cells per gut in (C–E).

G Expression of oncogenic *Ras* in the adult gut using the *esg-LexA* driver shortens lifespan compared to control flies.

H, I Representative images of flies (H). The arrowheads indicate a shrinked abdomen phenotype. Control and *esg*^{ts}>*Ras*^{V12} flies were divided into two classes based on the abdomen phenotypes, as indicated in pictures (I). Oncogenic *Ras* induction for 10 days. Scale bar, 500 μm.

J–L Representative images of ovaries (J). Control and *esg* ^{ts}>*Ras*^{V12} flies were divided into three classes based on the ovary's phenotypes, as indicated in (K). Quantification of ovary size (μm^2) from K (L). Oncogenic *Ras* induction for 10 days. Scale bar, 500 μm.

M, N Phalloidin and DAPI staining of dissected thoracic muscle from control (M) and *esg*^{ts}>*Ras*^{V12} flies (N). Scale bar, 5 μm.

O, P Lipi-Red and DAPI staining of dissected fat body from control (O) and *esg*^{ts}>*Ras*^{V12} flies (P). Scale bar, 10 μm.

Q Ectopic expression of *NetB* in the gut induced lethality of *esg*^{ts}>*Ras*^{V12} flies.

R Carnitine feeding increases the survival rate over the oncogenic stress.

Data information: Data points indicate biological replicates. Data are mean ± s.e.m., and *n* represents the number of flies that were analyzed. The experiments were repeated independently at least twice with similar results (G, Q and R). The statistical significance was determined by two-tailed unpaired *t*-test (A), one-way ANOVA followed by Tukey's multiple comparison test (F), log-rank (Mantel-Cox) test (G and Q, R), and chi-square test (I and K). NS, not significant, *$P < 0.05$, ****$P < 0.0001$. Source data are available online for this figure.
