# Supplementary figures and images for: Oncogenic stress‐induced Netrin is a humoral signaling molecule that reprograms systemic metabolism in Drosophila
Source: EMBO J. 2023 May 4;42(12):e111383. doi: 10.15252/embj.2022111383 (PMC10267689; doi:10.15252/embj.2022111383)

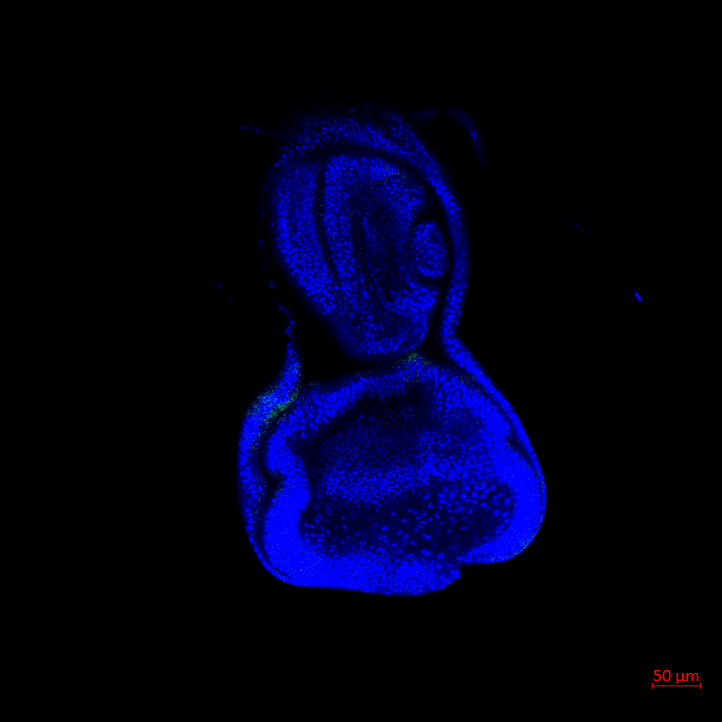

Supplement: Supplementary file 7 — Source Data for Figure 1 [file EMBJ-42-e111383-s012.zip › Source Data for Figure 1/Fig. 1g/Fig. 1g_Merge.tif]

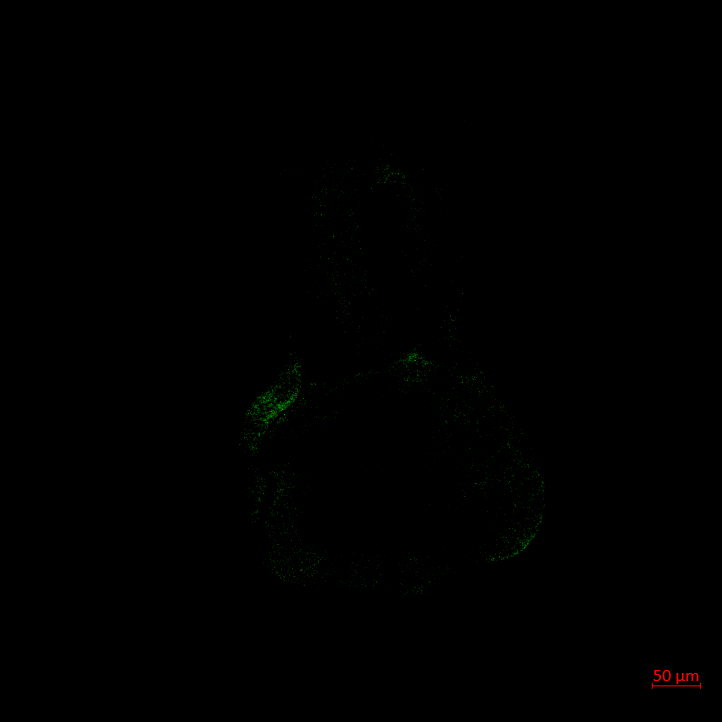

Supplement: Supplementary file 7 — Source Data for Figure 1 [file EMBJ-42-e111383-s012.zip › Source Data for Figure 1/Fig. 1g/Fig. 1g_NetB.tif]

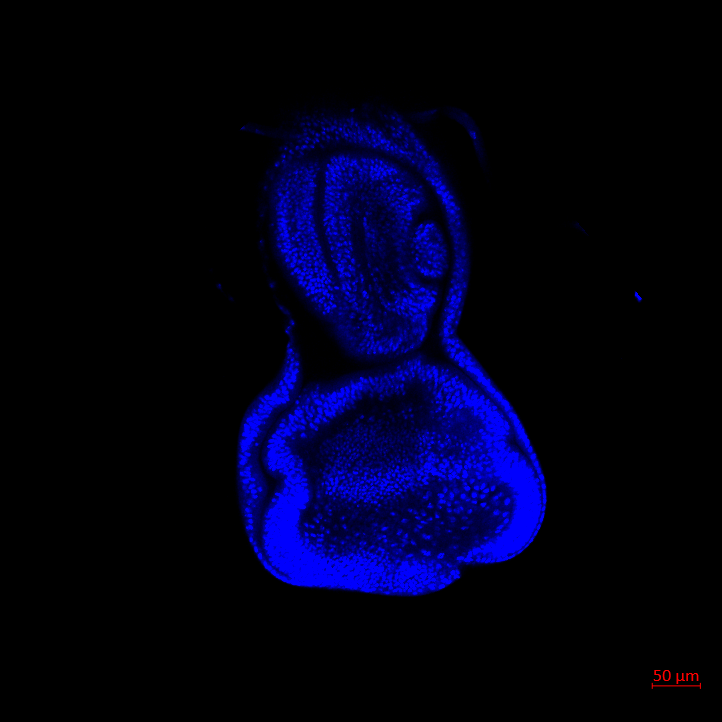

Supplement: Supplementary file 7 — Source Data for Figure 1 [file EMBJ-42-e111383-s012.zip › Source Data for Figure 1/Fig. 1g/Fig. 1g_DAPI.tif]

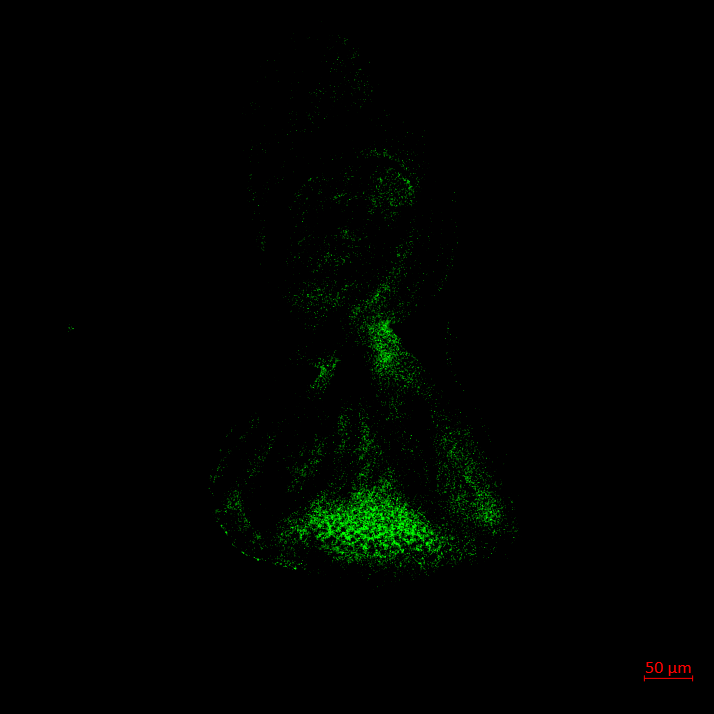

Supplement: Supplementary file 7 — Source Data for Figure 1 [file EMBJ-42-e111383-s012.zip › Source Data for Figure 1/Fig. 1h/Fig. 1h_NetB.tif]

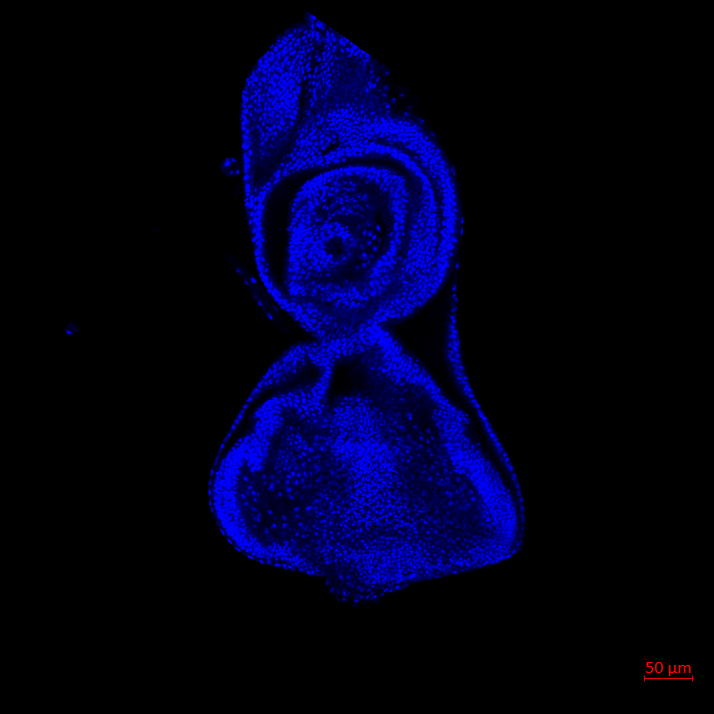

Supplement: Supplementary file 7 — Source Data for Figure 1 [file EMBJ-42-e111383-s012.zip › Source Data for Figure 1/Fig. 1h/Fig. 1h_DAPI.tif]

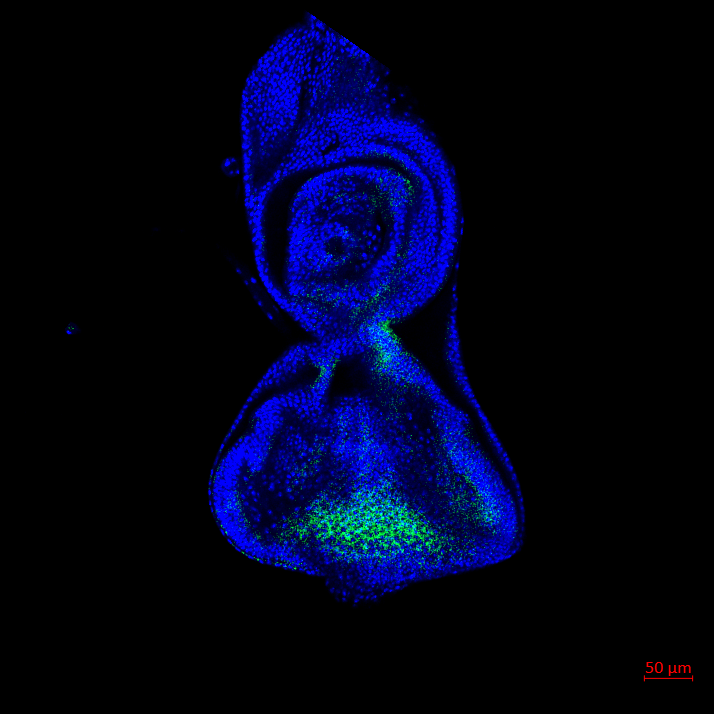

Supplement: Supplementary file 7 — Source Data for Figure 1 [file EMBJ-42-e111383-s012.zip › Source Data for Figure 1/Fig. 1h/Fig. 1h_Merge.tif]

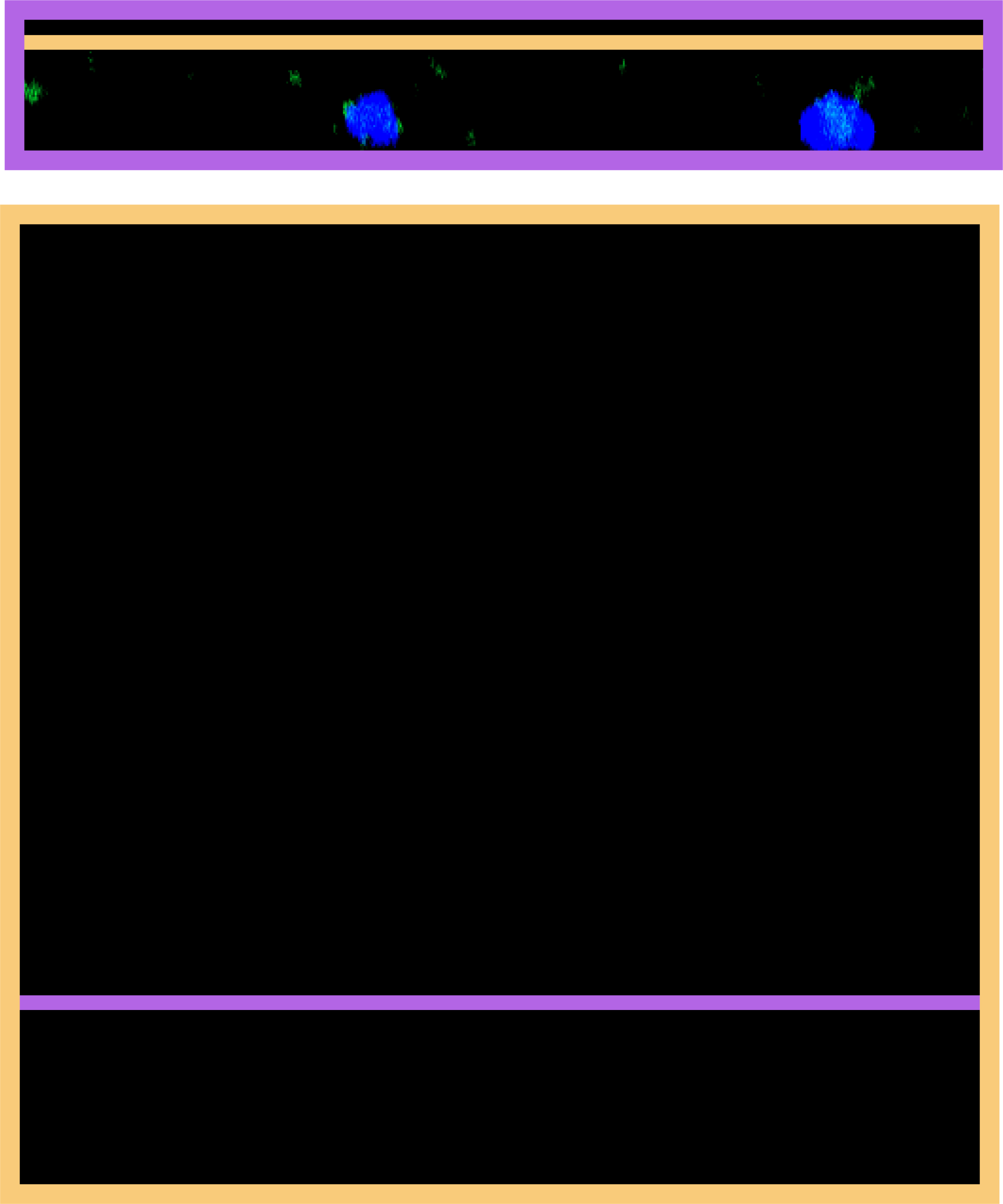

Supplement: Supplementary file 8 — Source Data for Figure 2 [file EMBJ-42-e111383-s003.zip › Source Data for Figure 2/Fig. 2f/Fig. 2f.tif]

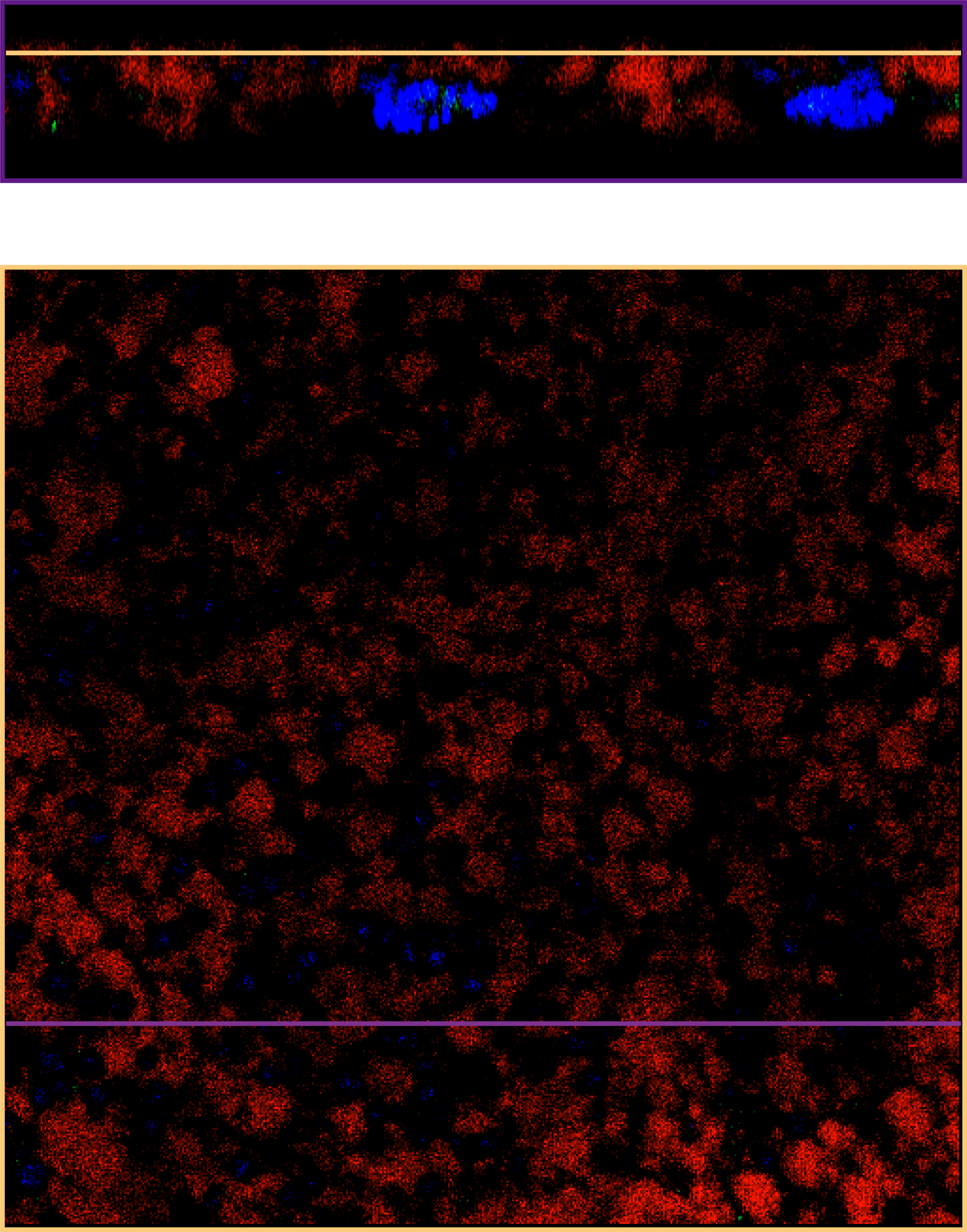

Supplement: Supplementary file 8 — Source Data for Figure 2 [file EMBJ-42-e111383-s003.zip › Source Data for Figure 2/Fig. 2a/Fig. 2a.tif]

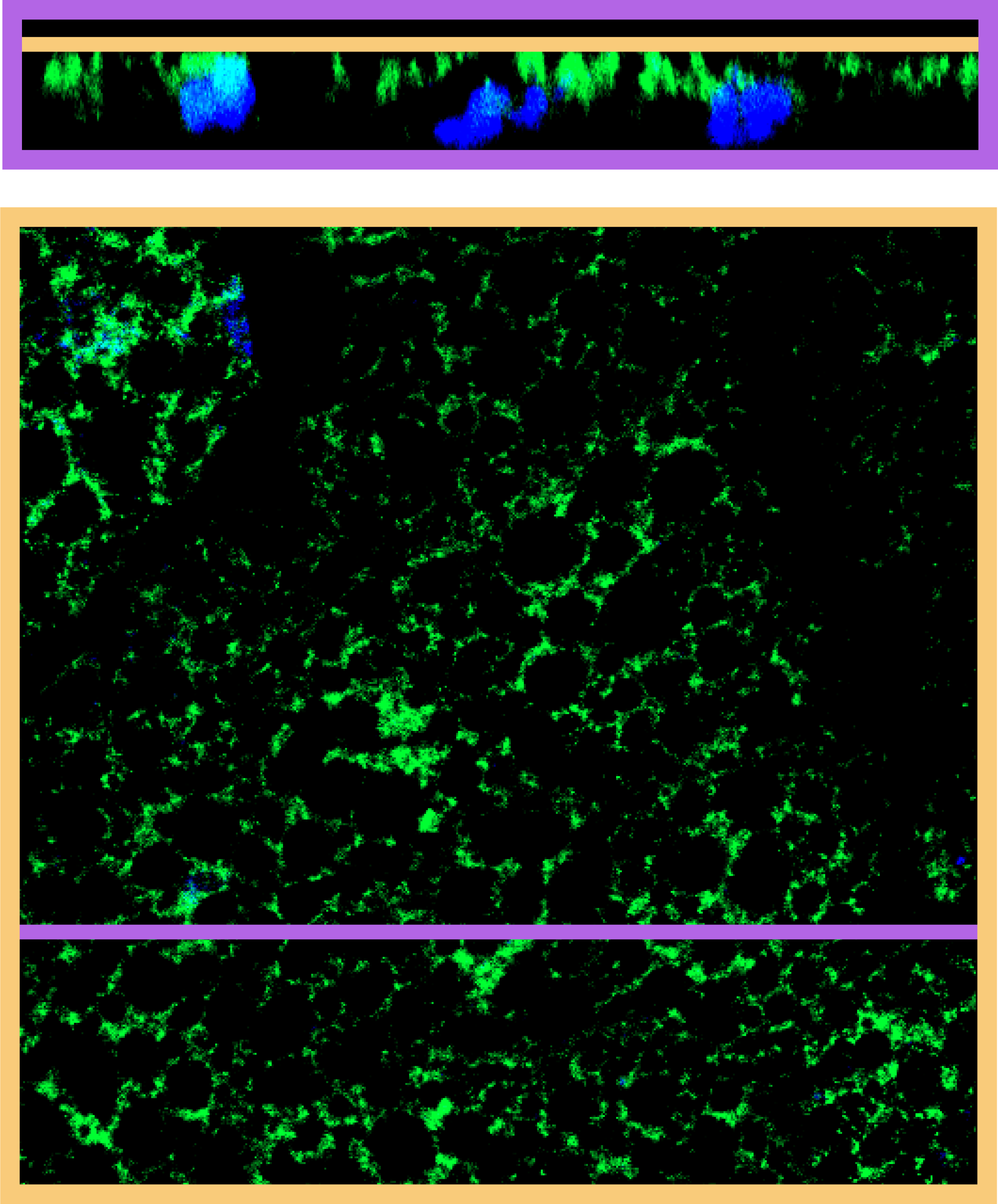

Supplement: Supplementary file 8 — Source Data for Figure 2 [file EMBJ-42-e111383-s003.zip › Source Data for Figure 2/Fig. 2h/Fig. 2h.tif]

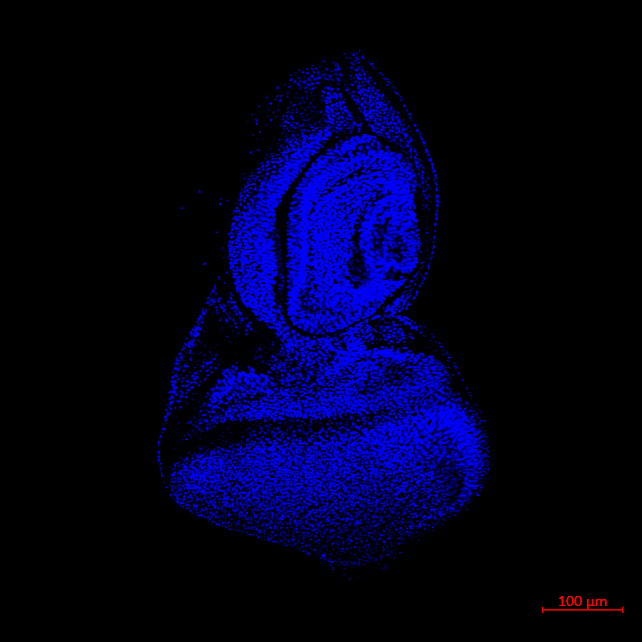

Supplement: Supplementary file 8 — Source Data for Figure 2 [file EMBJ-42-e111383-s003.zip › Source Data for Figure 2/Fig. 2g/Fig. 2g_DAPI.tif]

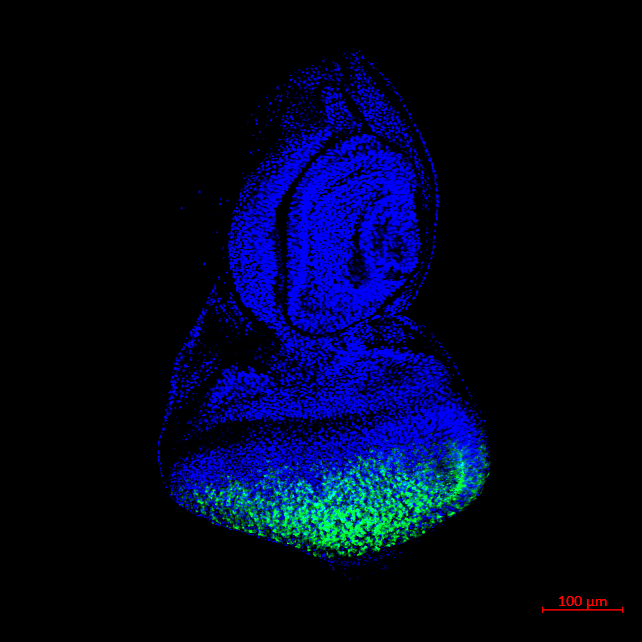

Supplement: Supplementary file 8 — Source Data for Figure 2 [file EMBJ-42-e111383-s003.zip › Source Data for Figure 2/Fig. 2g/Fig. 2g_Merge.tif]

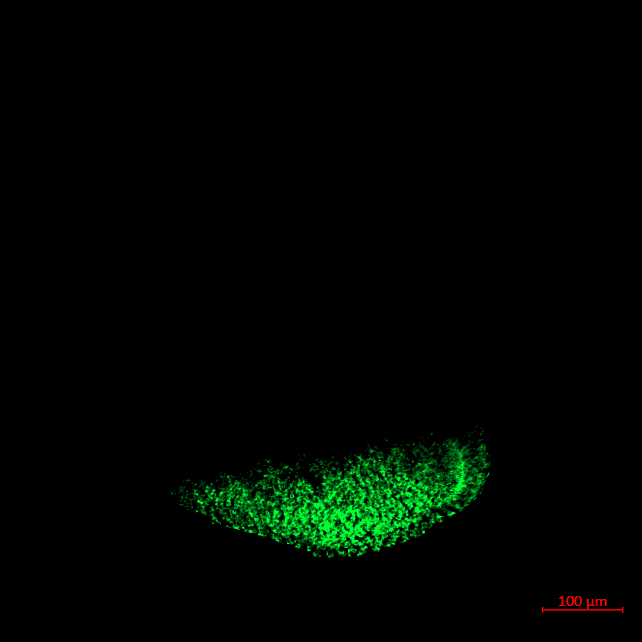

Supplement: Supplementary file 8 — Source Data for Figure 2 [file EMBJ-42-e111383-s003.zip › Source Data for Figure 2/Fig. 2g/Fig. 2g_GFP.tif]

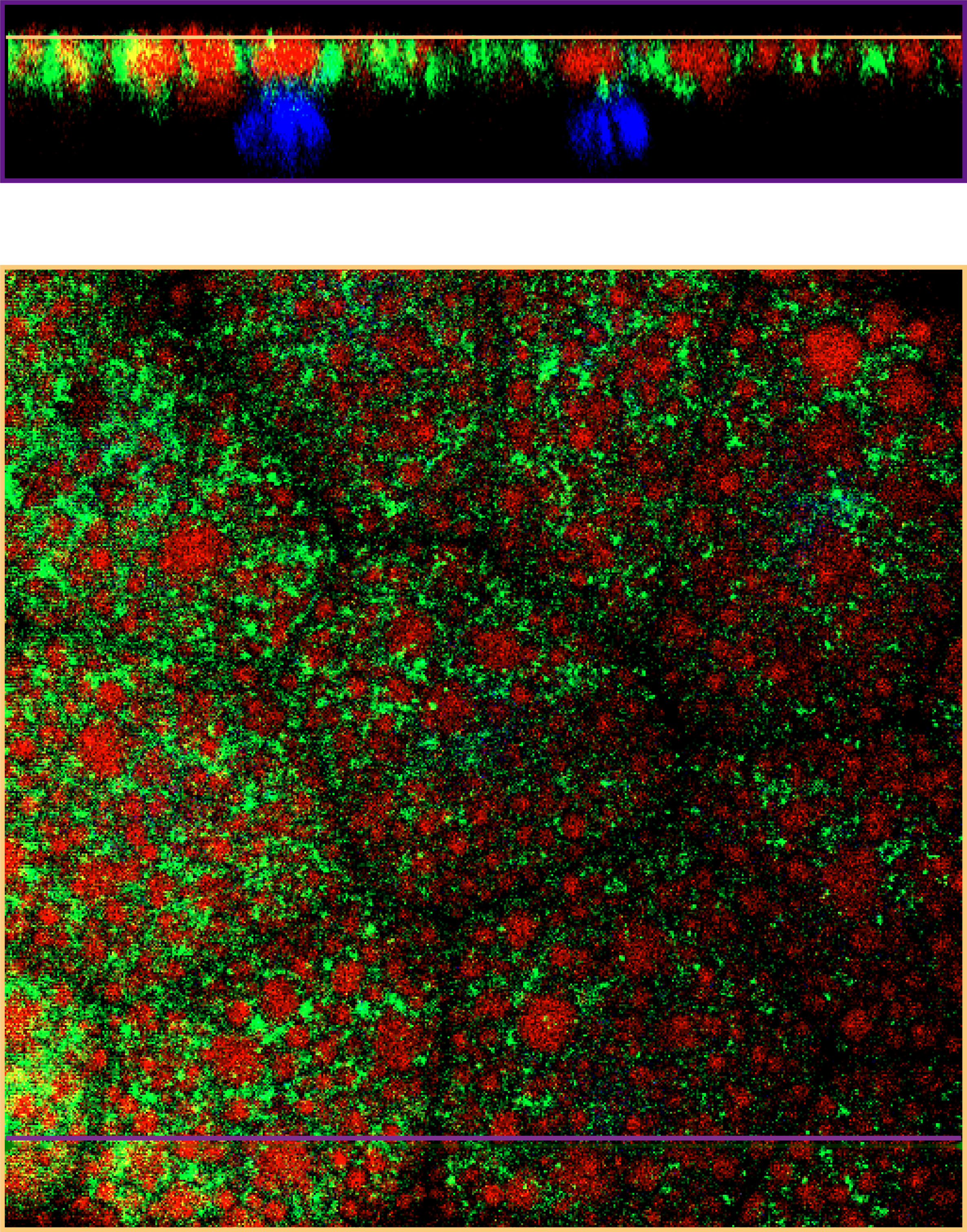

Supplement: Supplementary file 8 — Source Data for Figure 2 [file EMBJ-42-e111383-s003.zip › Source Data for Figure 2/Fig. 2b/Fig. 2b.tif]

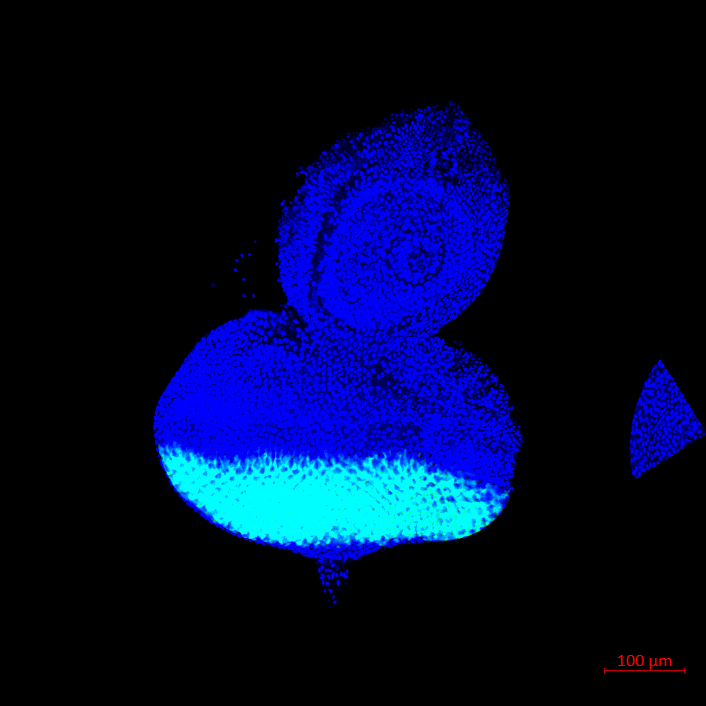

Supplement: Supplementary file 8 — Source Data for Figure 2 [file EMBJ-42-e111383-s003.zip › Source Data for Figure 2/Fig. 2e/Fig. 2e_Merge.tif]

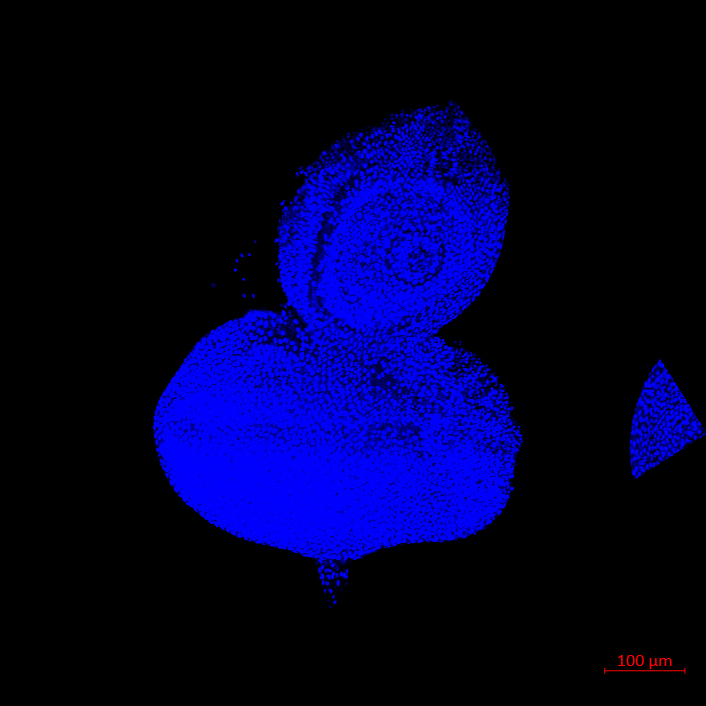

Supplement: Supplementary file 8 — Source Data for Figure 2 [file EMBJ-42-e111383-s003.zip › Source Data for Figure 2/Fig. 2e/Fig. 2e_DAPI.tif]

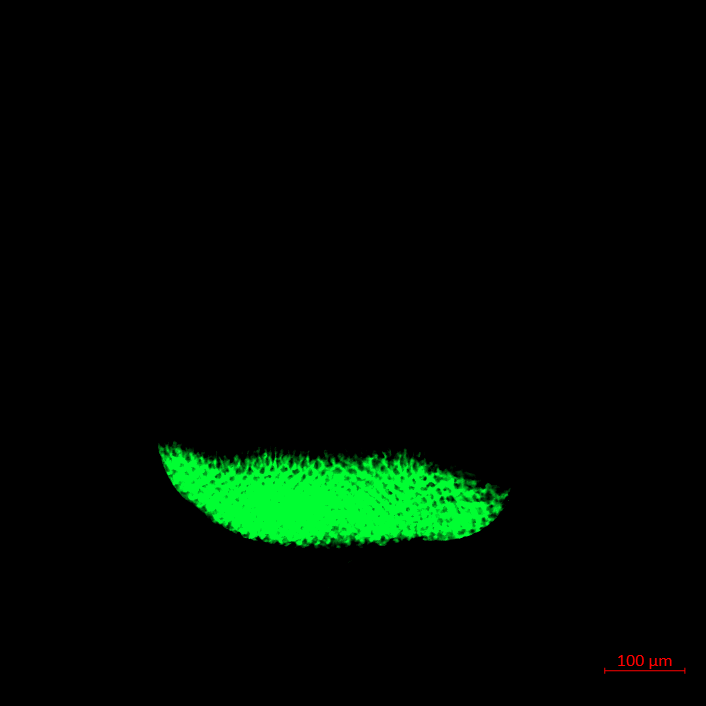

Supplement: Supplementary file 8 — Source Data for Figure 2 [file EMBJ-42-e111383-s003.zip › Source Data for Figure 2/Fig. 2e/Fig. 2e_GFP.tif]

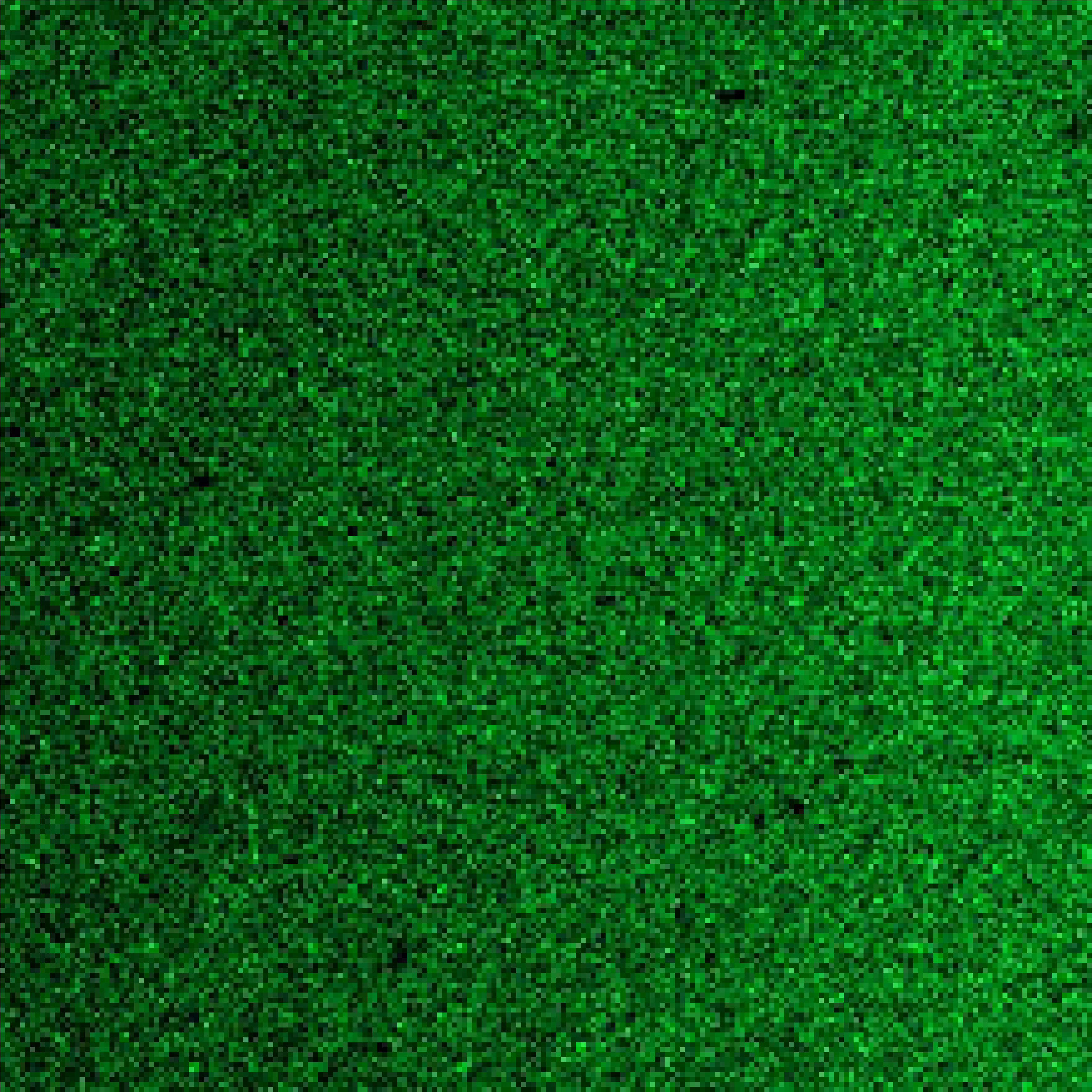

Supplement: Supplementary file 8 — Source Data for Figure 2 [file EMBJ-42-e111383-s003.zip › Source Data for Figure 2/Fig. 2k/Fig. 2k.tif]

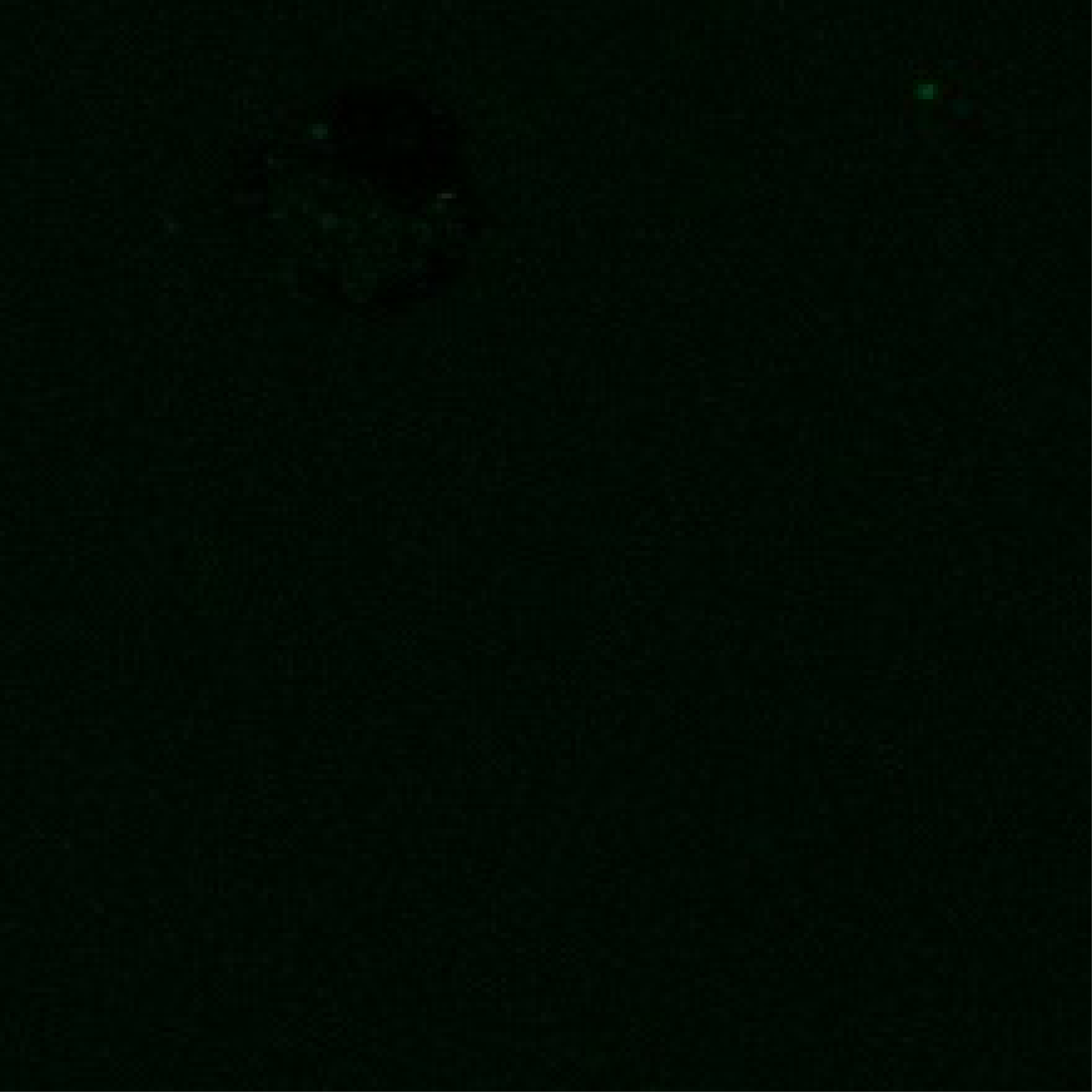

Supplement: Supplementary file 8 — Source Data for Figure 2 [file EMBJ-42-e111383-s003.zip › Source Data for Figure 2/Fig. 2j/Fig. 2j.tif]

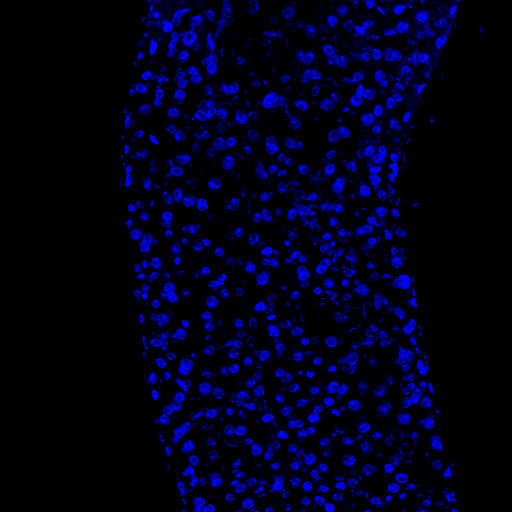

Supplement: Supplementary file 11 — Source Data for Figure 5 [file EMBJ-42-e111383-s006.zip › Source Data for Figure 5/Fig. 5f/Fig. 5f_DAPI.tif]

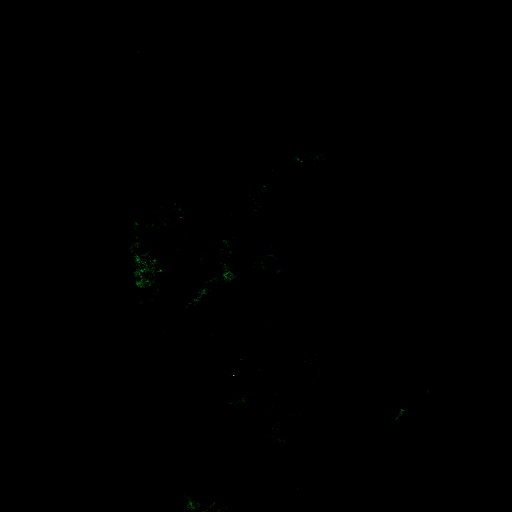

Supplement: Supplementary file 11 — Source Data for Figure 5 [file EMBJ-42-e111383-s006.zip › Source Data for Figure 5/Fig. 5f/Fig. 5f_GFP.tif]

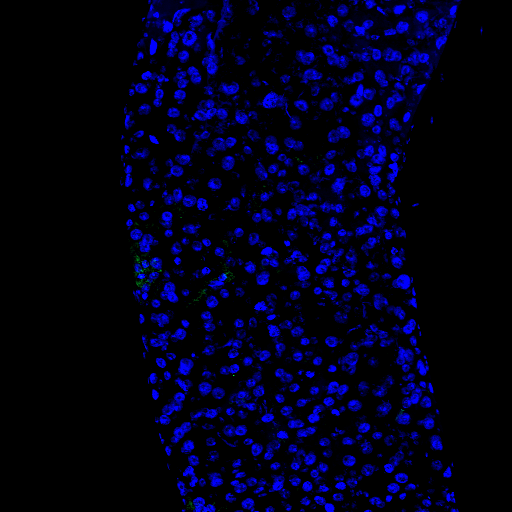

Supplement: Supplementary file 11 — Source Data for Figure 5 [file EMBJ-42-e111383-s006.zip › Source Data for Figure 5/Fig. 5f/Fig. 5f_Merge.tif]

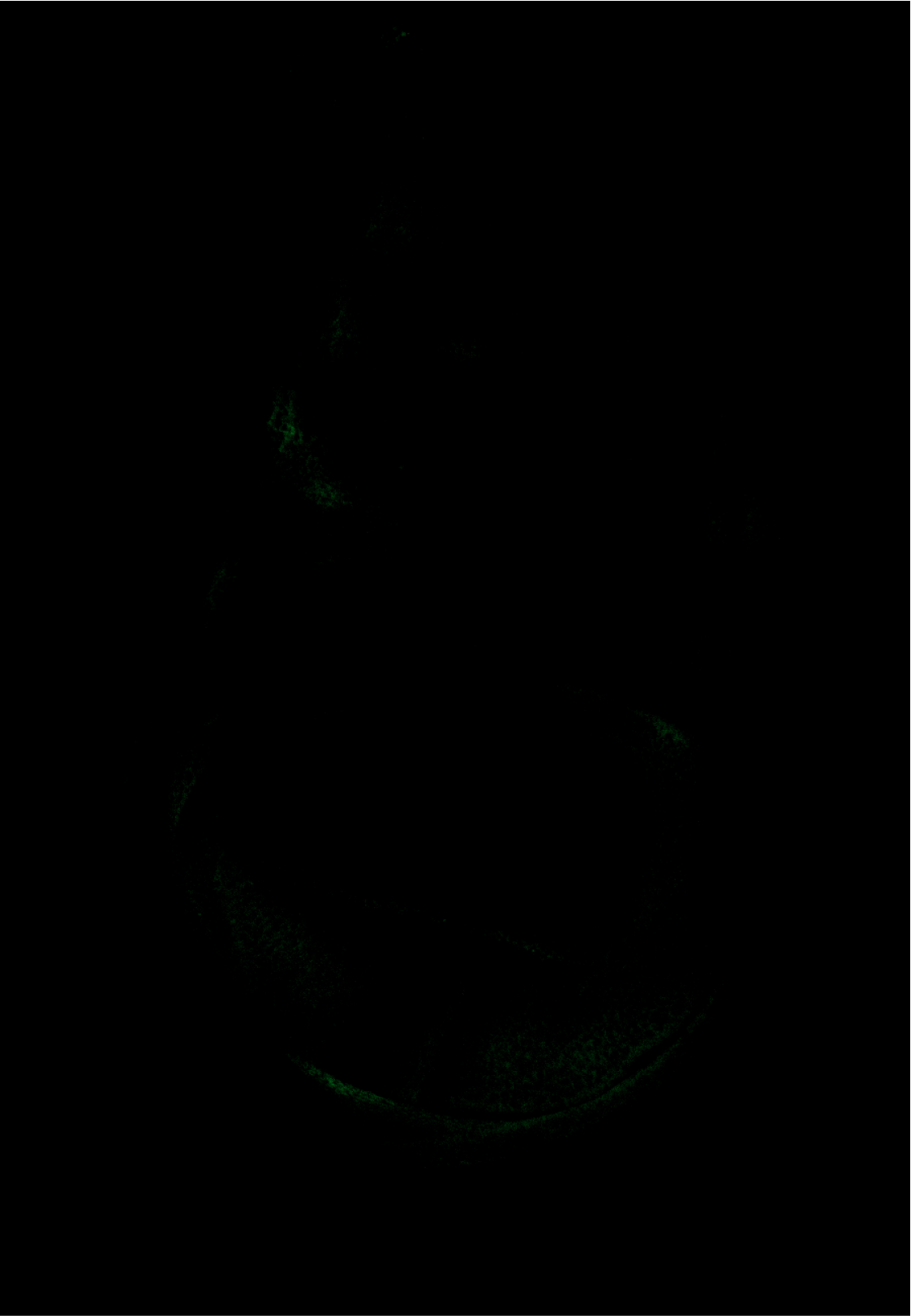

Supplement: Supplementary file 11 — Source Data for Figure 5 [file EMBJ-42-e111383-s006.zip › Source Data for Figure 5/Fig. 5a/Fig. 5a_NetB.tif]

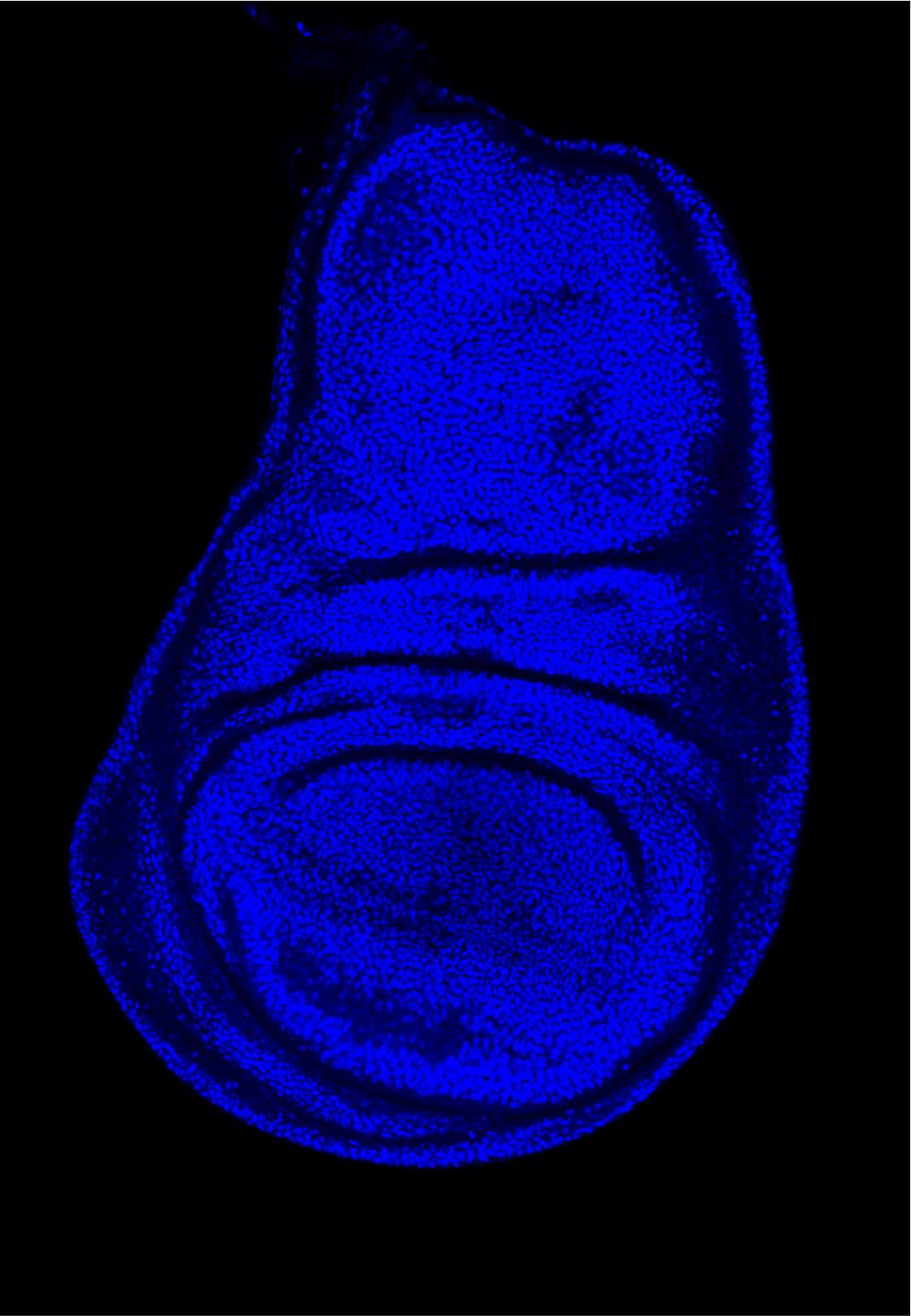

Supplement: Supplementary file 11 — Source Data for Figure 5 [file EMBJ-42-e111383-s006.zip › Source Data for Figure 5/Fig. 5a/Fig. 5a_DAPI.tif]

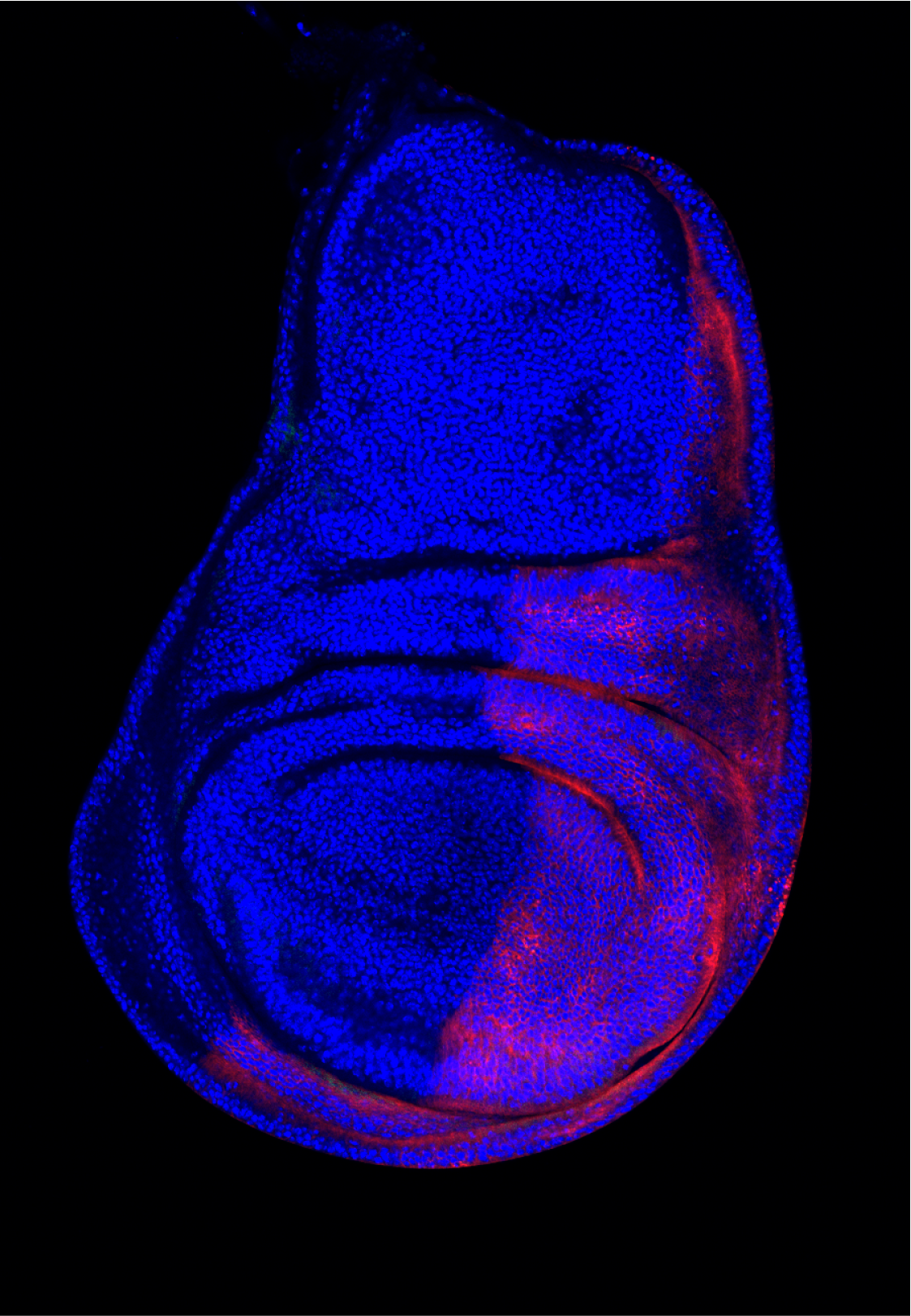

Supplement: Supplementary file 11 — Source Data for Figure 5 [file EMBJ-42-e111383-s006.zip › Source Data for Figure 5/Fig. 5a/Fig. 5a_Merge.tif]

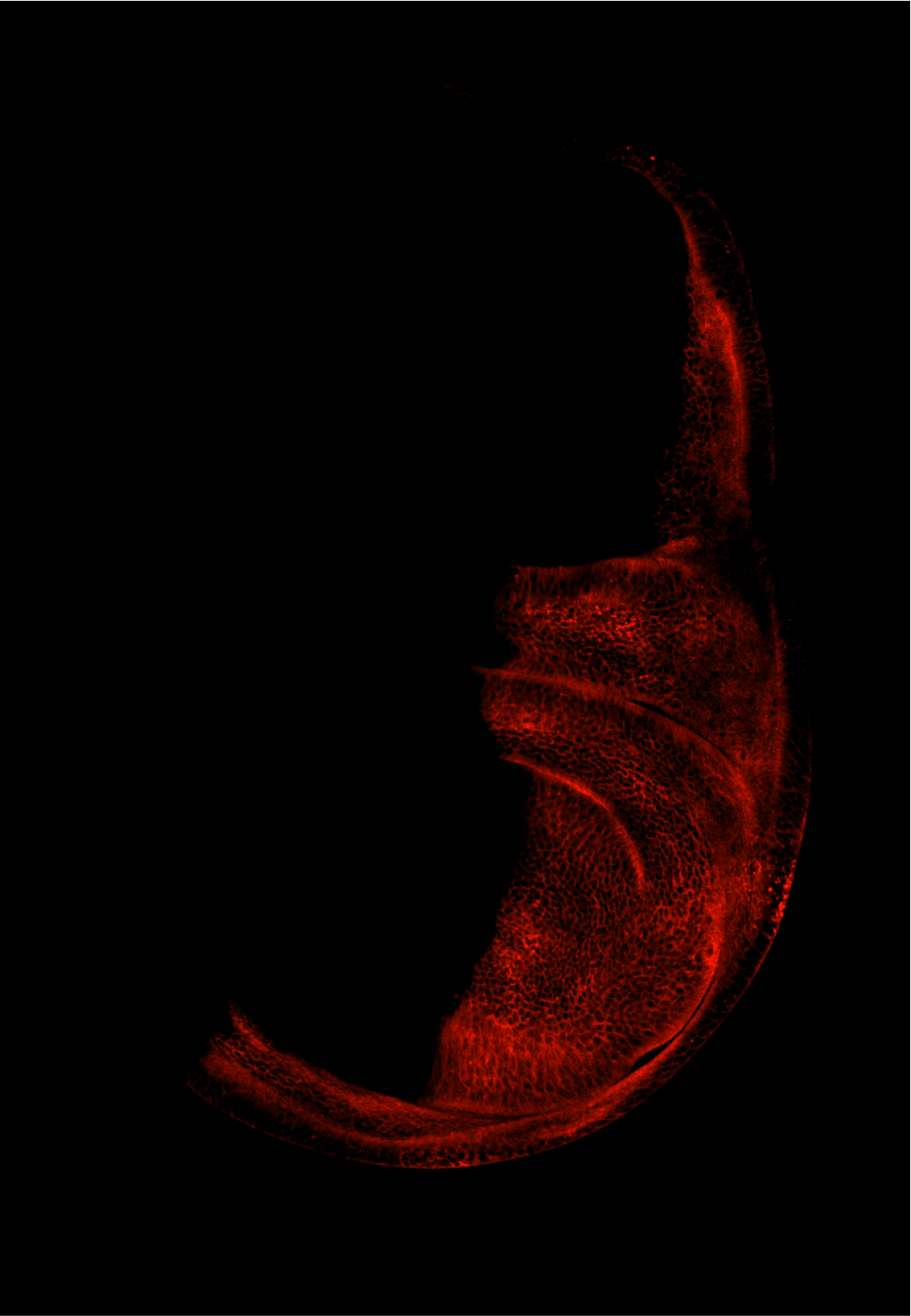

Supplement: Supplementary file 11 — Source Data for Figure 5 [file EMBJ-42-e111383-s006.zip › Source Data for Figure 5/Fig. 5a/Fig. 5a_RFP.tif]

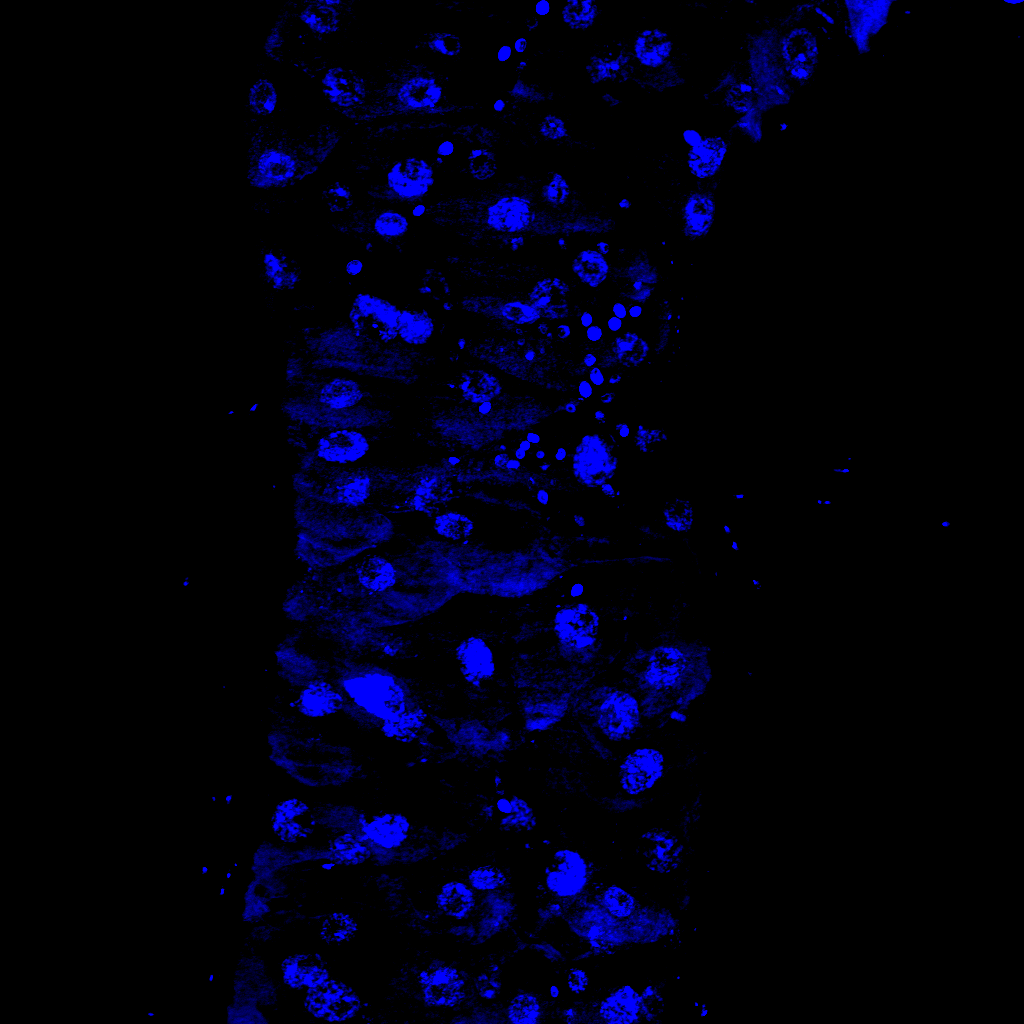

Supplement: Supplementary file 11 — Source Data for Figure 5 [file EMBJ-42-e111383-s006.zip › Source Data for Figure 5/Fig. 5g/Fig. 5g_DAPI.tif]

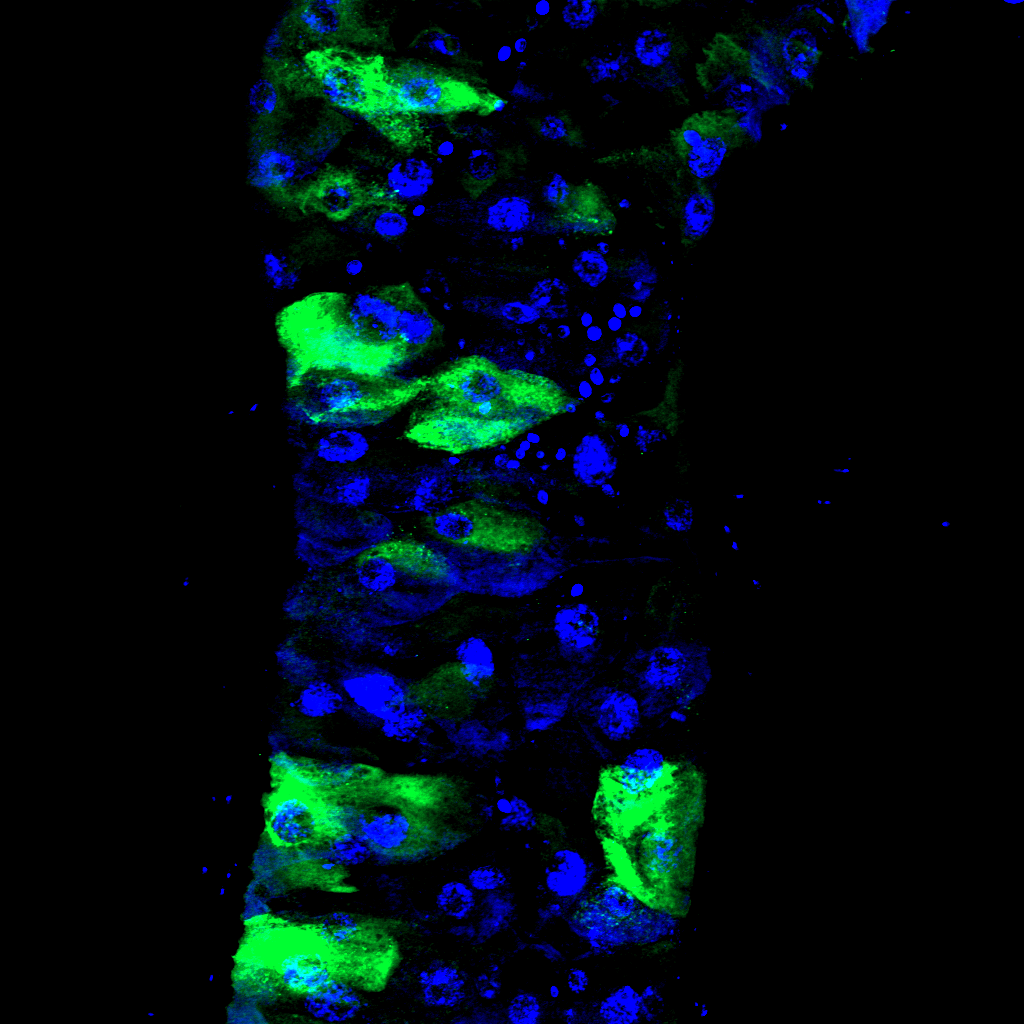

Supplement: Supplementary file 11 — Source Data for Figure 5 [file EMBJ-42-e111383-s006.zip › Source Data for Figure 5/Fig. 5g/Fig. 5g_Merge.tif]

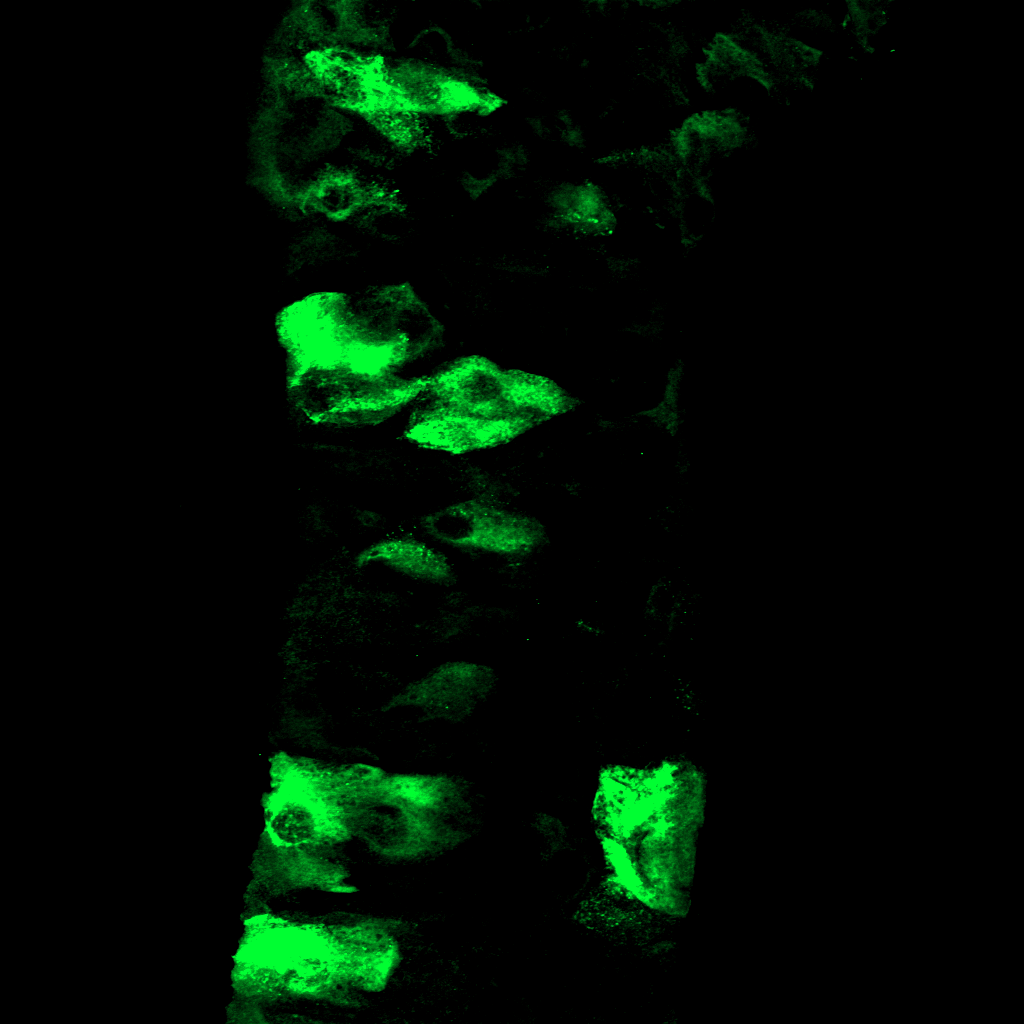

Supplement: Supplementary file 11 — Source Data for Figure 5 [file EMBJ-42-e111383-s006.zip › Source Data for Figure 5/Fig. 5g/Fig. 5g_GFP.tif]

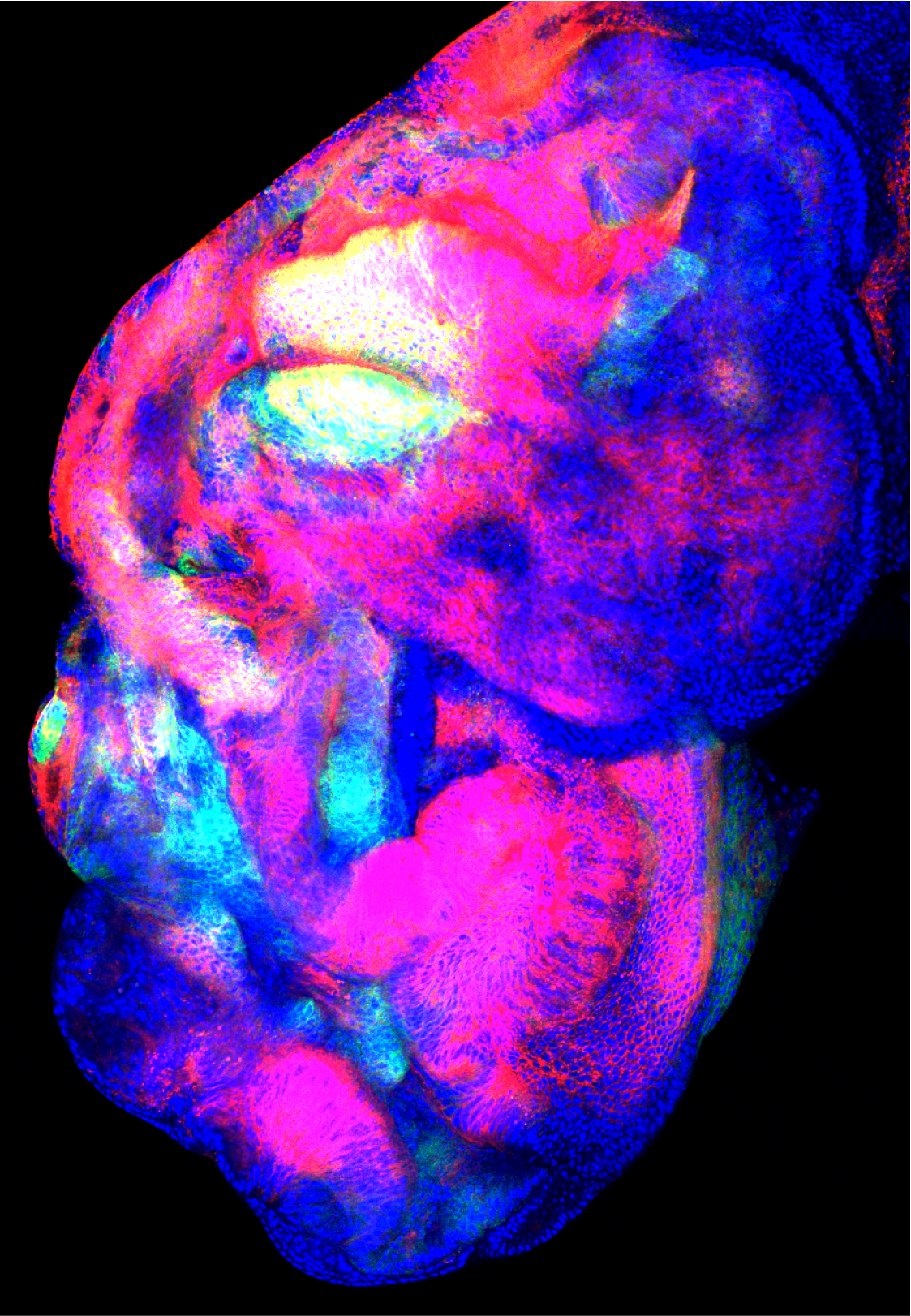

Supplement: Supplementary file 11 — Source Data for Figure 5 [file EMBJ-42-e111383-s006.zip › Source Data for Figure 5/Fig. 5b/Fig. 5b_Merge.tif]

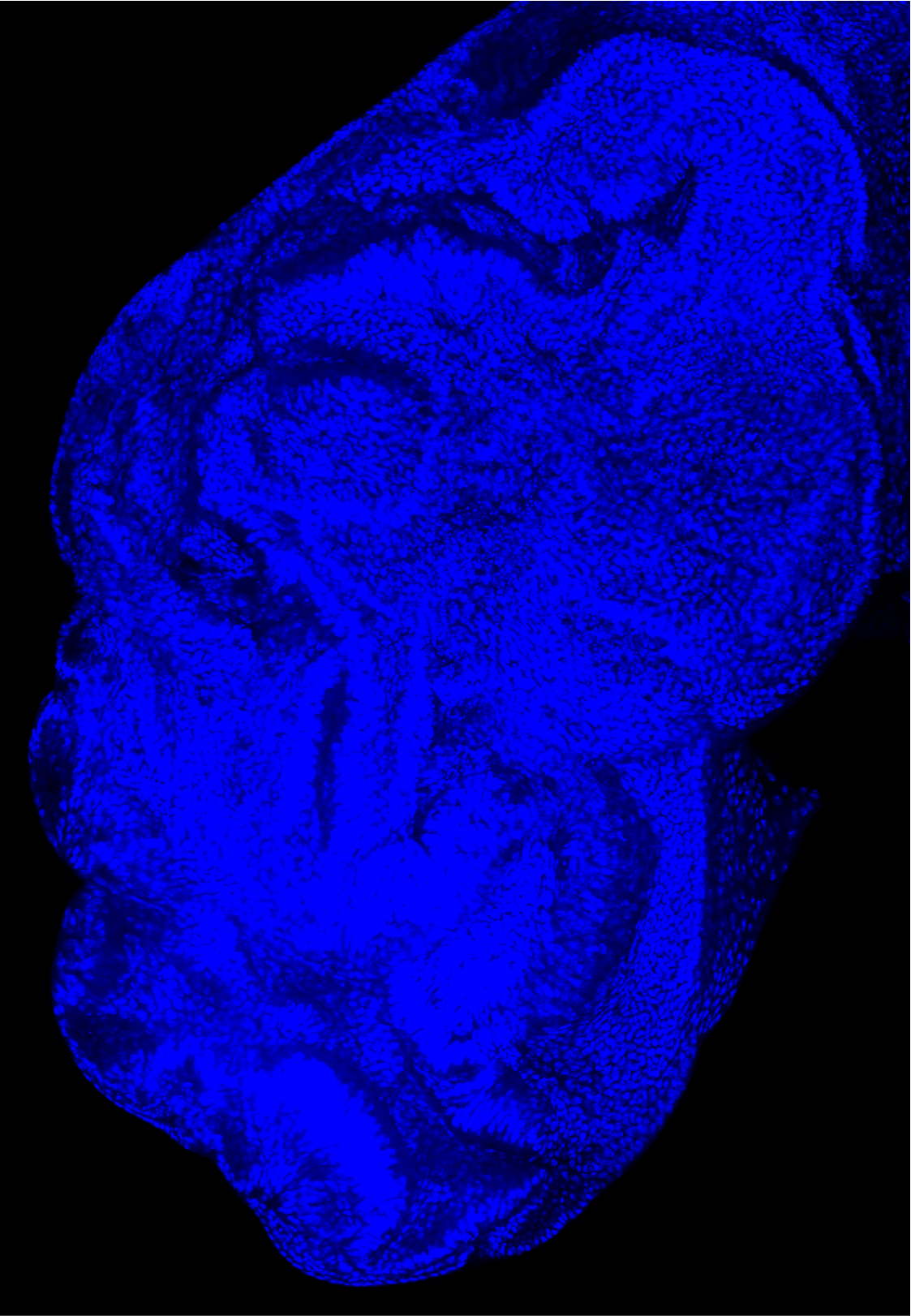

Supplement: Supplementary file 11 — Source Data for Figure 5 [file EMBJ-42-e111383-s006.zip › Source Data for Figure 5/Fig. 5b/Fig. 5b_DAPI.tif]

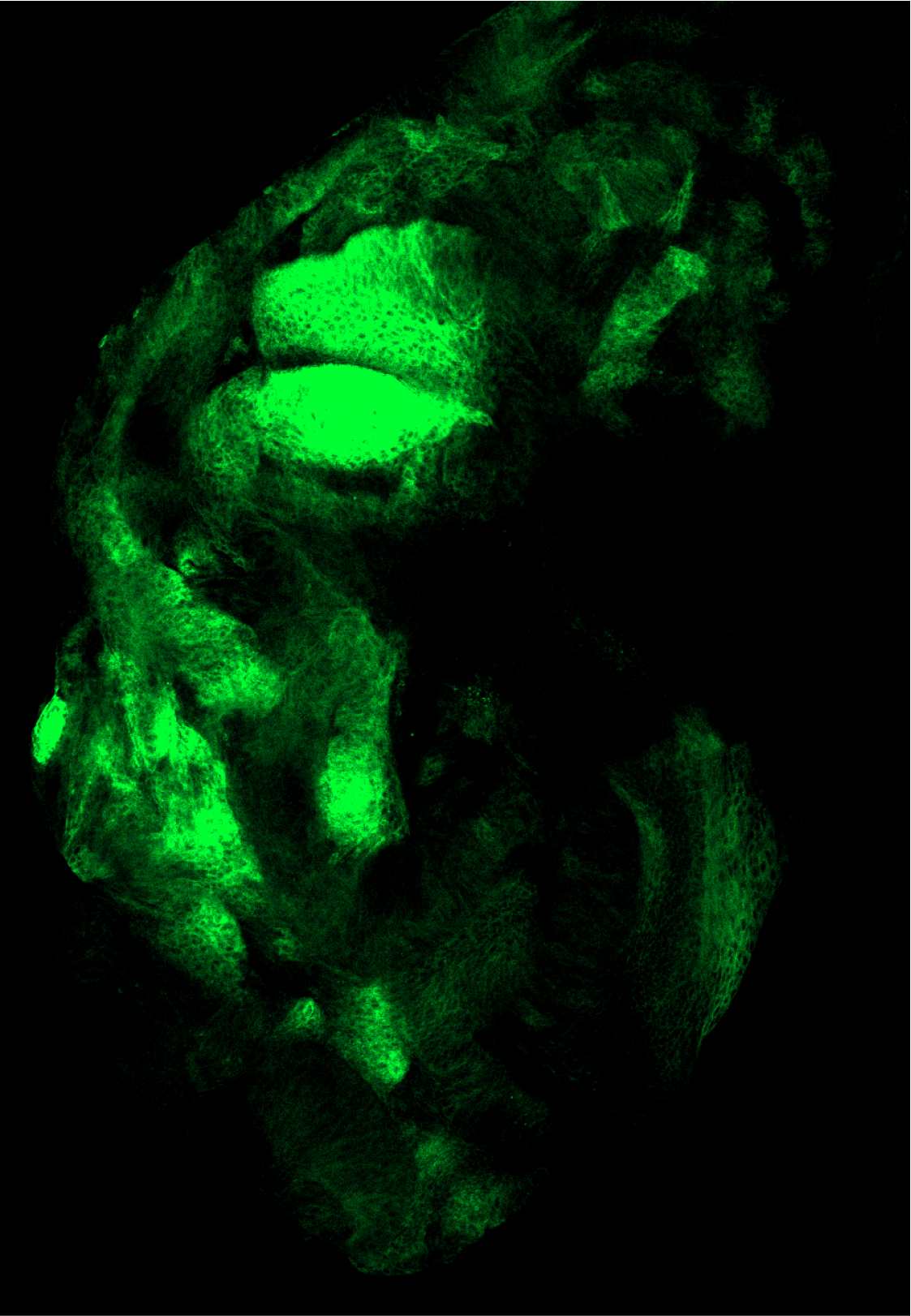

Supplement: Supplementary file 11 — Source Data for Figure 5 [file EMBJ-42-e111383-s006.zip › Source Data for Figure 5/Fig. 5b/Fig. 5b_NetB.tif]

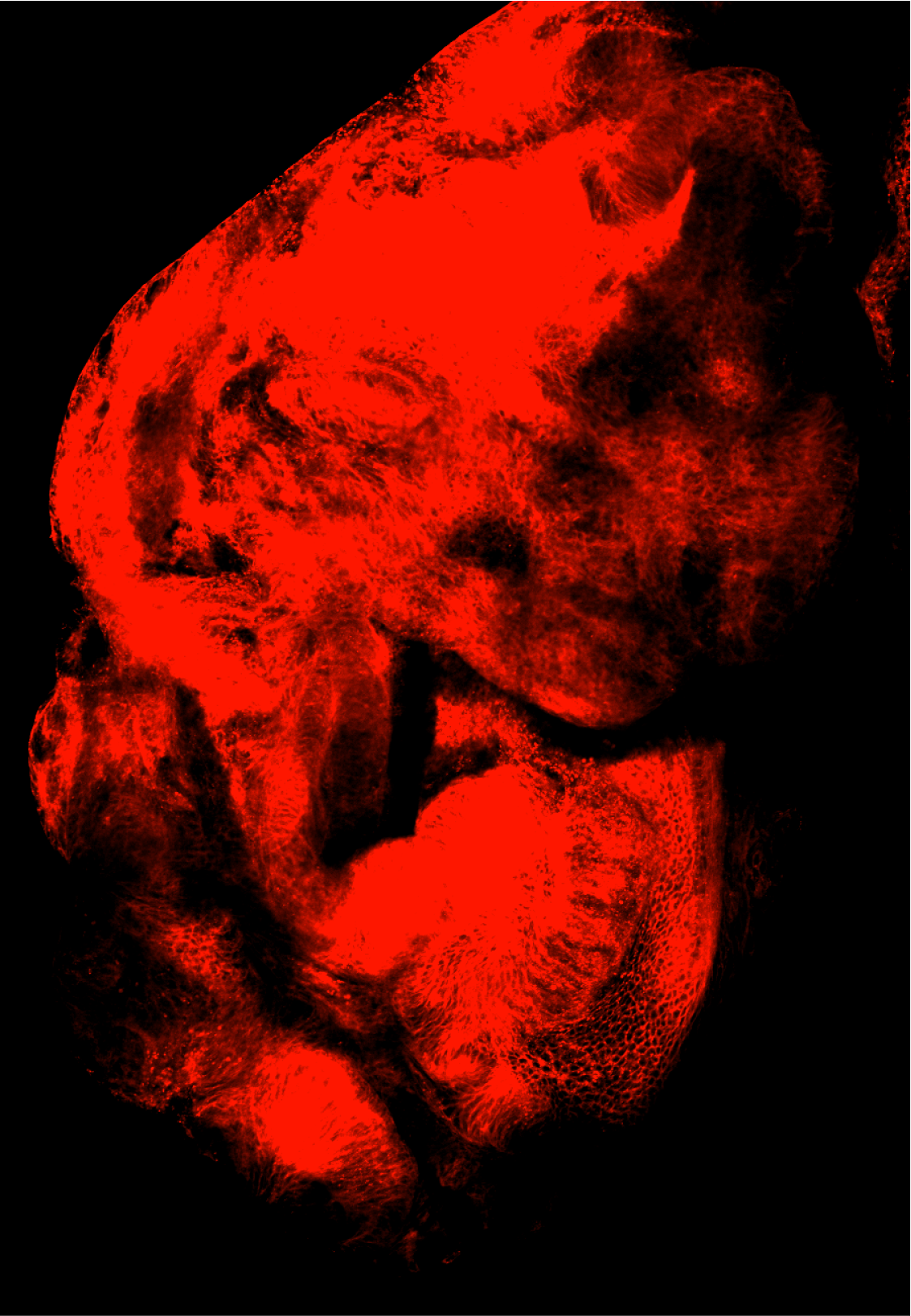

Supplement: Supplementary file 11 — Source Data for Figure 5 [file EMBJ-42-e111383-s006.zip › Source Data for Figure 5/Fig. 5b/Fig. 5b_RFP.tif]
