# Supplementary material for: Oncogenic stress‐induced Netrin is a humoral signaling molecule that reprograms systemic metabolism in Drosophila
Source: EMBO J. 2023 May 4;42(12):e111383. doi: 10.15252/embj.2022111383 (PMC10267689; doi:10.15252/embj.2022111383)
Supplement: Supplementary file 12 [file EMBJ-42-e111383-s010.pdf]

THE EMBO JOURNAL

Oncogenic stress-induced Netrin is a humoral signaling molecule that reprograms systemic metabolism in Drosophila

Morihiro Okada, Tomomi Takano, Yuko Ikegawa, Hanna Ciesielski, Hiroshi Nishida, and Sa Kan Yoo
DOI: 10.15252/embj.2022111383

Corresponding author(s): Sa Kan Yoo (sakan.yoo@riken.jp) , Morihiro Okada (morihiro.okada@riken.jp)

Review Timeline:

Pre-submission Date:	7th Apr 22
Pre-submission Editorial Decision:	8th Apr 22
Revision Received:	24th Jul 22
Editorial Decision:	19th Aug 22
Revision Received:	12th Feb 23
Editorial Decision:	27th Mar 23
Revision Received:	1st Apr 23
Accepted:	20th Apr 23

Editor: Ieva Gailite

Transaction Report:

(Note: With the exception of the correction of typographical or spelling errors that could be a source of ambiguity, letters and reports are not edited. Depending on transfer agreements, referee reports obtained elsewhere may or may not be included in this compilation. Referee reports are anonymous unless the Referee chooses to sign their reports.)

Presubmission Editorial Decision **8th Apr 2022**

Dear Dr. Yoo,

Thank you for submitting your pre-submission inquiry to The EMBO Journal. I have now read your summary and briefly discussed it with my colleagues. The topic of the manuscript certainly falls within the scope of our journal and we find the study very interesting. However, it is difficult to judge the extent of the analysis based on the limited information available. Therefore, I would like to invite you to submit the full manuscript using the link provided below. While submission of a full manuscript would mean that the manuscript could not be submitted elsewhere while under consideration here, I would get back to you with a final editorial decision within the next few days after full submission. I look forward to receiving your manuscript.

Yours sincerely,

Ieva Gailite

Ieva Gailite, PhD
Scientific Editor
The EMBO Journal
Meyerhofstrasse 1
D-69117 Heidelberg
Tel: +4962218891309
i.gailite@embojournal.org

To submit your manuscript, please follow this link.

Link Not Available

Please do not share this URL as it will give anyone who clicks it access to your account.

Please note that for technical reasons, this link will take you to a page for uploading revised manuscripts - once we receive the submission, it will be formally turned into an original submission.

1st Editorial Decision 19th Aug 2022

Dear Dr. Yoo,

Thank you for submitting your manuscript for consideration by the EMBO Journal. We have now received comments from three reviewers, which are included below for your information.

As you will see from the reports, the reviewers find the study novel and interesting, while also indicating several limitations of the used GMR-Gal4 expression system and the specific conditions of pupal stage metabolism. All reviewers agree that extension of the gut tumour system analysis would be needed to allow generalization of the proposed model.

If you find that you are able to address the main issues raised by the reviewers, I would be happy to consider a revised version of the manuscript. I think it would be helpful to discuss the revision in more detail via email or phone/videoconferencing - please let me know which option you prefer. I should also add that it is The EMBO Journal policy to allow only a single major round of revision and that it is therefore important to resolve the main concerns at this stage.

We generally allow three months as standard revision time. As a matter of policy, competing manuscripts published during this period will not negatively impact on our assessment of the conceptual advance presented by your study. However, please contact me as soon as possible upon publication of any related work to discuss the appropriate course of action. Should you foresee a problem in meeting this three-month deadline, please let us know in advance to arrange an extension.

When preparing your letter of response to the referees' comments, please bear in mind that this will form part of the Review Process File and will therefore be available online to the community. For more details on our Transparent Editorial Process, please visit our website: https://www.embopress.org/page/journal/14602075/authorguide#transparentprocess. Please also see the attached instructions for further guidelines on preparation of the revised manuscript.

Please feel free to contact me if you have any further questions regarding the revision. Thank you for the opportunity to consider your work for publication. I look forward to discussing your revision.

With best regards,

Ieva

Ieva Gailite, PhD
Senior Scientific Editor
The EMBO Journal
Meyerhofstrasse 1
D-69117 Heidelberg
Tel: +4962218891309
i.gailite@embojournal.org

When submitting your revised manuscript, please carefully review the instructions below and include the following items:

1) a .docx formatted version of the manuscript text (including legends for main figures, EV figures and tables). Please make sure that the changes are highlighted to be clearly visible.

2) individual production quality figure files as .eps, .tif, .jpg (one file per figure).

3) a .docx formatted letter INCLUDING the reviewers' reports and your detailed point-by-point response to their comments. As part of the EMBO Press transparent editorial process, the point-by-point response is part of the Review Process File (RPF), which will be published alongside your paper.

4) a complete author checklist, which you can download from our author guidelines (https://wol-prod-cdn.literatumonline.com/pb-assets/embo-site/Author Checklist%20-%20EMBO%20J-1561436015657.xlsx). Please insert information in the checklist that is also reflected in the manuscript. The completed author checklist will also be part of the RPF.

5) Please note that all corresponding authors are required to supply an ORCID ID for their name upon submission of a revised manuscript.

6) Before submitting your revision, primary datasets produced in this study need to be deposited in an appropriate public database (see https://www.embopress.org/page/journal/14602075/authorguide#datadeposition). Please remember to provide a reviewer password if the datasets are not yet public. Please note that the Data Availability Section is restricted to new primary data that are part of this study. If no data deposition in external databases is needed for this paper, please then state in this section: This study includes no data deposited in external repositories.
*** Note - All links should resolve to a page where the data can be accessed. ***

7) Our journal encourages inclusion of *data citations in the reference list* to directly cite datasets that were re-used and obtained from public databases. Data citations in the article text are distinct from normal bibliographical citations and should directly link to the database records from which the data can be accessed. In the main text, data citations are formatted as follows: "Data ref: Smith et al, 2001" or "Data ref: NCBI Sequence Read Archive PRJNA342805, 2017". In the Reference list, data citations must be labeled with "[DATASET]". A data reference must provide the database name, accession number/identifiers and a resolvable link to the landing page from which the data can be accessed at the end of the reference. Further instructions are available at .

8) We would also encourage you to include the source data for figure panels that show essential data. Numerical data can be provided as individual .xls or .csv files (including a tab describing the data). For 'blots' or microscopy, uncropped images should be submitted (using a zip archive or a single pdf per main figure if multiple images need to be supplied for one panel). Additional information on source data and instruction on how to label the files are available at .

9) We replaced Supplementary Information with Expanded View (EV) Figures and Tables that are collapsible/expandable online (see examples in https://www.embopress.org/doi/10.15252/embj.201695874). A maximum of 5 EV Figures can be typeset. EV Figures should be cited as 'Figure EV1, Figure EV2" etc. in the text and their respective legends should be included in the main text after the legends of regular figures.

- For the figures that you do NOT wish to display as Expanded View figures, they should be bundled together with their legends in a single PDF file called *Appendix*, which should start with a short Table of Content. Appendix figures should be referred to in the main text as: "Appendix Figure S1, Appendix Figure S2" etc. See detailed instructions regarding expanded view here: .

- Additional Tables/Datasets should be labelled and referred to as Table EV1, Dataset EV1, etc. Legends have to be provided in a separate tab in case of .xls files. Alternatively, the legend can be supplied as a separate text file (README) and zipped together with the Table/Dataset file.

10) When assembling figures, please refer to our figure preparation guideline in order to ensure proper formatting and readability in print as well as on screen:
http://bit.ly/EMBOPressFigurePreparationGuideline

Please remember: Digital image enhancement is acceptable practice, as long as it accurately represents the original data and conforms to community standards. If a figure has been subjected to significant electronic manipulation, this must be noted in the figure legend or in the 'Materials and Methods' section. The editors reserve the right to request original versions of figures and the original images that were used to assemble the figure.

Further information is available in our Guide For Authors: https://www.embopress.org/page/journal/14602075/authorguide

We realize that it is difficult to revise to a specific deadline. In the interest of protecting the conceptual advance provided by the work, we recommend a revision within 3 months (17th Nov 2022). Please discuss the revision progress ahead of this time with the editor if you require more time to complete the revisions.

Referee #1:

Okada M, et al seek to find how tumorkines may impact tumor-induced animal health deterioration and survival using flies. Expression of oncogenic RasV12 in differentiating cells of the eye part of the eye-antennal disc leads to a malformed (rough) eye due to developmental abnormalities. Despite the fact that all larvae reach the pupal stage with normal timing, only some hatch to adulthood. The lack of hatching is defined by the authors as survival from the oncogenic insult. Herein, lies my main concern. It is unclear whether this survival is similar to adult survival or is a particularity of the pupal stage which has specific energetic (non-feeding state), gross morphogenetic alterations and growth processes.
In this framework, 20 induced potential tumorkines were identified by RNASeq. One of these genes, netrin-B, is inducedalso at the protein levels and knockdown of the netrin-B expression improved hatching rate when knocked down in the tumor.

Overexpression of netrin-B alone from the eye-antennal disc is sufficient to cause lethality (lack of hatching) showing that NetrinB is both required and adequate to inhibit hatching. Surprisingly, both endogenous NetrinB and tagged NetrinB from expressed in the eye disc are secreted systemically and accumulate in the Fat Body upon RasV12 overexpression only. Knocking down the Unc-5 netrin receptor (Unc-5) in the fat body, but not other netrin receptors (frazzled or Dscam1) increases survival, suggesting that netrin impacts the fat body directly to induced lethality. Increased survival is also seen when Insulin signaling in the fat body is reduced and this allowed the authors to identify levels of TMLHE as a critical enzyme for tumor stress survival using RNASeq and genetics. TMLHE takes part in generating carnitine which is known for its role in fatty acid import to mitochondria , oxidation and energy generation. How TMLHE expression is controlled by NetrinB-Unc-5 and Insulin signaling remains unknown. It is also unclear whether reduced fatty acid oxidation in the fat body is what causes lethality in the pupal stage. The main limitation of the manuscript is that the experiments addressing whether this applies outside of the pupal stage for more "conventional" survival is not completed.
The novelty of this exciting manuscript lies in the identification NetrinB-Unc5 signaling in controlling remote metabolism affecting life span.
The manuscript is clearly written, and experiments are generally well designed according to the standard of the field.
Comments to authors:
General: Please provide source data, numbers of repeats etc for all graphs and figures. It is not possible to fully appreciate and evaluate the data and experimentation without it. The following comments are made assuming that the data are correctly obtained and processed. Please also deposit the RNASeq source and ppm data, so that they can be evaluated.

Fig 1. The tumor model(s): The authors define survival as the capacity to exit the pupal stage to adulthood (hatching). As a first approximation, it is unclear how pupal lethality reflects tumor-induced health deterioration in animals as such. The pupal stage is a particular non-feeding state where much of the animal organs are partially broken down and new tissues are formed. The death susceptibility could therefore be due to a developmental or energetic demand specific to this particular developmental stage or processes.
-Could the authors experimentally address whether the survival of the hatched tumor-carrying GMR-RasV12-escapers is reduced relative to control animals?
-To partially amend this problem, the authors use an adult RasV12-driven gut tumor model initiated from stem cells (Fig S5). It is, however, still unclear how similar the situations are. Is NetB induced in RasV12 gut tumor cells in adults? Please perform immunostaining of NetB. Does NetB knockdown in RasV12 gut tumors increase survival? Similarly, unc-5 fat body knockdown should increase survival. If this applies, it will strengthen the role of NetB-Unc5-TMLHE in reducing health and survival apart from the particular situation of the pupal stage.
-Fig1g-h. NetB has been shown be involved in neuronal differentiation and axonal guidance. Is NetB a general stress-induced tumorkine outside the neural system? This could easily be adressed by NetB immunostaining of animals with hs-flp induced RasV12 expressing MARCM clones ahead of the morphogenic furrow or antennal part. Other tissues can also be scored (brain, wing disc, gut,..)
-Fig1 c,d,e. Is it any particular reason why relative survival rate rather than absolute survival (hatching) as in Fig 1a?
Fig 2. A minor but interesting question is whether Netrin-B localization to the fat body depends on Unc-5 expression. This would suggest a ligand trapping mechanism. A simple immunostaining of NetB with or w/o FB-Gal4 > Unc-5-RNAi should answer this.
Fig3. Genetic experiments support a model whereby RasV12 remotely suppresses TMLHE mRNA levels in the fat body through Netrin-B-Unc-5 and that this is reversed upon suppression of the Insulin signaling pathway. It remains unknown how TMLHE may be regulated transcriptionally downstream of the receptor levels. Fig3 B,C. Does knockdown of the insulin receptor in the fat body lead to higher levels of TMLHE transcription alone?
Fig 4. Fig 4 a-e. The authors show convincingly using genetics that TMLHE is limiting in the fat body for survival to adulthood only for animals with RasV12 expression in the eye (gmr-RasV12). This is suggested to be related to carnitine biosynthesis needed for fatty acid import to mitochondria for energy generation. This is supported by the results that show increased survival by supplying carnitine or an acetyl-CoA precursor increase survival.
However, if this model is correct, two predictions should hold true:
1. Knocking down crucial components of the carnitine shuttle/fatty acid import should have the same reduced survival, like knockdown of Cpt1 or Cpt2 in the fat body. For instance.
Gmr-RasV12, CG-Gal4 >Cpt1 (or Cpt2)-RNAi versus CG-Gal4>Cpt1 (or Cpt2)-RNAi (see PMID: 33371457)
2. Knocking down TMLHE in the fat body should be limiting for ATP levels, possibly only in the Gmr-RasV12 background. I encourage the authors to elaborate on these experiments.
Fig 4g. To try and address whether TMLHE extends to affect death susceptibility other than at the pupal stage, which may have particular developmental or energetic demands, the authors resort to an adult gut tumor model driven by RasV12 in the stem cells. Esg>RasV12 animals display more than 50% reduction of life span compared to control. Knocking down TMLHE in the fat body further decreases this life span. However, control animals with TMLHE knockdown alone in the fat body is lacking to see if the reduction is additive or dependent on tumor presence. Further, it needs to be established whether NetB is expressed in Esg>RasV12 gut cells and whether NetB knockdown in the fat body is sufficient to extend life span under oncogenic stress. It may of course, be other factors involved in reducing adult health and life span than NetB, like the recently published Upd-Dome blood-brain barrier disruption in another tumor model (PMID: 34496290), so a complete rescue of reduced life span is not expected.

Referee #2:

Okada et al present a new system that studies oncogenic stress caused by a Drosophila RasV12 eye disc, and identify a new organ-organ signaling mechanism mediated by NetrinB that controls metabolism in the fat body. This molecule has been previously functioning in local signaling for neuron pathfinding. It is therefore a surprising finding that this paper reports and genetic and feeding experiments demonstrate it elegantly. Larvae with RasV12 eye discs make pupae, but infrequently eclose. The authors show this can be rescued by blocking NetB signaling to fat body. The authors identify carnitine metabolism downstream of NetB/Unc-5 through a secondary genetic screen for genes that are differentially regulated with inhibition of insulin signaling in the fat body with RasV12 eye discs. The authors screen point to TMHLE as being transcriptionally downregulated by NetB action, leading to reduced carnitine biosynthesis in the pupae. The authors are able to rescue eclosure rates by changing TMHLE expression or with carnitine supplementation in diet. The authors attempt to extend these findings to a RasV12 gut tumor model in the adult fly.
There is an exciting hypothesis presented in this paper that Netrins have a conserved function in organ-organ signaling and control metabolism in addition to neuronal connections. The studies the authors cite in human cancer elicit an attractive proposal that these findings can be broadly applied to understand death from oncogenic stress. However, I have concern that the data could also be interpreted as phenomenological to fly metamorphosis, which would narrow the impact. To achieve the broader relevance that the authors proposed in discussion, I would recommend some additions to the manuscript outlined below.

Major Criticisms
1. The authors use eclosure rate as measure of survival. It is not clear to me that failure of metamorphosis is the same as death of animal due to oncogenic stress. Development of the pupae into a adult requires significant and maybe very specific regulation of organ-organ signaling and metabolism that could be derailed by manipulations of the authors. The paper should write about this point rather than just using the word survival.
2. To make a case for NetB signaling mechanism being applied generally to oncogenic stress killing, the authors should extend the interesting studies in the adult RasV12 gut model, most of which should be easy:
A. Is NetB upregulated in the adult gut tumor? This should be easily ascertained from RNA or protein measurement or from microarray dataset (Tsuda-Sakurai et al 2020).
B. The experiments of Fig. 4G are missing important controls to show that RNAi of TMLHE in normal flies does not make them ill and die quicker, and a control RNAi expressed in the tumor model does not change survival.
C. The authors could test whether carnitine or acetyl COA supplementation extends life of gut tumor flies.
D. Additionally, measure carnitine levels in adult fat body with and without tumor.
E. NetB-Unc5 pathway could be directly tested between the gut tumor and the fat body through NetB RNAi in the tumor, and/or Unc5 RNAi in the fat body.
3. Measurements for larval survival assays are sometimes presented as a %age survival and other times as rate normalized to the control. It is difficult to compare the effect between these two different measurement units. I do not understand why for graphs with normalized survival rate, the data points bin into discreet groups rather than having more of a continuous spread. I am surprised by much larger survival rescue with Unc-5 heterozygotes (Figure 2N) compared to NetB RNAi (Figure 1C). If they are acting in the same signaling pathway, I would expect the effect to be less disparate, but perhaps differences are amplified by normalization. Additional explanation of the survival rate should be given, but I recommend showing all data as %age survival, this could be done in supplementary if authors like.
4. In many of the graphs, control genotype is not explained. A table of genotypes for each figure panel would clarify this and more importantly, control should be close of a match in background to the experimental condition to exclude affects of genetic background to survival rate. This means expressing of a control RNAi from a similar genetic background, or expressing of an control molecule for overexpression experiments.

Minor Criticisms
1. S.D. is preferable to S.E.M. for the error bars to see the data spread better.
2. For Figure 1G, H and 2E, G, it is not clear whether a single Z-section or a projection. This should be written in legend. Pictures look much more strong different than graphs.
3. It is not clear to me what the difference is between Figure 2L and Figure S2A - both measure GFP in the hemolymph after ectopic expression of either NetB-GFP or control GFP in the disc. The difference shown in Figure S2A seems more small than what Figure S2A shows and the images in Figure 2J,K suggest. This should be explained.
4. In figure 1I, the values appear normalized but the Y-axis indicates mean intensity. The Y-axis should be corrected. A preferable change would be to report mean intensity values without normalization.
5. The authors say that there is no cachexia in the eye disc larvae, but do not show this. Some examination of the fat body or muscle intactness should be included. Similarly, it is not mentioned whether cachexia phenotypes are present in Rasv12 gut model - this should be determined if the authors want to say that carnitine metabolism has a broad function in survival independent of cachexia.
6. In the discussion, several sentences could be re-edited. "Couple local neurogenesis and systemic metabolism"-any independent evidence that they are coupled, why neurogenesis rather than pathfinding? "If our findings are applicable to humans, inhibition of Netrin signaling in cancer patients may kill two birds with one stone, by improving the systemic symptom as well as by suppressing local tumorigenesis." - can the authors explain data that links fruit fly or human Netrin to local tumorigenesis?

Referee #3:

This manuscript describes that a ligand/receptor pair (NetB/Unc-5) that normally functions in axon guidance during development accounts for the lethality of tumours that are genetically induced in the fly eye. Because eye tissue is not required for viability, the authors investigated whether tumour-derived NetB signalling may trigger tumour-extrinsic mechanisms that result in lethality.

Through a series of tissue-specific gain- and loss-of-function experiments, the authors put forward a model whereby Netrin B secreted from the tumour binds to its Unc-5 receptor in fat body (akin to adipose tissue) to suppresses carnitine biosynthesis, critical for acetyl-CoA generation and systemic metabolism. The authors repeat some of these experiments in an adult gut tumour model with similar consequences, which lead them to suggest that carnitine generation in the fat body is also important in this context.

This is an unexpected and exciting finding. Whilst a priori it may not make sense for a tumor to kill its host through this mechanism, the authors speculate that inter-organ NetB signalling may have evolved to couple local neurogenesis with systemic metabolism, which is an intriguing idea. There are some loose ends that I feel need tightening, but I am generally positive about this manuscript

MAJOR ISSUES

1. Does the lethality phenotype (and systemic NetB) really emanate from the visual system tumours? GMR-Gal4 has been shown to be expressed in other tissues besides the eye disc (see PMID: 22911584). Can the authors rule out contributions from these other tissues?

2. Is the NetB/unc-5 effect eye disc specific? The authors use the gut model to illustrate the wider significance of their findings, but they only assess the effect of the gut tumour on TMHLE/carnitine. Presumably this effect is not mediated through NetB/Unc-5? This needs to be clarified. If the effects are eye disc-specific, one could argue this is a peculiar finding that is not even widely applicable across other fly tumours. To mitigate this criticism and to explore the wider significance of their findings from a different angle, the authors may want to explore the idea that this is a mechanism that couples neurogenesis with systemic metabolism (and which becomes dysregulated in tumors).

3. Is Unc-5 the only relevant receptor? The author test other Netrin receptors (fra, Dscam1) in the context of TMHLE expression but do they impact the lethality of the GMR-Rasv12 tumours?

4. What is the link between InR and NetB/Unc5? Why do both result in the same fat body phenotype? The cartoon in Fig 4h suggests that InR might impact Unc-5 levels but, unless I missed it, this is not shown. Does Unc-5 signalling affect InR expression or vice versa?

SPECIFIC COMMENTS

1. The authors state in the manuscript that "NetB inhibition in the Ras-transformed eye disc reversed the Ras-dependent TMLHE downregulation n the fat body (Fig 3d)." This figure does show the reversion but not the Ras-dependent TMLHE downregulation relative to controls; this needs to be provided.

2. When targeting the fat body, why did the authors use CG-Gal4 for some experiments and FB-Gal4 for others?

3. Table 1 was useful, but primary references for published Drosophila stocks should be included.

4. The finding that TMLHE knockdown in the fat body does not affect survival in flies with not tumours is an important negative control; I suggest that it is moved from the supplement to the main figures.

We would like to thank the editor and reviewers for their interest and enthusiasm in our manuscript. We spent half a year to experimentally address almost all comments. Because of their insightful comments and advice, now the manuscript improved tremendously. We hope this extensively revised manuscript will satisfy all the reviewers. Following is a point-by-point response.

Referee #1:

Okada M, et al seek to find how tumorkines may impact tumor-induced animal health deterioration and survival using flies. Expression of oncogenic RasV12 in differentiating cells of the eye part of the eye-antennal disc leads to a malformed (rough) eye due to developmental abnormalities. Despite the fact that all larvae reach the pupal stage with normal timing, only some hatch to adulthood. The lack of hatching is defined by the authors as survival from the oncogenic insult. Herein, lies my main concern. It is unclear whether this survival is similar to adult survival or is a particularity of the pupal stage which has specific energetic (non-feeding state), gross morphogenetic alterations and growth processes.

In this framework, 20 induced potential tumorkines were identified by RNASeq. One of these genes, netrin-B, is induced also at the protein levels and knockdown of the netrin-B expression improved hatching rate when knocked down in the tumor. Overexpression of netrin-B alone from the eye-antennal disc is sufficient to cause lethality (lack of hatching) showing that NetrinB is both required and adequate to inhibit hatching. Surprisingly, both endogenous NetrinB and tagged NetrinB from expressed in the eye disc are secreted systemically and accumulate in the Fat Body upon RasV12 overexpression only. Knocking down the Unc-5 netrin receptor (Unc-5) in the fat body, but not other netrin receptors (frazzled or Dscam1) increases survival, suggesting that netrin impacts the fat body directly to induced lethality. Increased survival is also seen when Insulin signaling in the fat body is reduced and this allowed the authors to identify levels of TMLHE as a critical enzyme for tumor stress survival using RNASeq and genetics. TMLHE takes part in generating carnitine which is known for its role in fatty acid import to mitochondria , oxidation and energy generation. How TMLHE expression is controlled by NetrinB-Unc-5 and Insulin signaling remains unknown. It is also unclear whether reduced fatty acid oxidation in the fat body is what causes lethality in the pupal stage. The main limitation of the manuscript is that the experiments addressing whether this applies outside of the pupal stage for more "conventional" survival is not completed.

The novelty of this exciting manuscript lies in the identification NetrinB-Unc5 signaling in controlling remote metabolism affecting life span.The manuscript is clearly written, and experiments are generally well designed according to the standard of the field.

Comments to authors:

General: Please provide source data, numbers of repeats etc for all graphs and figures. It is not possible to fully appreciate and evaluate the data and

experimentation without it. The following comments are made assuming that the data are correctly obtained and processed. Please also deposit the RNASeq source and ppm data, so that they can be evaluated.

We created a table that includes all the plotted data and information of statistical analyses performed in this manuscript. NGS data have been deposited in the DDBJ Sequence Read Archive (DRA) (accession number: DRA015648 and DRA015649).

The tumor model(s): The authors define survival as the capacity to exit the pupal stage to adulthood (hatching). As a first approximation, it is unclear how pupal lethality reflects tumor-induced health deterioration in animals as such. The pupal stage is a particular non-feeding state where much of the animal organs are partially broken down and new tissues are formed. The death susceptibility could therefore be due to a developmental or energetic demand specific to this particular developmental stage or processes.

We thank the reviewer for this important comment. We employed *GMR-Ras*^{V12} larvae as a model that enables us to induce dysplasia in a local tissue and to manipulate genes of interest in remote organs in order to examine the systemic effects of the oncogenic stress. However, as the reviewer pointed out, the pupal stage in which most *GMR-Ras*^{V12} animals die exhibits a particular metabolic state. To exclude the possibility that the death susceptibility could be due to a developmental or energetic demand specific to the pupal stage, in this revised manuscript, we characterized details of organismal death in larvae and adults. We found that Ras promotes expression of NetrinB in both wing disc in larvae and gut in adults (Fig 5a-b and f-h). In both situations, NetB inhibition suppresses Ras-induced lethality and NetB expression enhances the lethality (Fig 5c, EV7a, and 5i), suggesting that although the pupal situation should definitely affect organismal metabolism, Ras-Netrin signaling is a relatively general mechanism that affects organismal viability in other situations such as larvae and adults. We explain more details of these data below.

Could the authors experimentally address whether the survival of the hatched tumor-carrying GMR-RasV12-escapers is reduced relative to control animals?

We collected the *GMR-Ras*^{V12} escapers and assessed whether *GMR-Ras*^{v12} had a negative impact on adult lifespan. We observed a significant decrease in female and male lifespan of *GMR-Ras*^{V12} flies compared to control flies (Fig EV1l-m).

To partially amend this problem, the authors use an adult RasV12-driven gut tumor model initiated from stem cells (Fig S5). It is, however, still unclear how similar the situations are. Is NetB induced in RasV12 gut tumor cells in adults? Please perform immunostaining of NetB. Does NetB knockdown in RasV12 gut tumors increase survival? Similarly, unc-5 fat body knockdown should increase survival. If this applies, it will strengthen the role of NetB-Unc5-TMLHE in reducing health and survival apart from the particular situation of the pupal stage.

We thank the reviewer for pointing this out. As kindly suggested by the reviewer, we measured *NetB* using the gut tissue from control (*esg* ^{ts}>+) and *esg* ^{ts}>*Ras* ^{V12} flies. RT-qPCR revealed that the gut of *esg* ^{ts}>*Ras* ^{V12} flies upregulates *NetB* mRNA (Fig 5e). Consistent with the RT-qPCR data, the gut of *esg* ^{ts}>*Ras* ^{V12} flies also has higher levels of NetB protein (Fig 5f-h). We then assessed whether *NetB* inhibition in gut tumors had a positive impact on organismal viability. Inhibition of *NetB* in gut tumors lowered lethality (Fig 5i). On the other hand, ectopic expression of *NetB* in the gut induced lethality of *esg* ^{ts}>*Ras* ^{V12} flies (Fig EV7q).

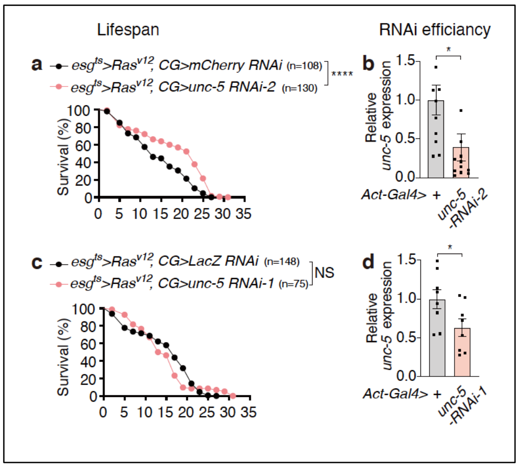

We also inhibited the *unc-5* in the fat body of *esg* ^{ts}>*Ras* ^{V12} flies. Knockdown of *unc-5* in the fat body using a line from Bloomington *Drosophila* stock center enhanced survival of *esg* ^{ts}>*Ras* ^{V12} flies (Fig. a). However, knockdown of *unc-5* in the fat body using a line from the Vienna *Drosophila* stock center did not extend lifespan of *esg* ^{ts}>*Ras* ^{V12} flies (Fig. c). The difference in the survival rate could be due to the RNAi efficiency, since the Bloomington stock demonstrated better knockdown than the VDRC stock (Fig b, d). But, we cannot exclude a possibility that other NeB receptors such as fra or Dscam1 might be involved in organismal health of adult animals. Since further extensive experiments and clarification are necessary to conclude the role for unc-5 and other NetB receptors in adults, we prefer not to include these results in the current manuscript.

Besides the adult gut tumor, in this revised report, we overexpressed oncogenic Ras in the wing disc using the *hh-Gla4* driver (Fig 5a-b). Ras induces NetB expression in non-neuronal wing epithelia (Fig 5a-b). In the context of oncogenic Ras expression in the wing disc, approximately half of the *nub*>*Ras* ^{V12} animals die at the larval stage (Fig 5c-d, EV7a). Importantly, we found that inhibition of *NetB* reverses the organismal lethality at the larval stages induced by Ras ^{V12}. On the other than, NeB expression enhances organismal lethality. Taken together, these data suggest that NetB could be a general Ras-induced tumorkine that could affect organismal fitness regardless of the developmental stages.

Fig1g-h. NetB has been shown be involved in neuronal differentiation and axonal guidance. Is NetB a general stress-induced tumorkine outside the neural system? This could easily be adressed by NetB immunostaining of animals with hs-flp induced RasV12 expressing MARCM clones ahead of the morphogenic furrow or antennal part. Other tissues can also be scored (brain, wing disc, gut,..)

In the revised manuscript, we demonstrate that Ras induces NetB expression in the non-neuronal tissues such as larval wing disc and adult midgut (Fig 5a-b and f-h). This indicates that induction of NetB by Ras is a relatively general phenomenon.

Fig1 c,d,e. Is it any particular reason why relative survival rate rather than absolute survival (hatching) as in Fig 1a?

Let us explain why we show relative survival rather than the absolute survival. The survival rate of even control fluctuates depending on food conditions, temperatures, humidity and seasons. Thus, we normalize survival by the control when we use the *GMRRas*^{V12} flies. We think that in a system where control data fluctuate biologically, it's more informative to normalize data by control values. We used this analysis in a previous publication (Nishida et al., 2021). To provide a rough idea of the survival rate, we showed the survival rate as a percentage only in Fig.1a. In this revised manuscript, we included all the raw data as the source data to ensure transparency.

5. Fig 2. A minor but interesting question is whether Netrin-B localization to the fat body depends on Unc-5 expression. This would suggest a ligand trapping mechanism. A simple immunostaining of NetB with or w/o FB-Gal4 > Unc-5-RNAi should answer this

This is definitely an interesting question but might be difficult to clarify. While revising this paper and conducing experiments to address another reviewer's comments, we found that even inhibition of other NetB receptors such as fra and dscam affects resistance to oncogenic stress. Since inhibition of these receptors doesn't affect TMLHE (Fig 3e), we think that NetB may mediate other mechanisms through these receptors. Although we have such an impression, these observations need more extensive clarification, which requires tremendous effort and time. If other receptors also trap NetB on the fat body, it's difficult to appreciate whether Unc5 affects trapping of NetB in the fat body. Again, we absolutely agree that this reviewer's question is an interesting topic from a cell biological point of view, but needs intense clarification and experimentation. So, we would like to pursue the ligand-trapping mechanism by Unc5 and potentially other receptors in our future project.

6. Fig3. Genetic experiments support a model whereby RasV12 remotely suppresses TMLHE mRNA levels in the fat body through Netrin-B-Unc-5 and that this is reversed upon suppression of the Insulin signaling pathway. It remains unknown how TMLHE may be regulated transcriptionally downstream of the receptor levels. Fig3 B,C. Does knockdown of the insulin receptor in the fat body lead to higher levels of TMLHE transcription alone?

We thank the reviewer for this important comment. We inhibited insulin receptor in the fat body of normal flies and then measured *TMLHE* mRNA expression in the fat body. In the absence of *GMR-Ras*^{V12}, inhibition of insulin receptor in the fat body does not affect *TMLHE* expression in the fat body (Fig EV5k). As the reviewer rightfully pointed out, we still don't know how NetB regulates TMLHE or how insulin signals cross talk with NetB signaling. We definitely plan to pursue this mechanistic details in our future study. We clarified this point in discussion.

7. Fig 4. Fig 4 a-e. The authors show convincingly using genetics that TMLHE is limiting in the fat body for survival to adulthood only for animals with RasV12 expression in the eye (gmr-RasV12). This is suggested to be related to carnitine

biosynthesis needed for fatty acid import to mitochondria for energy generation. This is supported by the results that show increased survival by supplying carnitine or an acetyl-CoA precursor increase survival. However, if this model is correct, two predictions should hold true:

1. Knocking down crucial components of the carnitine shuttle/fatty acid import should have the same reduced survival, like knockdown of Cpt1 or Cpt2 in the fat body. For instance. Gmr-RasV12, CG-Gal4 >Cpt1 (or Cpt2)-RNAi versus CG-Gal4>Cpt1 (or Cpt2)-RNAi (see PMID: 33371457)

As kindly suggested by the reviewer, we tested whether manipulation of *CPT2* in the fat body could affect organismal death. Knockdown of *CPT2* in the fat body of *GMR-Ras^{V12}* flies using independent *CPT2* RNAi lines significantly decreased their survival, suggesting that inhibition of *CPT2* in the fat body makes animals more sensitive to Ras-transformation. In the absence of *GMR-Ras^{V12}*, inhibition of *CPT2* in the fat body is not sufficient to induce organismal death. We have now included figures in Figure EV6i-j.

2. Knocking down TMLHE in the fat body should be limiting for ATP levels, possibly only in the Gmr-RasV12 background. I encourage the authors to elaborate on these experiments.

We measured the amount of ATP to examine whether manipulation of *TMLHE* in the fat body could affect ATP level. Knockdown of *TMLHE* in the fat body of *GMR-Ras^{V12}* flies significantly decreased ATP levels. In contrast to the survival rate, inhibition of *TMLHE* in the fat body also reduced ATP levels even in the absence of *GMR-Ras^{V12}*. This result suggests *GMR-Ras^{V12}* animals become more sensitive to a decrease of ATP. We have now included the data in Figure EV6h.

These data and *CPT2* inhibition data suggest that the fat body produces and consumes carnitine. These data prompted us to examine the amount of carnitine in tissues more carefully. We collected the tissues from L3 larvae of control (OegonR) and measured the amount of carnitine in the whole body, fat body, and hemolymph (Fig. e). Interestingly, we found a low amount of carnitine in the hemolymph. Instead, more than half of the total amount of carnitine exists in the fat body, suggesting that carnitine might be produced and consumed in the same tissue, at least in *Drosophila*.

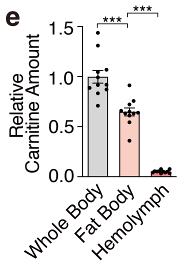

8. Fig 4g. To try and address whether TMLHE extends to affect death susceptibility other than at the pupal stage, which may have particular developmental or energetic demands, the authors resort to an adult gut tumor model driven by RasV12 in the stem cells. Esg>RasV12 animals display more than 50% reduction of life span compared to control. Knocking down TMLHE in the fat body further decreases this life span. However, control animals with TMLHE knockdown alone in the fat body is lacking to see if the reduction is additive or dependent on tumor presence.

We apologize for not having included important controls. We carried out a new experiment that included following, appropriate controls.

1. *esg*^{ts}>+, *LacZ RNAi* (Normal flies, Control *RNAi*)
2. *esg*^{ts}>+, *TMLHE RNAi* (Normal flies, *TMLHE RNAi*)
3. *esg*^{ts}>*Ras*^{V12}, *LacZ RNAi* (Tumor flies, Control *RNAi*)
4. *esg*^{ts}>*Ras*^{V12}, *TMLHE RNAi* (Tumor flies, *TMLHE RNAi*)

Consistent with the *GMR-Ras*^{V12} data, knockdown of *TMLHE* in the adult fat body had a negative impact on viability of *esg*^{ts}>*Ras*^{V12} flies. However, inhibition of *TMLHE* in the fat body of normal flies (*esg*^{ts}>+) is not sufficient to induce organismal death. Rather, we observed a slightly positive impact on lifespan of normal flies. We replaced the old figure with a new one (Fig 5l).

Further, it needs to be established whether NetB is expressed in Esg>RasV12 gut cells and whether NetB knockdown in the fat body is sufficient to extend life span under oncogenic stress. It may of course, be other factors involved in reducing adult health and life span than NetB, like the recently published Upd-Dome blood-brain barrier disruption in another tumor model (PMID: 34496290), so a complete rescue of reduced life span is not expected.

As explained in the previous comment, we measured NetB mRNA and protein using the gut tissue from control (*esg*^{ts}>+) and *esg*^{ts}>*Ras*^{V12} flies. Both NetB mRNA and protein are highly upregulated in the gut of *esg*^{ts}>*Ras*^{V12} flies. Furthermore, inhibition and expression of *NetB* in gut tumors had a positive and negative impact on organismal viability respectively.

Referee #2:

Okada et al present a new system that studies oncogenic stress caused by a Drosophila RasV12 eye disc, and identify a new organ-organ signaling mechanism mediated by NetrinB that controls metabolism in the fat body. This molecule has been previously functioning in local signaling for neuron pathfinding. It is therefore a surprising finding that this paper reports and genetic and feeding experiments demonstrate it elegantly. Larvae with RasV12 eye discs make pupae, but infrequently eclose. The authors show this can be rescued by blocking NetB signaling to fat body. The authors identify carnitine metabolism downstream of NetB/Unc-5 through a secondary genetic screen for genes that are differentially regulated with inhibition of insulin signaling in the fat body with RasV12 eye discs. The authors screen point to TMHLE as being transcriptionally downregulated by NetB action, leading to reduced carnitine biosynthesis in the pupae. The authors are able to rescue eclosure rates by changing TMHLE expression or with carnitine supplementation in diet. The authors attempt to extend these findings to a RasV12 gut tumor model in the adult fly.

There is an exciting hypothesis presented in this paper that Netrins have a conserved function in organ-organ signaling and control metabolism in addition to neuronal connections. The studies the authors cite in human cancer elicit an

attractive proposal that these findings can be broadly applied to understand death from oncogenic stress. However, I have concern that the data could also be interpreted as phenomenological to fly metamorphosis, which would narrow the impact. To achieve the broader relevance that the authors proposed in discussion, I would recommend some additions to the manuscript outlined below.

1. The authors use eclosure rate as measure of survival. It is not clear to me that failure of metamorphosis is the same as death of animal due to oncogenic stress. Development of the pupae into a adult requires significant and maybe very specific regulation of organ-organ signaling and metabolism that could be derailed by manipulations of the authors. The paper should write about this point rather than just using the word survival.

We agree with the reviewer's interpretation. Thus, in this revised text, we also exploited and characterized additional systems, larval wing disc and adult intestinal stem cells. In both tissues, Ras induces NetrinB expression and inhibition/expression of NetB increases/decreases organismal fitness. We clarified this point. Please also see our detailed response to the reviewer 1.

2. To make a case for NetB signaling mechanism being applied generally to oncogenic stress killing, the authors should extend the interesting studies in the adult RasV12 gut model, most of which should be easy:

A. Is NetB upregulated in the adult gut tumor? This should be easily ascertained from RNA or protein measurement or from microarray dataset (Tsuda-Sakurai et al 2020).

As explained in the response to the reviewer#1, the gut of *esg* ^{ts}>*Ras* ^{V12} flies has higher levels of NetB mRNA and NetB protein. We have now included figures in Figure 5f-h.

B. The experiments of Fig. 4G are missing important controls to show that RNAi of TMLHE in normal flies does not make them ill and die quicker, and a control RNAi expressed in the tumor model does not change survival.

We apologize for not having included important controls. As explained in the response to the reviewer#1, we carried out a new experiment that included appropriate controls. We replaced the old figure with a new one (Fig 5l).

C. The authors could test whether carnitine or acetyl COA supplementation extends life of gut tumor flies.

We thank this reviewer for this helpful suggestion. We orally supplemented carnitine to *esg* ^{ts}>*Ras* ^{V12} flies. Consistent with the *GMR-Ras* ^{V12} flies, carnitine administration enhanced survival of *esg* ^{ts}>*Ras* ^{V12} flies. We have now included a figure in Fig. EV7r.

D. Additionally, measure carnitine levels in adult fat body with and without tumor.

As helpfully suggested, we measured the amount of carnitine in *esg* ^{ts}>*Ras*^{V12} flies under *Ras*^{V12}-tumor induction for 2, 10, and 20 days using the LexA system. We found a decrease of carnitine in *esg* ^{ts}>*Ras*^{V12} flies under *Ras*^{V12}-tumor induction for 10 and 20 days compared to control (*esg* ^{ts}>+). In addition, TMLHE mRNA is also decreased in *esg* ^{ts}>*Ras*^{V12} flies compared to control. We have now included these data in Figure 5j and k.

E. NetB-Unc5 pathway could be directly tested between the gut tumor and the fat body through NetB RNAi in the tumor, and/or Unc5 RNAi in the fat body.

As explained in the response to the reviewer1, inhibition of *NetB* in gut tumors had a positive impact on organismal viability. We have now included the data in Fig. 5i.

3. Measurements for larval survival assays are sometimes presented as a %age survival and other times as rate normalized to the control. It is difficult to compare the effect between these two different measurement units. I do not understand why for graphs with normalized survival rate, the data points bin into discreet groups rather than having more of a continuous spread.

Regarding the rationale to normalize the data, please see our comment to the reviewer 1. Briefly, we believe this representation of data, which are not affected by fluctuation of control, will be more useful for readers. We also included all the raw data in the source file. Let us also explain how we calculate the survival rate of flies. First, the number of adult flies of each genotype that could eclose was recorded. Then, survival rates were calculated by dividing the number of survivors with the expected number of larvae of each genotype placed in each vial. Thus, a single dot in the graph represents the survival rate obtained from a single vial. We made multiple attempts and obtained the results that included many dots.

I am surprised by much larger survival rescue with Unc-5 heterozygotes (Figure 2N) compared to NetB RNAi (Figure 1C). If they are acting in the same signaling pathway, I would expect the effect to be less disparate, but perhaps differences are amplified by normalization. Additional explanation of the survival rate should be given, but I recommend showing all data as %age survival, this could be done in supplementary if authors like.

As explained in the comment to Reviewer#1, the survival rate fluctuates on different food conditions, temperatures, and humidity. Therefore, we normalize survival by the control when we use the *GMRRas*^{V12} flies.

Let us explain the survival rate of unc-5 heterozygotes (Figure 2n) compared to NetB RNAi (Figure 1c). Absolute survival for each strain is as follows. Figure 2n: *GMR-Ras*^{v12}*, unc-5/+:* 69.2 ± 4.3% (Mean ± SEM), Figure 1c: *GMR-Ras*^{v12}*, GMR-Gal4>NetB RNAi-1:* 84.8 ± 7.8%, *GMR-Ras*^{v12}*, GMR-Gal4>NetB RNAi-2:* 55.5 ± 5.2%. As the reviewer rightly interpreted, the difference in relative survival rates between unc-5 heterozygotes (Figure 2n) and NetB RNAi (Figure 1c) could be due to the fluctuation of survival in control flies of each experiment. We also note that knockdown efficiency of NetB RNAis is not so great (Fig EV2b-c), which also

complicates interpretation. In addition, we also have preliminary data that other NetB receptors may also be involved in the systemic response to the Ras dysplasia, although these receptors do not affect TMLHE expression (Fig 3e, EV5l-m). This could also explain why there is a difference between NetB and unc5 inhibition. Overall, we agree with the reviewer and included both relative survival and absolute survival data (%) in the Source Data.

4. In many of the graphs, control genotype is not explained. A table of genotypes for each figure panel would clarify this and more importantly, control should be close of a match in background to the experimental condition to exclude affects of genetic background to survival rate. This means expressing of a control RNAi from a similar genetic background, or expressing of an control molecule for overexpression experiments.

We agree and have revised the method in the manuscript. We also re-took appropriate control RNAi or expressing control molecules from a similar genetic background as controls (Fig. 1e, 5c, 5d, 5i, 5l, and EV7q). For TRiP RNAi lines, matching Attp2 and Attp40 lines were used as the control accordingly (Fig. 4c, 4d, EV4b, EV4d, EV6b, EV6c, EV6e, and EV6g). For other RNAi lines, we used OregonR or irrelevant control RNAis as control. In case we cannot re-do everything, we confirmed that UAS-RNAi expression does not affect the survival rate compared to OregonR and w- control. For the adult tumor model, we generated *esg-lexA::HG, lexAop-GFP, tub-Gal80^{ts}, CG-Gal4* flies (referred as *esg^{ts}>+*) as an appropriate control for *esg-lexA::HG, lexAop-RasV12, lexAop-GFP, tub-Gal80ts, CG-Gal4* flies (referred as *esg^{ts}>Ras^{V12}*). We have now included the data in Fig EV2.

Minor Criticisms

1. S.D. is preferable to S.E.M. for the error bars to see the data spread better.

Since we use a scatter plot with a bar for the graph, the data spread is already represented. To give insight on reliability of mean in each condition, we combined the scatter plot and SEM.

2. For Figure 1G, H and 2E, G, it is not clear whether a single Z-section or a projection. This should be written in legend. Pictures look much more strong different than graphs.

We apologize for not having included the details of image acquisition. Pictures in Figure 1g, h and Figure 2e, g are single confocal z-section images. We have revised the figure legend accordingly. When we quantify the intensity measurement of fluorescent signals, the background signal is also inevitably measured, making the quantitative difference look smaller than perception obtained in the pictures.

3. It is not clear to me what the difference is between Figure 2L and Figure S2A - both measure GFP in the hemolymph after ectopic expression of either NetB-GFP or control GFP in the disc. The difference shown in Figure S2A seems more small than what Figure S2A shows and the images in Figure 2J,K suggest. This should be explained.

We apologize for the lack of description. We used microscopy or spectrophotometry to measure GFP signals in the hemolymph. In figure 2I, we quantified the intensity of GFP signals of pictures taken by a microscope. In figure EV4a, we measured the intensity of GFP signals with a Nanodrop spectrophotometer. The differences in GFP intensity are due to differences in the sensitivity of methods. However, in both measurement cases, higher levels of GFP signal in the hemolymph were observed in flies with *GMR> NetB-GFP* rather than *GMR>GFP*.

4. In figure 1I, the values appear normalized but the Y-axis indicates mean intensity. The Y-axis should be corrected. A preferable change would be to report mean intensity values without normalization.

We followed this reviewer's suggestion. We have modified the Y axis to mean intensity values without normalization.

5. The authors say that there is no cachexia in the eye disc larvae, but do not show this. Some examination of the fat body or muscle intactness should be included. Similarly, it is not mentioned whether cachexia phenotypes are present in Rasv12 gut model - this should be determined if the authors want to say that carnitine metabolism has a broad function in survival independent of cachexia.

This was a great suggestion. In this revised manuscript, we investigated whether animals carrying *Ras*^{V12} -transformed tissue display cachexia symptoms such as bloating phenotype, muscle or fat body degeneration, and trehalose increase (hyperglycemia), which are often induced by more aggressive tumors.
No *GMR-Ras*^{V12} third-instar larvae show bloating syndrome. We also found that no *GMR-Ras*^{V12} third-instar larvae show muscle or fat body degeneration. Consistent with the Lipi-Red staining, we found no difference in triglyceride measurements between control and *GMR-Ras*^{V12} third-instar larvae. Furthermore, we measured the concentration of trehalose, the primary circulating sugar composed of two alpha-glucose, which can increase in the tumor-bearing flies (Kwon et al., 2015; Santabarbara-Ruiz & Leopold, 2021). However, no *GMR-Ras*^{V12} third-instar larvae and pupae show an increase in trehalose concentration compared to the control ones. Rather, a small or no decrease in the trehalose concentration was observed. These data suggest that *GMR-Ras*^{V12} third-instar larvae do not display obvious cachexia symptoms. We have now included these data in figure EV1 d-k.

We also examined cachexia symptoms using a tumor model in the adult gut (*esg*^{ts}>*Ras*^{V12}). We do not find bloating syndrome in *esg*^{ts}>*Ras*^{V12} flies, instead we often observed an abdominal shrinkage phenotype. We also examined the ovary, which degenerates progressively due to cachexia in the gut tumor-bearing flies (Figueroa-Clarevega & Bilder, 2015; Kwon et al., 2015). We measuered the ovary size as previously described (Zhang et al., 2023) and observed significantly smaller ovaries in *esg*^{ts}>*Ras*^{V12} flies compared to control flies. Half of *esg*^{ts}>*Ras*^{V12} flies showed an ovary atrophy. However, no *esg*^{ts}>*Ras*^{V12} flies show muscle or fat body degeneration compared to control. These data suggest that some *esg*^{ts}>*Ras*^{V12} flies

exhibited some phenotype, known as the cachexia phenotype in the *Drosophila* tumor model. We have now included these data in figure EV7 h-p.

6. In the discussion, several sentences could be re-edited. "Couple local neurogenesis and systemic metabolism"-any independent evidence that they are coupled, why neurogenesis rather than pathfinding? "If our findings are applicable to humans, inhibition of Netrin signaling in cancer patients may kill two birds with one stone, by improving the systemic symptom as well as by suppressing local tumorigenesis." - can the authors explain data that links fruit fly or human Netrin to local tumorigenesis?

As helpfully suggested by the reviewer, we modified "neurogenesis" to "neuronal pathfinding". We also toned down our speculation to clarify that this part is speculative discussion, which discusses a potential relevance of our fly study to medicine.

Referee #3:

1. Does the lethality phenotype (and systemic NetB) really emanate from the visual system tumours? GMR-Gal4 has been shown to be expressed in other tissues besides the eye disc (see PMID: 22911584). Can the authors rule out contributions from these other tissues?

We thank the reviewer for this important comment. As the reviewer correctly pointed out, W-Z Li *et al.* reported that *GMR-Gal4* is expressed not only in third-instar larva eye discs but also in wing imaginal discs, leg discs, brain, and trachea (Li, Li, Zheng, Zhang, & Xue, 2012). Therefore, we re-analyzed the expression pattern of two independent *GMR-Gal4* lines (2nd chromosome and 3rd chromosome) in six tissues (eye discs, wing imaginal discs, leg discs, brain, trachea, and salivary gland) using G-TRACE system, which enables detection of current (RFP) and past (GFP) expression.

We observed RFP and GFP expression in the eye discs, trachea, and salivary gland but not in wing imaginal discs, leg discs or brain. In addition, the *GMR-Gal4* (3rd chromosome) expression pattern completely overlaps the independent *GMR-Gal4* (2nd chromosome) expression pattern.

We then examined the expression pattern of NetB protein in *GMR-Ras*^{V12} and found that the eye disc of *GMR-Ras*^{V12} flies has higher levels of NetB protein, but not the trachea or salivary gland. We also observed NetB expression in the ventral nerve cord, consistent with previous findings (Kang et al., 2019). Taken together, only the eye disc displays overlapped expression of *GMR-gal4* and NetB protein, suggesting likely that NetB in the eye disc is critical for organismal death, at least in the *GMR-Gal4* line used in our experimental system. We have now included these in Figure EV3a-c.

2. Is the NetB/unc-5 effect eye disc specific? The authors use the gut model to illustrate the wider significance of their findings, but they only assess the effect of the

gut tumour on TMHLE/carnitine. Presumably this effect is not mediated through NetB/Unc- 5? This needs to be clarified. If the effects are eye disc-specific, one could argue this is a peculiar finding that is not even widely applicable across other fly tumours. To mitigate this criticism and to explore the wider significance of their findings from a different angle, the authors may want to explore the idea that this is a mechanism that couples neurogenesis with systemic metabolism (and which becomes dysregulated in tumors).

Please see our response to the Reviewers #1 and #2. Briefly, we observed Ras induces expression of NetB in both the wing disc and the intestinal stem cells. Inhibition and expression of NetB in both tissues enhances and worsens survival over the Ras dysplasia respectively.

3. Is Unc-5 the only relevant receptor? The author test other Netrin receptors (fra, Dscam1) in the context of TMHLE expression but do they impact the lethality of the GMR-Rasv12 tumours?

This is a good point. We thank the reviewer for this important comment. As kindly suggested by the reviewer, we examined the survival rate of *GMR-Ras*^{V12} flies after fra and Dscam-1 knock-down in the fat body. Interestingly, knockdown of fra and Dscam-1 in the fat body of *GMR-Ras*^{V12} flies enhances survival (Fig. f). Since only *unc-5* but not fra or Dscam-1 knockdown affected *TMLHE* expression (Fig 3e, Fig EV5l-m), fra and Dscam-1 might mediate other mechanisms for increasing survival over oncogenic stress besides unc-5-mediated TMLHE regulation. Since we plan to pursue how these receptors might regulate organismal survival in the future, we prefer not to include these data in the current manuscript.

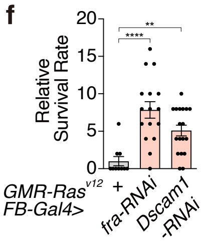

4. What is the link between InR and NetB/Unc5? Why do both result in the same fat body phenotype? The cartoon in Fig 4h suggests that InR might impact Unc-5 levels but, unless I missed it, this is not shown. Does Unc-5 signalling affect InR expression or vice versa?

We apologize for the lack of description. In Fig. EV5j, we show that the fat body-specific inhibition of insulin receptor decreases *unc-5* mRNA expression in the fat body of *GMR-Ras*^{V12} flies. Since inhibition of unc-5 increases TMLHE expression in the fat body, the insulin receptor in the fat body might regulates organismal death by regulating TMLHE through the *unc-5.* However, we agree with the reviewer that we still don't know how NetB signaling regulates TMLHE and also how exactly crosstalk of NetB and insulin signaling occurs. We'd like to pursue the mechanistic details in our future endeavor. We clarified this point in discussion.

SPECIFIC COMMENTS

1. The authors state in the manuscript that "NetB inhibition in the Ras-transformed eye disc reversed the Ras-dependent TMLHE downregulation n the fat body (Fig

3d)." This figure does show the reversion but not the Ras-dependent TMLHE downregulation relative to controls; this needs to be provided.

This is important, and we apologize for the lack of data. We carried out a new experiment that included appropriate control. RT-qPCR demonstrated that *TMLHE* mRNA is decreased in the fat body of *GMR-Ras*^{V12} flies and increased by NetB knockdown in the eye disc. We replaced the previous data with the new one (Fig 3d).

2. When targeting the fat body, why did the authors use CG-Gal4 for some experiments and FB-Gal4 for others?

We initially used *CG-Gal4* as the fat body specific Gal4-driver. Since the *Cg-Gal4* driver is expressed in fat body and hemocytes, we shifted to use *FB-Gal4.* Please note that we verify almost all data using both *CG-Gal4* and *FB-Gal4* drivers.

3. Table 1 was useful, but primary references for published Drosophila stocks should be included.

We agree and have revised the Table 1 accordingly.

4. The finding that TMLHE knockdown in the fat body does not affect survival in flies with not tumours is an important negative control; I suggest that it is moved from the supplement to the main figures.

We agree and moved the figure to Fig. 4d.

We hope that our explanation and changes are satisfactory. Again, we appreciate the comments and suggestions by the reviewers to help us improve the paper.

Figueroa-Clarevega, A., & Bilder, D. (2015). Malignant Drosophila tumors interrupt insulin signaling to induce cachexia-like wasting. *Dev Cell, 33*(1), 47-55. doi:10.1016/j.devcel.2015.03.001

Kang, H., Zhao, J., Jiang, X., Li, G., Huang, W., Cheng, H., & Duan, R. (2019). Drosophila Netrin-B controls mushroom body axon extension and regulates courtship-associated learning and memory of a Drosophila fragile X syndrome model. *Mol Brain, 12*(1), 52. doi:10.1186/s13041-019-0472-1

Kwon, Y., Song, W., Droujinine, I. A., Hu, Y., Asara, J. M., & Perrimon, N. (2015). Systemic organ wasting induced by localized expression of the secreted insulin/IGF antagonist ImpL2. *Dev Cell, 33*(1), 36-46. doi:10.1016/j.devcel.2015.02.012

Li, W. Z., Li, S. L., Zheng, H. Y., Zhang, S. P., & Xue, L. (2012). A broad expression profile of the GMR-GAL4 driver in Drosophila melanogaster. *Genet Mol Res, 11*(3), 1997-2002. doi:10.4238/2012.August.6.4

Nishida, H., Okada, M., Yang, L., Takano, T., Tabata, S., Soga, T., . . . Yoo, S. K. (2021). Methionine restriction breaks obligatory coupling of cell proliferation and death by an oncogene Src in Drosophila. *Elife, 10*. doi:10.7554/eLife.59809

Santabarbara-Ruiz, P., & Leopold, P. (2021). An Oatp transporter-mediated steroid sink promotes tumor-induced cachexia in Drosophila. *Dev Cell, 56*(19), 2741-2751 e2747. doi:10.1016/j.devcel.2021.09.009

Zhang, Y., Ting, C. Y., Yang, S., Reich, J., Fru, K., & Lilly, M. A. (2023). Wdr59 promotes or inhibits TORC1 activity depending on cellular context. *Proc Natl Acad Sci U S A, 120*(1), e2212330120. doi:10.1073/pnas.2212330120

1st Revision - Editorial Decision 27th Mar 2023

Dear Sakan,

Thank you for submitting a revised version of your manuscript. Your study has now been seen by all original referees, who find that their previous concerns have been addressed and now recommend publication of the manuscript. There remain only a few mainly editorial points that have to be addressed before I can extend formal acceptance of the manuscript:
1. Please extend the discussion on the experimental caveats and tone down the conclusions as requested by reviewer #2. Please also add the information on the genetic background of the used fly lines.
1. Please upload the main and EV figures as individual production quality figure files in the .eps, .tif,or .jpg format (one file per figure).
2. We can accommodate up to five EV figures. Please consider either moving two of the EV figures to the Appendix, or transforming, for example Figures EV5 and EV7 into main figures.
3. Please update references according to The EMBO Journal style - where there are more than 10 authors on a paper, the first 10 should be listed, followed by 'et al.' Please see further information here:
https://www.embopress.org/page/journal/14602075/authorguide#referencesformat
4. Papers published in The EMBO Journal are accompanied online by a 'Synopsis' to enhance discoverability of the manuscript. It consists of A) a short (1-2 sentences) summary of the findings and their significance, B) 3-4 bullet points highlighting key results and C) a synopsis image that is 550x300-600 pixels large (width x height, jpeg or png format). You can either show a model or key data in the synopsis image. Please note that the image size is rather small and that text needs to be readable at the final size. Please send us this information together with the revised manuscript.

Please let me know if you have any questions regarding any of these points. You can use the link below to upload the revised files.

Thank you again for giving us the chance to consider your manuscript for The EMBO Journal. I look forward to receiving the final version.

With best regards,

Ieva

Ieva Gailite, PhD
Senior Scientific Editor
The EMBO Journal
Meyerhofstrasse 1
D-69117 Heidelberg
Tel: +4962218891309
i.gailite@embojournal.org

Further information is available in our Guide For Authors: https://www.embopress.org/page/journal/14602075/authorguide

We realize that it is difficult to revise to a specific deadline. In the interest of protecting the conceptual advance provided by the work, we recommend a revision within 3 months (25th Jun 2023). Please discuss the revision progress ahead of this time with the editor if you require more time to complete the revisions.

Referee #1:

Okada M, et al seek to find how tumorkines may impact tumor-induced animal health deterioration and survival using flies. In this work, they identified NetrinB as being induced in RAsV12-driven ontogenically transformed cells as inducing premature death. The initial work was performed in larval/pupal stages using a reduced hatching rate as a measure. In response to several reviewers' concerns, the authors now extend these findings to adult flies. They show that NetrinB is expressed in response to RasV12 transformation in other tissues (gut, wing disc)in addition to the eye antennal disc and enacts reduced life span. This is further distinct from cancer cachexia phenotypes earlier described in that it does not obviously affect muscle or adipose tissue but does reduce the growth of the ovary. In the previous version of the manuscript, they further identified the Unc-5 netrin receptor (Unc-5)in the fat body, as being responsible for the reduced life span. Moreover, they showed that TMLHE, an enzyme required for carnitine-mediated fatty acid transport and beta-oxidation is reduced and seems partially responsible for the effects.

In the revision, they extend this analysis to cpt2 downstream of TMLHE providing further evidence to the idea that reduced FAO in the fat body contributes to death. In keeping with this hypothesis, lower levels of ATP are measured in the fat body upon TMLHE knockdown or in GMR-RasV12 expressing animals. As requested, the authors now provide primary data and improved the data visibility and explanations.

Although it is now clear that not all NetrinB detrimental effects are mediated through Unc5/TMLHE, it is beyond reasonable doubt that it is a major mechanism. It places NetrinB as a new tumorkine in the expanding negative physiological effects instigated by tumors with more to be explored.

All my major concerns raised by the first submission are adequately addressed.

Referee #2:

The authors have done a commendable job responding to the critiques. I support publication following responses to the below:
To me the case for a general Ras to Netrin to TMLHE humoral pathway with oncogenic impact is still less strong than the text implies. The effect size of TMLHE RNAi and carnitine supplementation in the adult gut model look small, although p values reach significance. In the larval wing model, it is not explored when or why the animals do not pupate, and again one of the NetB RNAi has a quite small effect on the phenotype. Does the significant but tiny change in EV7r make the authors comfortable with the abstract's broad statement that "Supplementation of carnitine or acetyl-CoA inhibits oncogenic stress-induced organismal death'"? Answering these questions is not required for this manuscript, but I would suggest that the authors caveat their discussion and also revise their abstract to reflect the strength of the data.

A lack of clarity about the control genotypes remain, which are especially critical for the lifespans. The authors appear aware of the importance of this issue, as expressed in their response to previous comment 4. But the specific information is still lacking. A table of genotypes used in each figure panel would clarify this and has become broadly adopted in the field. Many of the figures still use "+" to refer to the controls, which leaves ambiguity for whether and which control RNAi lines were used. Others specify a LacZ RNAi. It is not clear why LacZ RNAi was used for Figure 5 whereas TRIP attp2 and attp40 lines were used for Figures 4, EV4 and EV6. The reference for the LacZ line from Table 1 indicates that it was generated by p-element insertion, which can have quite dirty genetic backgrounds. It is not impossible that such a mechanism could account for the small effect of Fig. 5L. I emphasize that although this manuscript may not have the best controls each and every time, the targeting of multiple points in the pathway as well as carnitine feeding as an alternative assay to genetics does make a good case overall for the authors' conclusions.
"Ectopic expression of the GFP-tagged NetB, but not control GFP, in the eye disc led to existence of GFP signals in both the hemolymph and the fat body (Fig 2e-I, EV4a), providing further evidence that NetB secreted by the eye disc humorally relays the signal to the fat body". Again because of the incomplete genotypes including Table 1 it is not clear if this is a standard cytoplasmic GFP or a GFP tagged with a secretory sequence. Presumably it is the latter, because the former would be a silly control. If it is not, this needs to be repeated or removed.

Figure EV7q: does NetBGFP overexpression from the adult gut have an effect alone on WT ---why was this done only in the context of RasV12? It should parallel Fig. 1E.
Figure 1e: GMR>NetB overexpression: do they die as larvae or pupae?
I remain surprised that NetB protein is so detectable in fat body, and that effects of a 50% reduction of transcript are so strong, when NetB upregulation by RasV12 is so limited: just 50% increase from portions of two imaginal discs in the entire animal.
Figure 5I: ideally NetB RNAi with esg-ts alone should be shown, though unlikely that would extend WT lifespan, so it is not worth delaying the paper just for this experiment.

Referee #3:

The authors have addressed my concerns. The finding that this Netrin is also induced in other tumour contexts broadens the interest of the authors' finding. They have also addressed other specific concerns, so I would be happy to see this manuscript published in its current form.

Dear Ieva,

Please see our response to your comments below.

1. Please extend the discussion on the experimental caveats and tone down the conclusions as requested by reviewer #2. Please also add the information on the genetic background of the used fly lines.

We modified our discussion and toned down our conclusion as suggested by the reviewer 2. We also provide a complete table that includes exact genotypes of all experiments (Dataset EV3).

1. Please upload the main and EV figures as individual production quality figure files in the .eps, .tif,or .jpg format (one file per figure).

We followed this instruction.

2. We can accommodate up to five EV figures. Please consider either moving two of the EV figures to the Appendix, or transforming, for example Figures EV5 and EV7 into main figures.

We reduced the number of EV figures down to five by combining some figures.

3. Please update references according to The EMBO Journal style - where there are more than 10 authors on a paper, the first 10 should be listed, followed by 'et al.' Please see further information here: https://www.embopress.org/page/journal/14602075/authorguide#referencesformat

We modified the citation style.

4. Papers published in The EMBO Journal are accompanied online by a 'Synopsis' to enhance discoverability of the manuscript. It consists of A) a short (1-2 sentences) summary of the findings and their significance, B) 3-4 bullet points highlighting key results and C) a synopsis image that is 550x300-600 pixels large (width x height, jpeg or png format). You can either show a model or key data in the synopsis image. Please note that the image size is rather small and that text needs to be readable at the final size. Please send us this information together with the revised manuscript.

We submitted the synopsis.

We provide point-by-point responses below.

Referee #1:
Okada M, et al seek to find how tumorkines may impact tumor-induced animal health deterioration and survival using flies. In this work, they identified NetrinB as being induced in RAsV12-driven ontogenically transformed cells as inducing premature death. The initial work was performed in larval/pupal stages using a reduced hatching rate as a measure. In response to several reviewers' concerns, the authors now extend these findings to adult flies. They show that NetrinB is expressed in response to RasV12 transformation in other tissues (gut, wing disc)in addition to the eye antennal disc and enacts reduced life span. This is further distinct from cancer cachexia phenotypes earlier described in that it does not obviously affect muscle or adipose tissue but does reduce the growth of the ovary. In the previous version of the manuscript, they further identified the Unc-5 netrin receptor (Unc-5)in the fat body, as being responsible for the reduced life span. Moreover, they showed that TMLHE, an enzyme required for carnitine-mediated fatty acid transport and beta-oxidation is reduced and seems partially responsible for the effects. In the revision, they extend this analysis to cpt2 downstream of TMLHE providing further evidence to the idea that reduced FAO in the fat body contributes to death. In keeping with this hypothesis, lower levels of ATP are measured in the fat body upon TMLHE knockdown or in GMR-RasV12 expressing animals. As requested, the authors now provide primary data and improved the data visibility and explanations.

Although it is now clear that not all NetrinB detrimental effects are mediated through Unc5/TMLHE, it is beyond reasonable doubt that it is a major mechanism. It places NetrinB as a new tumorkine in the expanding negative physiological effects instigated by tumors with more to be explored.

All my major concerns raised by the first submission are adequately addressed.

We thank the reviewer for his/her appreciation of our work.

Referee #2:
The authors have done a commendable job responding to the critiques. I support publication following responses to the below:
To me the case for a general Ras to Netrin to TMLHE humoral pathway with oncogenic impact is still less strong than the text implies. The effect size of TMLHE RNAi and carnitine supplementation in the adult gut model look small, although p values reach significance. In the larval wing model, it is not explored when or why the animals do not pupate, and again one of the NetB RNAi has a

quite small effect on the phenotype. Does the significant but tiny change in EV7r make the authors comfortable with the abstract's broad statement that "Supplementation of carnitine or acetyl-CoA inhibits oncogenic stress-induced organismal death"'? Answering these questions is not required for this manuscript, but I would suggest that the authors caveat their discussion and also revise their abstract to reflect the strength of the data.

We appreciate the reviewer's constructive comments. We agree with the reviewer's comment and have revised the abstract and the discussion as suggested.

Abstract:
We changed the text "Supplementation of carnitine or acetyl-CoA inhibits oncogenic stress-induced organismal death" to "Supplementation of carnitine or acetyl-CoA ameliorates organismal health under oncogenic stress."

Discussion:
We also toned down our conclusion by not using the word "organismal death".

A lack of clarity about the control genotypes remain, which are especially critical for the lifespans. The authors appear aware of the importance of this issue, as expressed in their response to previous comment 4. But the specific information is still lacking. A table of genotypes used in each figure panel would clarify this and has become broadly adopted in the field. Many of the figures still use "+" to refer to the controls, which leaves ambiguity for whether and which control RNAi lines were used. Others specify a LacZ RNAi. It is not clear why LacZ RNAi was used for Figure 5 whereas TRIP attp2 and attp40 lines were used for Figures 4, EV4 and EV6. The reference for the LacZ line from Table 1 indicates that it was generated by p-element insertion, which can have quite dirty genetic backgrounds. It is not impossible that such a mechanism could account for the small effect of Fig. 5L. I emphasize that although this manuscript may not have the best controls each and every time, the targeting of multiple points in the pathway as well as carnitine feeding as an alternative assay to genetics does make a good case overall for the authors' conclusions.

We thank this reviewer for this helpful suggestion. We created a table of genotypes used in each figure panel and uploaded it as Dataset EV3.

Regarding RNAi controls, whenever possible, for TRiP RNAi lines, matching Attp2 and Attp40 lines were used as control. For other RNAi lines, we used OregonR or irrelevant control RNAis as control. In case we could not re-do all

experiments, we confirmed that UAS-RNAi expression does not affect the survival rate compared to OregonR and w- control. At least in a context of organismal survival upon Ras expression, we did not find any difference among w1118, OregonR, LacZ RNAi, mCherry RNAi (TRiP line), Luciferase RNAi (TRiP line) when we crossed our tester to these (EV1n).

"Ectopic expression of the GFP-tagged NetB, but not control GFP, in the eye disc led to existence of GFP signals in both the hemolymph and the fat body (Fig 2e-l, EV4a), providing further evidence that NetB secreted by the eye disc humorally relays the signal to the fat body". Again because of the incomplete genotypes including Table 1 it is not clear if this is a standard cytoplasmic GFP or a GFP tagged with a secretory sequence. Presumably it is the latter, because the former would be a silly control. If it is not, this needs to be repeated or removed.

Here we are using normal GFP as a control, not GFP with a secretory sequence. There are two purposes of using GFP as a negative control. One is to demonstrate that GMR-gal4 does not promote expression of Gal4 in the fat body. Second is to show that NetB-GFP secreted from the eye disc can reach the eye disc in a specific manner. For the first purpose, cytoplasmic GFP suffices. For the second purpose, we agree that the secretory GFP can work as a better, specific negative control for secreted NetB, but cytoplasmic GFP should still function as a negative control compared to having nothing. In this case, especially for the first reason, we humbly reason it'd be better to keep GFP control rather than completely removing it from the paper.

Figure EV7q: does NetBGFP overexpression from the adult gut have an effect alone on WT ---why was this done only in the context of RasV12? It should parallel Fig. 1E.

In this manuscript, we believe that the main component is the data with GMR>Ras. The data in ISCs are complementary for this main part. So, all data with ISCs were obtained in a context of Ras. In that sense, it's less comprehensive than the part of GMR-Ras. We sincerely hope the reviewer would generously understand this.

Figure 1e: GMR>NetB overexpression: do they die as larvae or pupae?

Ectopic expression of NetB in the eye disc of normal animals induced lethality at the early pupal stage. We clarified this in text.

I remain surprised that NetB protein is so detectable in fat body, and that effects of a 50% reduction of transcript are so strong, when NetB upregulation by RasV12 is so limited: just 50% increase from portions of two imaginal discs in the entire animal.

This is certainly an interesting point. Although many genes exhibit a higher increase in expression than NetB, inhibiting those genes does not suppress oncogenic stress-induced organismal death. As one of the NetB receptors (Unc-5) is upregulated four-fold by the ectopic expression of Ras^{V12}, upregulating Unc-5 might amplify the effect of NetB at the organismal level.

Figure 5I: ideally NetB RNAi with esg-ts alone should be shown, though unlikely that would extend WT lifespan, so it is not worth delaying the paper just for this experiment.

We appreciate the reviewer's enthusiastic curiosity and generous understanding.

Referee #3:
The authors have addressed my concerns. The finding that this Netrin is also induced in other tumour contexts broadens the interest of the authors' finding. They have also addressed other specific concerns, so I would be happy to see this manuscript published in its current form.

We would like to thank the referee for his/her valuable and constructive comments on our manuscript.

2nd Revision - Editorial Decision — 20th Apr 2023

Dear Sakan,

Thank you for addressing the final editorial issues, and I apologise for the delay in communicating the decision due to the Easter holidays and the resulting backlog. I am now pleased to inform you that your manuscript has been accepted for publication.

Before we can forward your manuscript to our publishers, there remain a couple of editorial points that need addressing:
1. I am very sorry to say that I have only now noticed the extremely short introductory paragraph of the manuscript. Please include a more detailed overview of studies reporting on systemic effects of tumour growth on organismal metabolism and fitness, especially in the Drosophila model, and on the biological roles of Netrin family molecules, also including the studies that report on their elevated expression in cancer.
2. I would like to propose a couple of changes in the article title, abstract and synopsis. I have also written a short blurb that will accompany the title of your manuscript in our online table of contents. Please take a look at the text below and in the attached manuscript text file and let me know if any corrections are necessary.

Title:
Oncogenic stress-induced Netrin is a humoral signaling molecule that reprograms systemic metabolism in Drosophila

Blurb:
Ras-induced tumors in Drosophila reduce organismal fitness by Netrin secretion-dependent suppression of carnitine biosynthesis in the fat body.
Synopsis:
NetrinB is a secreted protein that acts as a regulator of cell migration and an axon guidance cue. The current study identifies a role for NetB as a humoral signaling molecule that mediates systemic effects of local oncogenic stress on organismal metabolism in Drosophila.
- Ras-induced dysplastic cells upregulate and secrete NetrinB.
- Inhibition of either NetrinB from the transformed tissue or its receptor in the fat body suppresses oncogenic stress-induced organismal death.
- NetrinB from the dysplastic tissue suppresses carnitine biosynthesis in the fat body.
- Supplementation of carnitine or acetyl-CoA improves fly survival under oncogenic stress.

Please note that it is EMBO Journal policy for the transcript of the editorial process (containing referee reports and your response letter) to be published as an online supplement to each paper. If you do NOT want this, you will need to inform the Editorial Office via email immediately. More information is available here:
https://www.embopress.org/page/journal/14602075/authorguide#transparentprocess

Your manuscript will be processed for publication in the journal by EMBO Press. Manuscripts in the PDF and electronic editions of The EMBO Journal will be copy edited, and you will be provided with page proofs prior to publication. Please note that supplementary information is not included in the proofs.

You will be contacted by Wiley Author Services to complete licensing and payment information. The required 'Page Charges Authorization Form' is available here: https://www.embopress.org/pb-assets/embo-site/tej_apc.pdf - please download and complete the form and return to embopressproduction@wiley.com

EMBO Press participates in many Publish and Read agreements that allow authors to publish Open Access with reduced/no publication charges. Check your eligibility: https://authorservices.wiley.com/author-resources/Journal-Authors/open-access/affiliation-policies-payments/index.html

Should you be planning a Press Release on your article, please get in contact with embojournal@wiley.com as early as possible, in order to coordinate publication and release dates.

If you have any questions, please do not hesitate to call or email the Editorial Office. Thank you for this contribution to The EMBO Journal and congratulations on a successful publication!

Best wishes,

Ieva

Ieva Gailite, PhD
Senior Scientific Editor
The EMBO Journal

Meyerhofstrasse 1
D-69117 Heidelberg
Tel: +4962218891309
i.gailite@embojournal.org

EMBO Press Author Checklist

Corresponding Author Name: Sa Kan Yoo, Morihiro Okada	
Journal Submitted to: EMBO	
Manuscript Number: EMBOJ-2022-111383	

USEFUL LINKS FOR COMPLETING THIS FORM
The EMBO Journal - Author Guidelines
EMBO Reports - Author Guidelines
Molecular Systems Biology - Author Guidelines
EMBO Molecular Medicine - Author Guidelines

Reporting Checklist for Life Science Articles (updated January
This checklist is adapted from Materials Design Analysis Reporting (MDAR) Checklist for Authors. MDAR establishes a minimum set of requirements in transparent reporting in the life sciences (see Statement of Task: 10.31222/osf.io/9sm4x). Please follow the journal's guidelines in preparing your
Please note that a copy of this checklist will be published alongside your article.

Abridged guidelines for figures
1. Data
The data shown in figures should satisfy the following conditions:
- the data were obtained and processed according to the field's best practice and are presented to reflect the results of the experiments in an accurate and unbiased manner.
- ideally, figure panels should include only measurements that are directly comparable to each other and obtained with the same assay.
- plots include clearly labeled error bars for independent experiments and sample sizes. Unless justified, error bars should not be shown for technical
- if n<5, the individual data points from each experiment should be plotted. Any statistical test employed should be justified.
- Source Data should be included to report the data underlying figures according to the guidelines set out in the authorship guidelines on Data

2. Captions
Each figure caption should contain the following information, for each panel where they are relevant:
- a specification of the experimental system investigated (eg cell line, species name).
- the assay(s) and method(s) used to carry out the reported observations and measurements.
- an explicit mention of the biological and chemical entity(ies) that are being measured.
- an explicit mention of the biological and chemical entity(ies) that are altered/varied/perturbed in a controlled manner.
- the exact sample size (n) for each experimental group/condition, given as a number, not a range;
- a description of the sample collection allowing the reader to understand whether the samples represent technical or biological replicates (including how many animals, litters, cultures, etc.).
- a statement of how many times the experiment shown was independently replicated in the laboratory.
- definitions of statistical methods and measures:
  - common tests, such as t-test (please specify whether paired vs. unpaired), simple χ2 tests, Wilcoxon and Mann-Whitney tests, can be unambiguously identified by name only, but more complex techniques should be described in the methods section;
  - are tests one-sided or two-sided?
  - are there adjustments for multiple comparisons?
  - exact statistical test results, e.g., P values = x but not P values < x;
  - definition of 'center values' as median or average;
  - definition of error bars as s.d. or s.e.m.

Please complete ALL of the questions below.
Select "Not Applicable" only when the requested information is not relevant for your study.

Materials

Newly Created Materials	Information included in the manuscript?	In which section is the information available? (Reagents and Tools Table, Materials and Methods, Figures, Data Availability Section)
New materials and reagents need to be available; do any restrictions apply?	Yes	method

Antibodies	Information included in the manuscript?	In which section is the information available? (Reagents and Tools Table, Materials and Methods, Figures, Data Availability Section)
For **antibodies** provide the following information:<br>- Commercial antibodies: RRID (if possible) or supplier name, catalogue number and or/clone number<br>- Non-commercial: RRID or citation	Yes	method

DNA and RNA sequences	Information included in the manuscript?	In which section is the information available? (Reagents and Tools Table, Materials and Methods, Figures, Data Availability Section)
**Short novel DNA or RNA including primers, probes**: provide the sequences.	Yes	method and table

Cell materials	Information included in the manuscript?	In which section is the information available? (Reagents and Tools Table, Materials and Methods, Figures, Data Availability Section)
**Cell lines:** Provide species information, strain. Provide accession number in repository **OR** supplier name, catalog number, clone number, and/**OR** RRID.	Not Applicable	
**Primary cultures:** Provide species, strain, sex of origin, genetic modification status.	Not Applicable	
Report if the cell lines were recently **authenticated** (e.g., by STR profiling) and tested for mycoplasma contamination.	Not Applicable	

Experimental animals	Information included in the manuscript?	In which section is the information available? (Reagents and Tools Table, Materials and Methods, Figures, Data Availability Section)
**Laboratory animals or Model organisms:** Provide species, strain, sex, age, genetic modification status. Provide accession number in repository **OR** supplier name, catalog number, clone number, **OR** RRID.	Yes	method
**Animal observed in or captured from the field:** Provide species, sex, and age where possible.	Not Applicable	
Please detail **housing and husbandry conditions**.	Yes	method

Plants and microbes	Information included in the manuscript?	In which section is the information available? (Reagents and Tools Table, Materials and Methods, Figures, Data Availability Section)
**Plants:** provide species and strain, ecotype and cultivar where relevant, unique accession number if available, and source (including location for collected wild specimens).	Not Applicable	
**Microbes:** provide species and strain, unique accession number if available, and source.	Not Applicable	

Human research participants	Information included in the manuscript?	In which section is the information available? (Reagents and Tools Table, Materials and Methods, Figures, Data Availability Section)
If collected and within the bounds of privacy constraints report on age, sex and gender or ethnicity for all study participants.	Not Applicable	

Core facilities	Information included in the manuscript?	In which section is the information available? (Reagents and Tools Table, Materials and Methods, Figures, Data Availability Section)
If your work benefited from core facilities, was their service mentioned in the acknowledgments section?	Not Applicable	

Design

Study protocol	Information included in the manuscript?	In which section is the information available? (Reagents and Tools Table, Materials and Methods, Figures, Data Availability Section)

If study protocol has been **pre-registered, provide DOI in the manuscript**. For clinical trials, provide the trial registration number **OR** cite DOI.	Not Applicable	
Report the **clinical trial registration number** (at ClinicalTrials.gov or equivalent), where applicable.	Not Applicable	

Laboratory protocol	Information included in the manuscript?	In which section is the information available? (Reagents and Tools Table, Materials and Methods, Figures, Data Availability Section)
Provide DOI OR other citation details if **external detailed step-by-step protocols** are available.	Not Applicable	

Experimental study design and statistics	Information included in the manuscript?	In which section is the information available? (Reagents and Tools Table, Materials and Methods, Figures, Data Availability Section)
Include a statement about **sample size** estimate even if no statistical methods were used.	Yes	method
Were any steps taken to minimize the effects of subjective bias when allocating animals/samples to treatment (e.g. **randomization procedure**)? If yes, have they been described?	Yes	method
Include a statement about **blinding** even if no blinding was done.	Yes	method
Describe **inclusion/exclusion criteria** if samples or animals were excluded from the analysis. Were the criteria pre-established?<br><br>If sample or data points were omitted from analysis, report if this was due to attrition or intentional exclusion and provide justification.	Not Applicable	
For every figure, are **statistical tests** justified as appropriate? Do the data meet the assumptions of the tests (e.g., normal distribution)? Describe any methods used to assess it. Is there an estimate of variation within each group of data? Is the variance similar between the groups that are being statistically compared?	Yes	method

Sample definition and in-laboratory replication	Information included in the manuscript?	In which section is the information available? (Reagents and Tools Table, Materials and Methods, Figures, Data Availability Section)
In the figure legends: state number of times the experiment was **replicated** in laboratory.	Yes	legend
In the figure legends: define whether data describe **technical or biological replicates**.	Yes	legend

Ethics

Ethics	Information included in the manuscript?	In which section is the information available? (Reagents and Tools Table, Materials and Methods, Figures, Data Availability Section)
Studies involving **human participants**: State details of **authority granting ethics approval** (IRB or equivalent committee(s), provide reference number for approval.	Not Applicable	
Studies involving **human participants**: Include a statement confirming that **informed consent** was obtained from all subjects and that the experiments conformed to the principles set out in the WMA Declaration of Helsinki and the Department of Health and Human Services Belmont Report.	Not Applicable	
Studies involving **human participants:** For publication of **patient photos**, include a statement confirming that consent to publish was obtained.	Not Applicable	
Studies involving experimental **animals**: State details of **authority granting ethics approval** (IRB or equivalent committee(s), provide reference number for approval. Include a statement of compliance with ethical regulations.	Not Applicable	
Studies involving **specimen and field samples:** State if relevant **permits** obtained, provide details of authority approving study; if none were required, explain why.	Not Applicable	

Dual Use Research of Concern (DURC)	Information included in the manuscript?	In which section is the information available? (Reagents and Tools Table, Materials and Methods, Figures, Data Availability Section)
Could your study fall under dual use research restrictions? Please check biosecurity documents and list of **select agents and toxins** (CDC): https://www.selectagents.gov/sat/list.htm	Not Applicable	
If you used a select agent, is the security level of the lab appropriate and reported in the manuscript?	Not Applicable	
If a study is subject to dual use research of concern regulations, is the name of the **authority granting approval and reference number** for the regulatory approval provided in the manuscript?	Not Applicable	

Reporting

The MDAR framework recommends adoption of discipline-specific guidelines, established and endorsed through community initiatives. Journals have their own policy about requiring specific guidelines and recommendations to complement MDAR.

Adherence to community standards	Information included in the manuscript?	In which section is the information available? (Reagents and Tools Table, Materials and Methods, Figures, Data Availability Section)
State if relevant guidelines or checklists (e.g., **ICMJE, MIBBI, ARRIVE, PRISMA**) have been followed or provided.	Not Applicable	
For **tumor marker prognostic studies,** we recommend that you follow the **REMARK** reporting guidelines (see link list at top right). See author guidelines, under 'Reporting Guidelines'. Please confirm you have followed these guidelines.	Not Applicable	
For **phase II and III randomized controlled trials**, please refer to the **CONSORT** flow diagram (see link list at top right) and submit the CONSORT checklist (see link list at top right) with your submission. See author guidelines, under 'Reporting Guidelines'. Please confirm you have submitted this list.	Not Applicable	

Data Availability

Data availability	Information included in the manuscript?	In which section is the information available? (Reagents and Tools Table, Materials and Methods, Figures, Data Availability Section)
Have **primary datasets** been deposited according to the journal's guidelines (see 'Data Deposition' section) and the respective accession numbers provided in the Data Availability Section?	Yes	DDBJ Sequence Read Archive (DRA) (accession number: DRA015648 and DRA015649).
Were **human clinical and genomic datasets** deposited in a public access-controlled repository in accordance to ethical obligations to the patients and to the applicable consent agreement?	Not Applicable	
Are **computational models** that are central and integral to a study available without restrictions in a machine-readable form? Were the relevant accession numbers or links provided?	Not Applicable	
If publicly available data were reused, provide the respective **data citations in the reference list**.	Not Applicable	
